# Uncovered Diversity of a Predominantly Andean Butterfly Clade in the Brazilian Atlantic Forest: a Revision of the Genus *Praepedaliodes* Forster (Lepidoptera: Nymphalidae, Satyrinae, Satyrini)

**DOI:** 10.1007/s13744-017-0543-x

**Published:** 2017-10-02

**Authors:** T W Pyrcz, A V L Freitas, P Boyer, F M S Dias, D R Dolibaina, E P Barbosa, L M Magaldi, O H H Mielke, M M Casagrande, J Lorenc-Brudecka

**Affiliations:** 1 Entomology Dept, Institute of Zoology and Biomedical Research, Jagiellonian Univ, Kraków, Poland; 2 Nature Education Centre, Jagiellonian Univ, ul. Gronostajowa 5, 30-387 Kraków, Poland; 3 Depto de Biologia Animal and Museu de Zoologia, Instituto de Biologia, Univ Estadual de Campinas, Campinas, Brasil; 4 7, Lotissement l’Horizon, Le Puy Sainte Réparade, France; 5 Lab de Estudos de Lepidoptera Neotropical, Depto de Zoologia, Univ Federal do Parana, Curitiba, Brasil; 6 McGuire Center for Lepidoptera and Biodiversity, Univ of Florida, Gainesville, FL USA

**Keywords:** Brazil, molecular phylogeny, morphology, new species, Pronophilina, taxonomy

## Abstract

The genus *Praepedaliodes* Forster, 1964, the only representative of the mega-diverse mostly Andean *Pedaliodes* complex lineage in the Brazilian Atlantic Forest, is revised. Prior to this study, four species were known, *P. phanias* (Hewitson, 1862), *P. granulata* (Butler, 1868), *P. amussis* (Thieme, 1905) and *P. exul* (Thieme, 1905). Here, a further six are described, all from SE Brazil, expanding to 10 the number of species in this genus. Lectotypes are designated for *P. phanias*, *P. granulata* and *P. amussis*. The genus is most diverse in the Serra da Mantiqueira (São Paulo, Rio de Janeiro, Minas Gerais) and in the Serra Geral (Paraná, Santa Catarina) with seven species occurring in both ranges. *Praepedaliodes phanias* is the most widespread species and the only one found in the western part of the Atlantic Forest; only this species and *P. duartei* Dias, Dolibaina & Pyrcz n. sp. occurring to near sea level. Other species, *P. zaccae* Dolibaina, Dias & Pyrcz n. sp., *P. francinii* Freitas & Pyrcz n. sp., *P. sequeirae* Pyrcz, Dias & Dolbaina n. sp., *P. landryi* Pyrcz & Freitas n. sp. and *P. pawlaki* Pyrcz & Boyer n. sp. are strictly montane and the highest species richness is reached at 1400–1800 m. One species, *P. sequeirae* n. sp., is a narrow endemic found only at timberline in the Agulhas Negras massif above 2300 m. Immature stages are described for two species, *P. phanias* and *P. landryi* n. sp. Molecular data (barcode region of cytochrome oxidase, subunit I) and adult morphology, including male and female genitalia, support the genus as monophyletic, belonging to a predominantly Andean clade of the *Pedaliodes* Butler, 1867 complex. Morphological evidences, in particular female genitalia comparative analysis, indicate the genera *Physcopedaliodes* Forster, 1964 and *Panyapedaliodes* Forster, 1964 as possibly the closest relatives to *Praepedaliodes*. Molecular data are inconclusive in this respect.

## Introduction

The subfamily Satyrinae is a cosmopolitan group of brush-footed butterflies (Nymphalidae) with at least 2500 known species, and the highest taxonomic diversity reported in the tribe Satyrini is found in the neotropical region (Peña *et al* 2006, Pyrcz 2010). Neotropical Satyrini segregate into two apparently monophyletic subtribes (Peña *et al* 2006): (1) the predominantly lowland Euptychiina (with the notable exception of the strictly montane genus *Forsterinaria* Gray, 1973) and (2) the nearly exclusively montane Pronophilina (Pyrcz 2010). The status, tribal or subtribal, of some neotropical genera, such as *Manataria* Kirby, [1902], *Oressinoma* Doubleday, [1849], *Calisto* Hübner, [1823] and *Diaphanos* Adams & Bernard, 1981 in particular, remains a debatable issue (see Viloria 1994, 2003, Peña *et al* 2006). Pronophilina, with some 45–50 genera and over 550 described species (and at least 50–80 more identified and to be described), largely dominate in terms of species richness and abundance in the cloud forests of the Andes and the peripheral mountain ranges of Sierra Nevada de Santa Marta, the Venezuelan Cordillera de la Costa and the Sierra de Talamanca in Costa Rica and Panama (Adams 1985, Pyrcz 2010, Pyrcz *et al* 2013). The Euptychiina, with over 400 described species, constitute the most speciose group of Satyrinae in the Amazonian basin lowlands and the Andean foothills (Lamas 2004, Freitas *et al* 2015). Only six genera of Pronophilina occur exclusively outside from the Andes—*Drucina* Butler, 1872 in Mesoamerica, *Arhuaco* Adams & Bernard, 1977 in the Sierra Nevada de Santa Marta and in Mesoamerica, *Protopedaliodes* Viloria & Pyrcz, 1994 in the Guyana Shield, and three genera confined to SE Brazil—*Foetterleia* Viloria, 2004, *Eteona* Doubleday, 1848 and *Praepedaliodes* Forster, 1964 (the latter two marginally penetrating into neighbouring Uruguay, Paraguay and Argentina). *Foetterleia* and *Eteona* are monobasic with remote, and still only superficially researched affinities with the Andean taxa, especially the latter one (Freitas 2002). Therefore, in the mountains of SE Brazil, the subtribe Pronophilina is severely underrepresented compared to the Andes, and even to Mesoamerica, and its ecological niche is taken over by the Euptychiina which show a huge generic and species diversity in the cloud forests of this region. Several genera of Euptychiina have undergone important adaptive radiations in the Brazilian Atlantic Forest, including *Moneuptychia* Forster, 1964 and *Carminda* Ebert & Dias, 1997 (Freitas 2007, Dias 2011, Freitas *et al* 2015).

The only genus of Pronophilina which has witnessed some radiation in the mountains of SE Brazil, *Praepedaliodes*, was erected by Forster (1964) in his monumental taxonomic work on neotropical Satyrinae, alongside many other genera separated from the species-rich (>200 species) *Pedaliodes* Butler, 1867 complex. *Praepedaliodes* was described based exclusively on male genital characters, and Forster (1964) pointed out the following diagnostic characters: (1) a stout uncus, (2) a short subuncus (i.e. “gnathos”), (3) a long and thin aedeagus and (4) characteristically shaped valves. So far, four species of *Praepedaliodes* have been described, three of which are known only from SE Brazil, namely *P. exul* (Thieme, 1905), *P. granulata* (Butler, 1868)*, P. amussis* (Thieme, 1905), and *P. phanias* (Hewitson, 1862), a widespread species also found in Uruguay, northern Argentina and eastern Paraguay. All four species of *Praepedaliodes* were described over a hundred years ago, with *P. phanias* and *P. granulata* in the nineteenth century and *P. exul* and *P. amussis* in early twentieth century (Thieme 1905). Such a timeframe might imply that the species catalogue of the genus *Praepedaliodes* is complete. The research carried out by the authors of this paper shows, however, that this is far from reality. Recent sampling and taxonomic studies reveal that the total number of extant species of *Praepedaliodes* is more than double than that previously thought. Most surprisingly, most of the newly identified species occur in the vicinity of large Brazilian cities, São Paulo, Curitiba and Rio de Janeiro, and have been found in museum drawers misidentified among the four previously described species. These discoveries emphasize how much work remains to be done on the butterflies of even apparently well-known faunal regions of South America. In this paper, a review of the taxonomy and distribution, and a preliminary examination of the relationships among the species within the genus *Praepedaliodes* are provided.

## Material and Methods

### Material

A total of 639 male and 212 female specimens of all ten extant species of *Praepedaliodes* were examined (Figs 1 to 13). Types of all the taxa described prior to this study were examined in MfN, NHMUK and ZSBS.

The acronyms of the consulted collections are as follows: **CLAM**: Alfred Moser collection, São Leopoldo, Rio Grande do Sul, Brazil; **DD**: Diego R. Dolibaina Collection, Curitiba, Paraná, Brazil; **DZRS**: Coleção Entomológica do Departamento de Zoologia, Universidade Federal do Rio Grande do Sul, Porto Alegre, Rio Grande do Sul, Brazil; **DZUP**: Coleção Entomológica Padre Jesus Santiago Moure, Departamento de Zoologia, Universidade Federal do Paraná, Curitiba, Paraná, Brazil; **MfN**: Museum für Naturkunde der Humboldt Universität, Berlin, Germany; **MIZPAN**: Muzeum i Instutut Zoologii Polskiej Akademii Nauk, Warszaw, Poland; **MNHG**: Museum of Natural History, Geneva, Switzerland; **MUSM**: Museo de Historia Natural, Universidad Nacional Mayor de San Marcos, Lima, Peru; **CEP-MZUJ**: Nature Education Centre, Jagiellonian University (formerly: Zoological Museum of the Jagiellonian University), Kraków, Poland; **MZUSP**: Museu de Zoologia, Universidade de São Paulo, São Paulo, Brazil; **NHMUK**: The Natural History Museum, London, United Kingdom; **OM**: Olaf H. H. Mielke Collection, Curitiba, Paraná, Brazil; **PBF**: Pierre Boyer Collection, Le Puy Sainte Réparade, France; **SMTD**: Senckenberg Museum für Tierkunde, Dresden, Germany; **UFRGS**: Universidade Federal do Rio Grande do Sul, Porto Alegre, Brazil; **ZSBS**: Zoologiche Sammlungen des Bayerlichen Staates, Münich, Germany; **ZUEC**: Museu de Zoologia Adão José Cardoso, Universidade Estadual de Campinas, Campinas, São Paulo, Brazil; **ZUEC-AVLF**: André V. L. Freitas collection, Universidade Estadual de Campinas, Campinas, São Paulo, Brazil.

### Field studies

Field studies were carried out by TP in SE Brazil in 2006 (São Paulo), 2013 (Minas Gerais, São Paulo, Rio Grande do Sul), 2014 (São Paulo, Minas Gerais, Rio de Janeiro) and 2015 (São Paulo, Paraná, Santa Catarina, Rio Grande do Sul), the latter in the company of PB, by AVLF (São Paulo, Minas Gerais, Rio de Janeiro, Espírito Santo and Bahia), and OHHM, DRD, MMC and FMSD (Minas Gerais, Rio de Janeiro, São Paulo, Paraná, Santa Catarina, Rio Grande do Sul) on several occasions. Standard sampling procedures were used, including entomological hand nets with various extensions, and van Someren-Rydon traps with dung of carnivorous animals, although these proved ineffective as an attractant for *Praepedaliodes* (*P. phanias* was observed to be attracted to fermented banana bait). Photographic documentation has been made of biotopes and individuals in natural habitats (Figs 14 to 16). Elevations above sea level were measured by SILVA altimeter, calibrated daily, and GPS for each collecting locality. Voucher specimens from the above field trips were deposited in DD, DZUP, MZUJ, OM, ZUEC and ZUEC-AVLF.

### Rearing

Fertile eggs were obtained from wild-captured females, which were confined in plastic bags along with leaves of several potential host-plants (species of small leaved bamboos) and put under a source of heat (40W incandescent lamp). The intense heat triggers in most females the behaviour of laying eggs. The eggs were laid on the leaves and/or scattered on the plastic bag. Larvae were reared in plastic containers cleaned daily, with fresh plant material provided every 2 or 3 days. Data were recorded on behaviour and development time for all stages (Figs 14, 15). Dry head capsules and pupal cases were retained in glass vials. Immature stages were fixed in Kahle-Dietrich solution when the number of specimens was sufficient. Voucher specimens of the immature stages were deposited at the ZUEC-AVLF. Measurements were taken and general aspects of morphology were observed using a Leica®MZ7.5 stereomicroscope equipped with a micrometric scale. Egg size is presented as height and diameter, and head capsule size is the distance between the most external ocelli (as in Freitas 2007, Freitas *et al* 2015). Terminology for early stages descriptions followed García-Barros & Martín (1995) for eggs and Stehr (1987) for larvae and pupae.

### Morphology

Male and female genitalia were removed from abdomens soaked in 10% KOH solution for 5–10 min. The same procedure was used for examination of head parts. Subsequently, abdomens were preliminarily cleaned out of soft tissue in water in order to expose genital parts. Female abdomens were stained in chlorazol black in order to identify soft genital parts. Dissected genitalia were cleaned using 90 and 95% ethanol solutions. A Nikon digital camera DS-Fi1 and an Olympus SZX9 stereomicroscope were used for taking pictures of the dissections, which were then processed in Combine ZP and Corel PHOTO-PAINT X3 programs to enhance focus and improve quality (Figs 5 to 12). Genital dissections were kept in glycerol vials pinned under corresponding specimens. Male genital terminology follows largely Klots (1956) and Razowski (1996). Female genital morphology follows Coutsis (1983) who was the first author who examined in detail female genitalia of Palearctic Satyrini, and suggested new terms for several morphological structures, including the subdivision into proximal, median and distal units. Wing preparation was performed by soaking in hot 10% KOH solution and subsequently by removing dorsal and ventral scales. Wing slides were photographed and preserved in glycerol (Fig 13).

### Genetic divergence and phylogenetic inference

Total genomic DNA was isolated from 58 individuals of *Praepedaliodes* species using Invisorb® Spin Tissue Mini Kit (STRATEC Molecular, Germany). The mitochondrial DNA (mtDNA) gene cytochrome oxidase subunit I (COI, 1499 bp) was sequenced, including the barcode region proposed by Hebert *et al* (2003), which is the 5′ portion of the COI (658 bp), according to published protocols (Wahlberg & Wheat 2008). Sequences were aligned with all sequences available for *Praepedaliodes* genus in Genbank, with 14 individuals as outgroups, 12 of which belonging to eight genera of the *Pedaliodes* complex, one belonging to the genus *Pseudomaniola* Röber, 1889 (a representative of another clade of the subtribe Pronophilina), and one belonging to the genus *Atlanteuptychia* Freitas, Barbosa & Mielke, 2013, a representative of the subtribe Euptychiina, considered as sister group of Pronophilina (Table 1).

**Table 1:** Species of *Praepedaliodes* and outgroups with code, sampling site data and GenBank accession numbers for COI sequences.

Code	Genus	Species	Country	Locality	Genbank code
YPH0191	*Atlanteuptychia*	*ernestina*	Brazil	São Paulo: Jundiaí (Serra do Japi)	KP994863
CP04-01	*Pseudomaniola*	*phaselis*	Peru	Junín	DQ338593
EUCD1	*Neopedaliodes*	*parrhoebia*	Ecuador	–	KT448680
YPH0538	*Panyapedaliodes*	*drymaea*	Colombia	La Cocha via Sibundoy	MF415602
YPH0539	*Panyapedaliodes*	*drymaea*	Colombia	La Cocha via Sibundoy	MF415603
CP07-51	*Parapedaliodes*	*parepa*	Peru	Lima	DQ338591
YPH0536	*Pedaliodes*	*phaedra*	Colombia	Purace	MF415600
YPH0537	*Pedaliodes*	*pilaloensis*	Colombia	Purace	MF415601
CP09-66	*Pedaliodes*	*ewelina*	Peru	AP, S.N. de Ampay, Laguna Uspacocha	DQ338856
YPH0535	*Physcopedaliodes*	*physcoa*	Colombia	Sierra Nevada de Santa Marta	MF415599
CP07-86	*Punapedaliodes*	*flavopunctata*	Peru	PA, 2 km S Cerro de Pasco	GQ357223
CP07-87	*Punapedaliodes*	*flavopunctata*	Peru	Pasco	DQ338861
CP17-02	*Redonda*	*lossadana*	Venezuela	Mérida: Tuñame	GQ357243
CP17-01	*Steromapedaliodes*	*albonotata*	Venezuela	Mérida: El Batallón	GQ357244
BLU711	*Praepedaliodes*	*amussis*	Brazil	Santa Catarina: São Bento do Sul (Road to Joinville)	MF415568
BLU780	*Praepedaliodes*	*amussis*	Brazil	São Paulo: São José do Barreiro (Sertão da Bocaina)	MF415579
BLU781	*Praepedaliodes*	*amussis*	Brazil	São Paulo: São José do Barreiro (Sertão da Bocaina)	MF415580
YPH0446	*Praepedaliodes*	*amussis*	Brazil	São Paulo: Serra da Bocaina	MF415593
BLU550	*Praepedaliodes*	*duartei*	Brazil	Bahia: Camacan (Reserva Serra Bonita)	MF415552
BLU632	*Praepedaliodes*	*exul*	Brazil	Minas Gerais: Itamonte (Parque Nacional do Itatiaia)	MF415561
BLU709	*Praepedaliodes*	*exul*	Brazil	Rio de Janeiro: Itatiaia (Parque Naciona do Itatiaia, Morro do Couto)	MF415567
YPH0442	*Praepedaliodes*	*exul*	Brazil	Minas Gerais: Itamonte (Parque Nacional do Itatiaia)	MF415589
YPH0460	*Praepedaliodes*	*exul*	Brazil	Minas Gerais: Alto Caparaó (Parque Nacional do Caparaó)	MF415597
YPH0461	*Praepedaliodes*	*exul*	Brazil	Minas Gerais: Alto Caparaó (Parque Nacional do Caparaó)	MF415598
BLU369	*Praepedaliodes*	*francinii*	Brazil	Minas Gerais: Alto Caparaó (Parque Nacional do Caparaó)	MF415546
BLU371	*Praepedaliodes*	*francinii*	Brazil	Minas Gerais: Alto Caparaó (Parque Nacional do Caparaó)	MF415547
BLU623	*Praepedaliodes*	*francinii*	Brazil	São Paulo: Campos do Jordão (Parque Estadual de Campos do Jordão)	MF415554
BLU625	*Praepedaliodes*	*francinii*	Brazil	São Paulo: Campos do Jordão (Parque Estadual de Campos do Jordão)	MF415556
BLU626	*Praepedaliodes*	*francinii*	Brazil	São Paulo: Campos do Jordão (Alto da Boa Vista)	MF415557
BLU627	*Praepedaliodes*	*francinii*	Brazil	São Paulo: Campos do Jordão (Parque Estadual de Campos do Jordão)	MF415558
BLU717	*Praepedaliodes*	*francinii*	Brazil	Santa Catarina: Urubici (Serra do Corvo Branco)	MF415574
BLU719	*Praepedaliodes*	*francinii*	Brazil	Santa Catarina: Urubici (Serra do Corvo Branco)	MF415577
YPH0398	*Praepedaliodes*	*francinii*	Brazil	São Paulo: Campos do Jordão (Alto do Capivari)	MF415586
YPH0448	*Praepedaliodes*	*francinii*	Brazil	São Paulo: Serra da Bocaina	MF415595
BLU659	*Praepedaliodes*	*granulata*	Brazil	São Paulo: Santo André (Parque das Nascentes de Paranapiacaba)	MF415565
BLU713	*Praepedaliodes*	*granulata*	Brazil	Paraná: Morretes (Serra da Graciosa)	MF415570
BLU714	*Praepedaliodes*	*granulata*	Brazil	Paraná: Morretes (Serra da Graciosa)	MF415571
CP12-01	*Praepedaliodes*	*granulata*	Brazil	São Paulo: Paranapiacaba	DQ338857
BLU373	*Praepedaliodes*	*landryi*	Brazil	Minas Gerais: Alto Caparaó (Parque Nacional do Caparaó)	MF415549
BLU712	*Praepedaliodes*	*landryi*	Brazil	Paraná: Morretes (Serra da Graciosa)	MF415569
BLU720	*Praepedaliodes*	*landryi*	Brazil	Santa Catarina: Urubici (Serra do Corvo Branco)	MF415578
BLU721	*Praepedaliodes*	*landryi*	Brazil	Santa Catarina: São Bento do Sul (road to Joinville, Campo Alegre)	MF415566
BLU782	*Praepedaliodes*	*landryi*	Brazil	São Paulo: Santo André (Parque das Nascentes de Paranapiacaba)	MF415581
BLU784	*Praepedaliodes*	*landryi*	Brazil	São Paulo: Santo André (Parque das Nascentes de Paranapiacaba)	MF415582
YPH0369	*Praepedaliodes*	*landryi*	Brazil	São Paulo: Santo André (Parque das Nascentes de Paranapiacaba)	MF415584
YPH0443	*Praepedaliodes*	*landryi*	Brazil	São Paulo: Campos do Jordão (Alto da Boa Vista)	MF415590
YPH0445	*Praepedaliodes*	*landryi*	Brazil	São Paulo: Campos do Jordão (Alto da Boa Vista)	MF415592
YPH0447	*Praepedaliodes*	*landryi*	Brazil	São Paulo: Serra da Bocaina	MF415594
YPH0449	*Praepedaliodes*	*pawlaki*	Brazil	São Paulo: Serra da Bocaina	MF415596
BLU372	*Praepedaliodes*	*pawlaki*	Brazil	Minas Gerais: Alto Caparaó (Parque Nacional do Caparaó)	MF415548
BLU622	*Praepedaliodes*	*pawlaki*	Brazil	São Paulo: Pindamonhangaba (Pico do Itapeva)	MF415553
BLU624	*Praepedaliodes*	*pawlaki*	Brazil	São Paulo: Pindamonhangaba (Pico do Itapeva)	MF415555
YPH0397	*Praepedaliodes*	*pawlaki*	Brazil	São Paulo: Campos do Jordão (Alto do Capivari)	MF415585
YPH0444	*Praepedaliodes*	*pawlaki*	Brazil	São Paulo: Campos do Jordão (Pico do Itapeva)	MF415591
BLU290	*Praepedaliodes*	*phanias*	Brazil	Rio de Janeiro: Itatiaia (Parque Nacional do Itatiaia)	MF415544
BLU291	*Praepedaliodes*	*phanias*	Brazil	Rio de Janeiro: Itatiaia (Parque Nacional do Itatiaia)	MF415545
BLU405	*Praepedaliodes*	*phanias*	Brazil	São Paulo: Jundiaí (Serra do Japi)	MF415550
BLU406	*Praepedaliodes*	*phanias*	Brazil	São Paulo: Jundiaí (Serra do Japi)	MF415551
BLU779	*Praepedaliodes*	*phanias*	Brazil	São Paulo: São José do Barreiro (Sertão da Bocaina)	MF415607
CP10-04	*Praepedaliodes*	*phanias*	Brazil	São Paulo: Campinas (Mata de Santa Genebra)	DQ338592
NW127-15	*Praepedaliodes*	*phanias*	Brazil	Minas Gerais: Extrema (Serra do Lopo)	MF415605
YPH0353	*Praepedaliodes*	*phanias*	Brazil	São Paulo: Pindamonhangaba (Reserva Trabijú)	MF415583
YPH0405	*Praepedaliodes*	*phanias*	Brazil	RS: São Franciso de Paula (Floresta Nacional)	MF415587
BLU630	*Praepedaliodes*	*sequeirae*	Brazil	Minas Gerais: Itamonte (Parque Nacional do Itatiaia)	MF415559
BLU631	*Praepedaliodes*	*sequeirae*	Brazil	Minas Gerais: Itamonte (Parque Nacional do Itatiaia, Pedra do Camelo)	MF415560
BLU633	*Praepedaliodes*	*sequeirae*	Brazil	Minas Gerais: Itamonte (Parque Nacional do Itatiaia)	MF415562
BLU634	*Praepedaliodes*	*sequeirae*	Brazil	Minas Gerais: Itamonte (Parque Nacional do Itatiaia)	MF415563
BLU635	*Praepedaliodes*	*sequeirae*	Brazil	Minas Gerais: Itamonte (Parque Nacional do Itatiaia)	MF415564
BLU707	*Praepedaliodes*	*sequeirae*	Brazil	Rio de Janeiro: Itatiaia (Parque Naciona do Itatiaia, Morro do Couto)	MF415604
BLU708	*Praepedaliodes*	*sequeirae*	Brazil	Rio de Janeiro: Itatiaia (Parque Naciona do Itatiaia, Morro do Couto)	MF415566
BLU802	*Praepedaliodes*	*sequeirae*	Brazil	Minas Gerais: Itamonte (Parque Nacional do Itatiaia)	MF415606
YPH0441	*Praepedaliodes*	*sequeirae*	Brazil	Minas Gerais: Itamonte (Parque Nacional do Itatiaia)	MF415588
BLU715	*Praepedaliodes*	*zaccae*	Brazil	Santa Catarina: Urupema	MF415572
BLU716	*Praepedaliodes*	*zaccae*	Brazil	Santa Catarina: Urupema	MF415573
BLU718	*Praepedaliodes*	*zaccae*	Brazil	Santa Catarina: Urubici (Serra do Corvo Branco)	MF415575

The final matrix comprised 73 individuals of the ten species of *Praepedaliodes*, and the mentioned outgroups. Bayesian analyses (BI) were carried out using the program MrBayes 3.2 (Ronquist *et al* 2012) on the CIPRES portal (Miller *et al* 2010). The model-jumping feature of the program was utilized, thereby sampling all possible GTR submodels according to their posterior probability (Ronquist *et al* 2012). The gamma parameter was also included to allow site rate variation. Four simultaneous chains were run for 10 × 10^6^ generations for two runs, sampling trees every 1000 cycles. The first 2500 trees were discarded as “burn in” based on when the runs had converged and reached equilibrium. The convergence of the likelihood traces of the independent runs was assessed with TRACER v1.5, and the ESS (effective sample size) values were verified to be above 300 for all parameters, which indicates that they were sufficiently sampled to estimate their posterior distributions (Drummond *et al* 2006).

Pairwise genetic distances between individuals (based on barcodes only) (Table 2) were calculated using MEGA v. 6.0 (Tamura *et al* 2013), under Kimura two-parameter (K2P) model of nucleotide substitution (Kimura 1980), and the frequency distribution of genetic divergence was plotted using pairwise values.

## Study Area

The study area extends over the entire territory covered by the Atlantic Forest (*Mata Atlântica*), a terrestrial biome and region which extends along the Atlantic coast of Brazil from state in the north to Rio Grande do Sul state, and northern Uruguay in the south, and inland as far as Paraguay and Misiones Province in Argentina. It is a very varied biome and comprises several ecoregions, including tropical and subtropical moist broadleaf forests, tropical and subtropical grasslands, savannas, and shrublands, sand dunes and mangrove forests (Morellato & Haddad 2000). This study is concerned exclusively with three of these ecoregions: tropical moist forests that receive more than 2000 mm of rain a year including Lowland Tropical Moist Forests, Submontane Tropical Moist Forest, and Montane Tropical Moist Forest extending at higher altitude, above 800–1200 m across mountains and plateaus of southern Brazil. The Atlantic forest is a region of high endemism for plants and animals, but over 85% of the original area has been deforested, threatening many plants and animals with extinction (Ribeiro *et al* 2009).

## Results

### Systematic overview

***Praepedaliodes*** **Forster, 1964**

*Praepedaliodes* Forster, 1964: 152; Miller, 1968: 116; Adams, 1986: 276; Viloria & Pyrcz, 1994: 347; Lamas *et al*, 2004: 214; Pyrcz, 2010: 215, 242. Type species: *Pronophila phanias* Hewitson, 1862, by original designation

### Generic diagnosis

General appearance, including wing shape, venation pattern and head morphology similar to *Pedaliodes* and other genera of the *Pedaliodes* complex. Androconial patch covering median one-fourth to one-third FWD wing surface larger than in other genera of the *Pedaliodes* complex, except *Panyapedaliodes* Forster, 1964 and *Praepronophila* Forster, 1964 *.* Male genitalia with characteristic, ventrally constricted uncus near base (marginally in *P. amussis* and *P. sequeirae* Pyrcz, Dias & Dolibaina n. sp.), stout subunci, in some species with a wide base, extending to 2/3 of uncus length, more massive than in *Pedaliodes* and in other genera of this complex; slender valva with more than one, and generally with several dorsal sharp processes or serrations, not found in any other genus of the *Pedaliodes* complex (except in *P. amussis* whose dorsum is smooth, and *P. sequeirae* n. sp., which has a single massive dorsal process); aedeagus very long, thin and straight or slightly curved, similar to *Panyapedaliodes* which is however strongly curved in basal half, and differing noticeably from the contorted aedeagus of *Pedaliodes* and much shorter and/or thicker of other genera of the complex, or from the extremely slender and even longer aedeagus of *Physcopedaliodes* Forster, 1964. Female genitalia proximal unit characterized by two, prominent lateral pocket-like folds with a rippled surface, not apparent in other genera of the *Pedaliodes* complex, compressed towards the entrance of ductus bursae; simple, slat-like lamella antevaginalis with smooth edges, enclosing from above the entrance to ductus bursae; strongly sclerotized ductus bursae between half and two-thirds length of corpus bursae, similar to *Corderopedaliodes* Forster, 1964 and *Physcopedaliodes*, but differing from the less sclerotized and generally shorter ductus bursae of *Pedaliodes* and other genera of the complex; entrance of bursa with a sclerotized bulbous structure, not apparent in other genera of the *Pedaliodes* complex; bursa copulatrix with two wide signa extending over half to two-thirds of its length. Sexual dimorphism slight, females generally larger with more contrasting colour patterns on wing undersides.

***Praepedaliodes phanias*** (Hewitson, 1862)

(Fig 4a–f, Fig 8c–f, 11c–h, 14a–p, 16a and 19)

*Pronophila phanias* Hewitson, 1862: 6, pl. 3, fig 18

*Pedaliodes phanias*; Butler, 1867: 267

**Fig 1:**
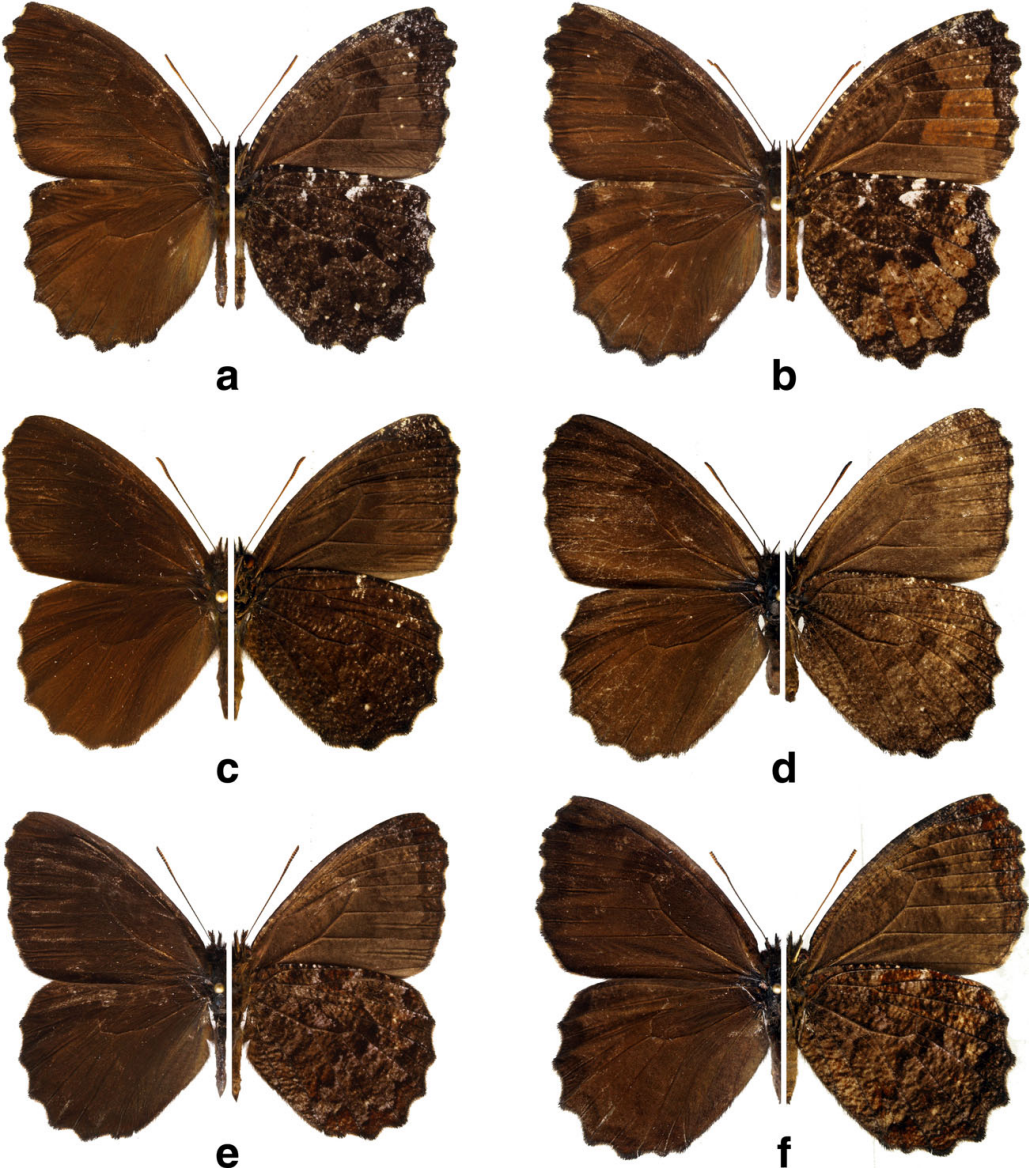
*Praepedaliodes* adults (left: dorsum; right: venter): **a** *Praepedaliodes amussis* (Thieme, 1905) male (P.N. da Serra da Bocaina, São José do Barreiro, São Paulo); **b** *Praepedaliodes amussis* female (P.N. da Serra da Bocaina, São José do Barreiro, São Paulo), **c** *Praepedaliodes francinii* n. sp. male paratype; **d** *Praepedaliodes francinii* n. sp. female paratype (Mundo Novo, Urubici, Santa Catarina); **e** *Praepedaliodes landryi* n. sp. male paratype (Serra do Corvo Branco, Urubici, Santa Catarina); **f** *Praepedaliodes landryi* n. sp. female paratype (Serra Dona Francisca, Joinville, Santa Catarina).

**Fig 2:**
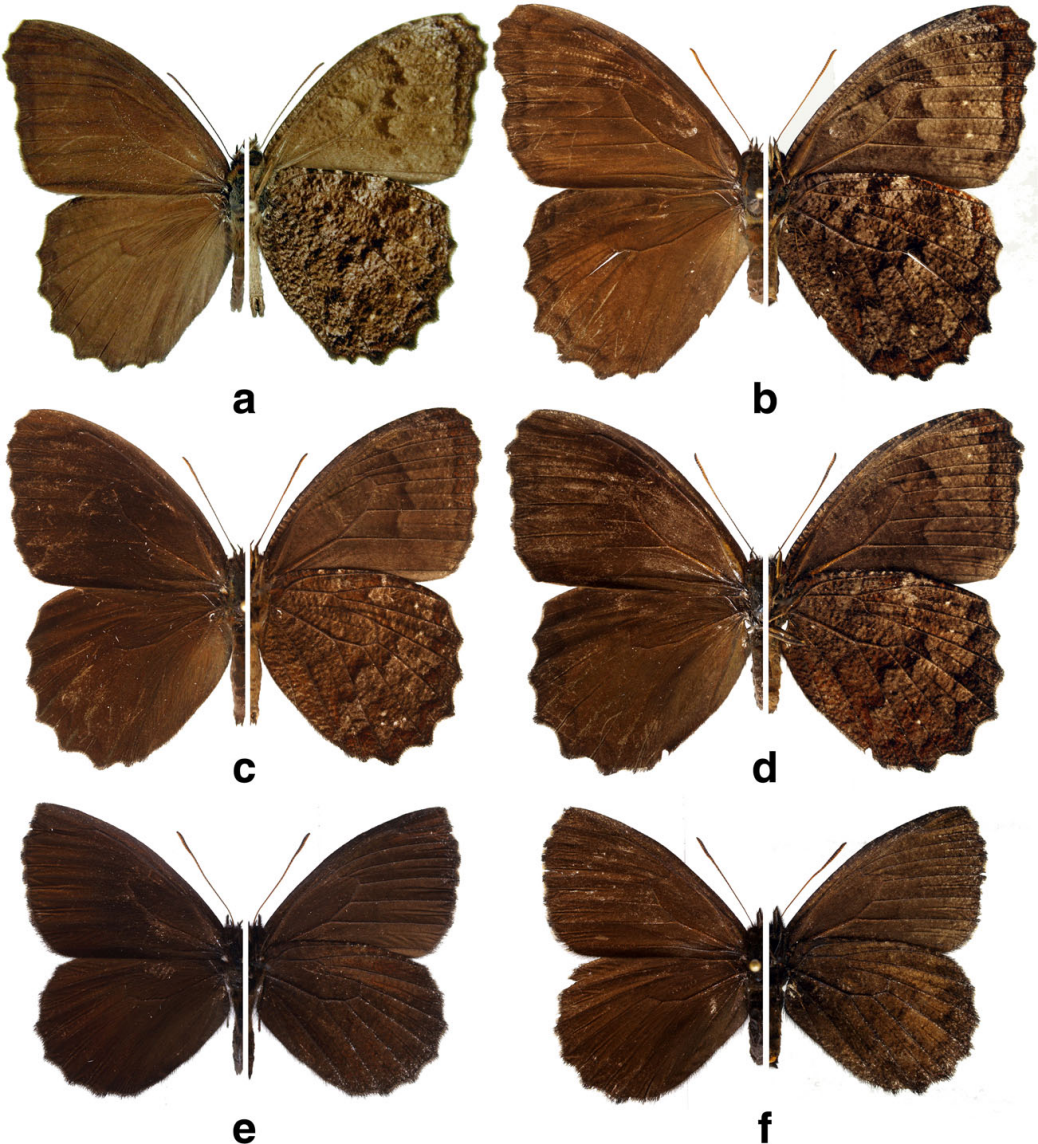
*Praepedaliodes* adults (left: dorsum; right: venter): **a** *Praepedaliodes duartei* n. sp. male paratype (Joinville, Santa Catarina); **b** *Praepedaliodes duartei* n. sp. female paratype (Morro do Araçatuba, Tijucas do Sul, Paraná); **c** *Praepedaliodes granulata* (Butler, 1868) male (Paraná, Morretes, Serra da Graciosa-Rio Taquari); **d** *Praepedaliodes granulata* female (Paraná, Morretes, Serra da Graciosa – Rio Taquari); **e** *Praepedaliodes sequeirae* n. sp. male paratype (P. N. do Itatiaia, km 13 da estrada para Agulhas Negras, Itatiaia, São Paulo); **f** *Praepedaliodes sequeirae* n. sp. female paratype (P. N. do Itatiaia, km 13 da estrada para Agulhas Negras, Itatiaia, São Paulo).

**Fig 3:**
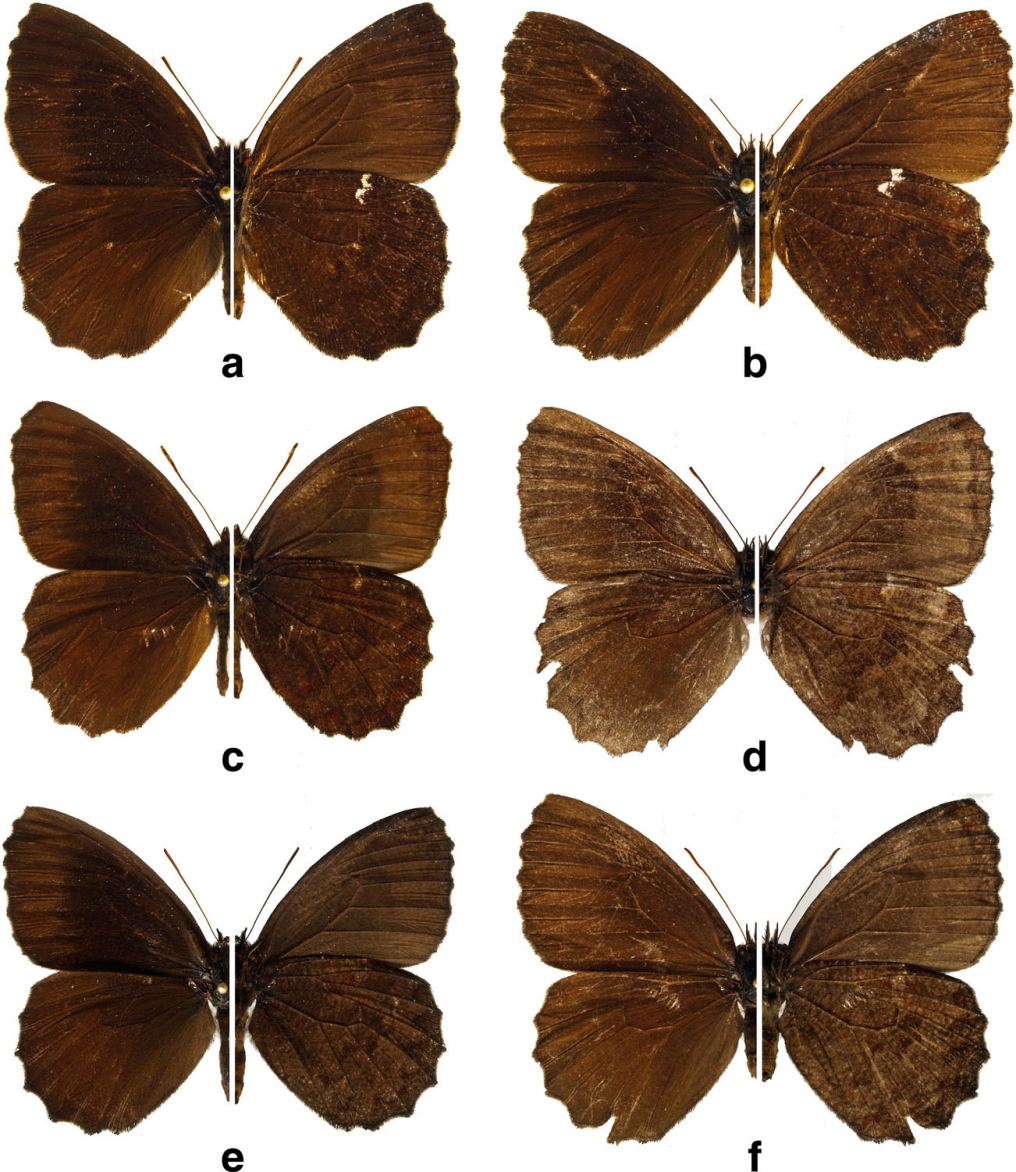
*Praepedaliodes* adults (left: dorsum; right: venter): **a** *Praepedaliodes exul* (Thieme, 1905) male (P.N. do Itatiaia, Itatiaia, São Paulo); **b** *Praepedaliodes exul* female (P.N. do Itatiaia, Itatiaia, São Paulo); **c** *Praepedaliodes pawlaki* n. sp. male paratype (Pico Itapeva, Pindamonhangaba, São Paulo); **d** *Praepedaliodes pawlaki* n. sp. female paratype (Pico Itapeva, Pindamonhangaba, São Paulo); **e** *Praepedaliodes zaccae* n. sp. male paratype (Morro das Antenas, Urupema, Santa Catarina); **f** *Praepedaliodes zaccae* n. sp. female paratype (Morro das Antenas, Urupema, Santa Catarina).

*Praepedaliodes phanias*; Forster, 1964: 152, figs 183, 184; D’Abrera, 1988: 852, 853, figs [1–2]; Brown & Freitas 2000: 103; Lamas *et al* 2004: 214; Peña *et al* 2006: 37, 39 (Fig 1), 40 (Fig 2), 41 (Fig 3) 43 (Fig 5), 45 (Fig 7); Brown *et al* 2007: 472; Pyrcz, 2010: 242; Francini *et al* 2011: 65

Type locality: Minas Gerais [Brazil]

Type material: *Pronophila phanias* Butler, 1867 was described based on an unstated number of specimens from Hewitson’s collection. One male syntype from this collection with the following labels, currently deposited at the NHMUK, is here designated lectotype to confirm the identity of the species: /B.M. TYPE No. Rh. 3969 *Pronophila phanias*, ♂ Hew. / Minas Gerais Hewitson Coll. 79–69 *Pronophila phanias* Hew. / Type /. It is illustrated by Warren *et al* (2016). Characteristic lectotype labels will be added to the specimen.

### Material examined

**Fig 4:**
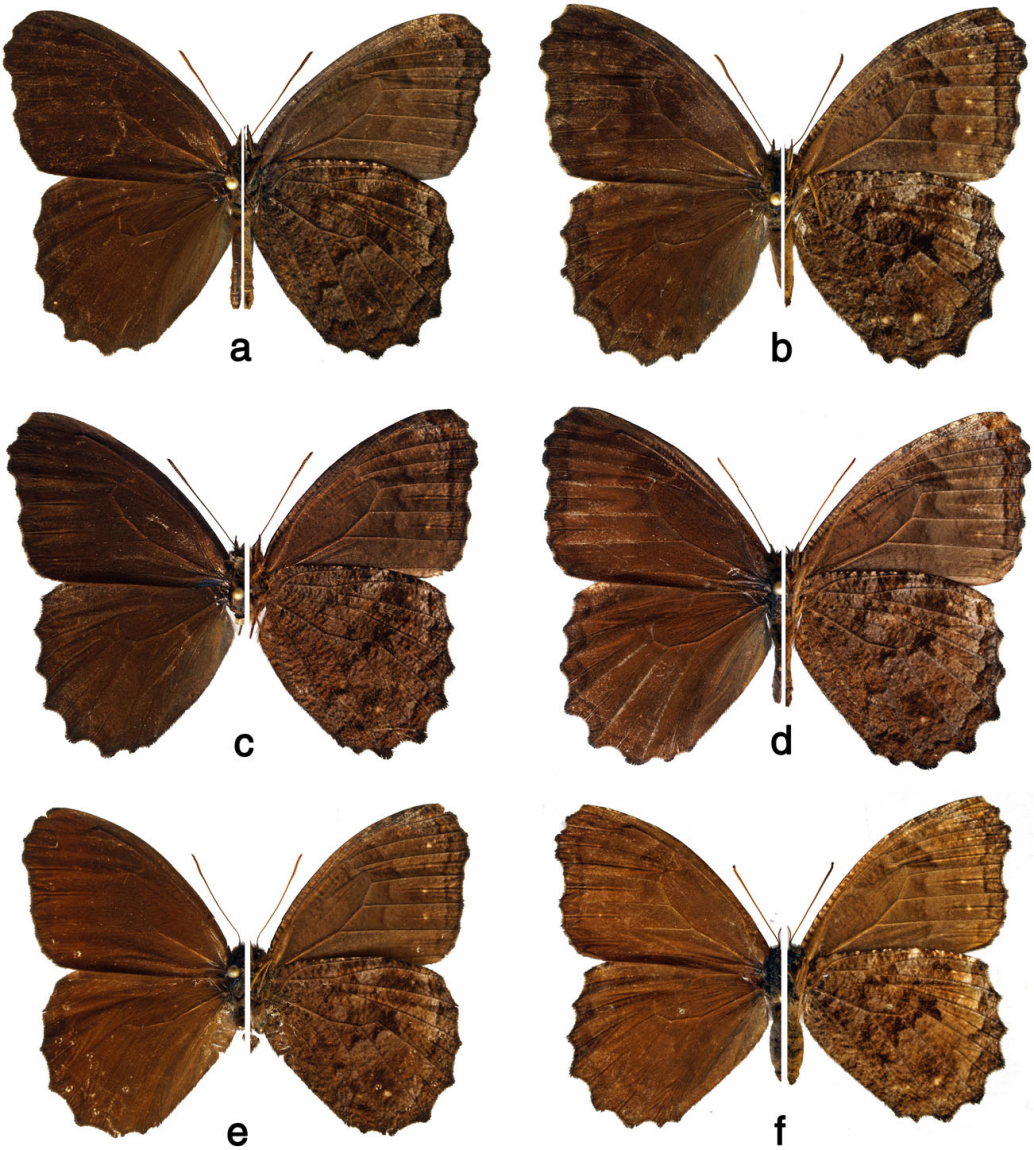
*Praepedaliodes* adults (left: dorsum; right: venter): **a** *Praepedaliodes phanias* (Hewitson, 1862) male (Serra da Graciosa, Morretes, Paraná); **b** *Praepedaliodes phanias* female (Britador, Turvo, Paraná); **c** *Praepedaliodes phanias* male (Serra do Lopo, Extrema, Minas Gerais); **d** *Praepedaliodes phanias* female (P.N. da Serra da Bocaina, São José do Barreiro, São Paulo); **e** *Praepedaliodes phanias* male (Puerto Iguazú, Misiones); **f** *Praepedaliodes phanias* female (Brazil).

BRAZIL: 1 ♂ 08.I.1941, ex coll. A. Jasiński (prep. genit. 02/09.05.2005 T. Pyrcz) (MZUJ), 1 ♂, ex coll. A. Jasiński (prep. genit. 01/25.06.2012 J. Lorenc) (MZUJ), 13 ♂ and 6 ♀, ex coll. Staudinger & Bang-Haas (MZUJ) [♀ Fig 4f]. *Minas Gerais*: **Alto Caparaó** (Parque Nacional do Caparaó, 20°25′2″S 41°50′9″W), 1250 m, 1 ♂ 08.II.2014, P. Boyer *leg.* (MZUJ). **Barbacena** (Serra da Mantiqueira), 900–1100 m, 1 ♂ 23.VIII.1951, H. Ebert *leg.*, ex coll. H. Ebert (DZ 36.987), 2 ♂ 22.VIII.1952, H. Ebert *leg.*, ex coll. H. Ebert (DZ 36.966, DZ 37.027), 1 ♂ 16.XI.1952, H. Ebert *leg.*, ex coll. H. Ebert (DZ 36.836) (DZUP). **Camanducaia** (Monte Verde), 1650 m, 1 ♂ 06.IX.1967, H. Ebert *leg.*, ex coll. H. Ebert (DZ 36.766) (DZUP). **Catas Altas** (Caraça), 1300–1500 m, 1 ♂ 01-05.IX.1985, Mielke & Casagrande *leg.* (DZ 36.957) (DZUP). **Conceição da Aparecida**, 1 ♂ 30.I.1960, Mielke *leg.* (OM 3.155), 1 ♀ 04.II.1960, Mielke *leg.* (OM 3.156) (OM). **Conceição dos Ouros**, 1 ♂ 25.VI.1967, ex coll. A. Cardoso (DZ 23.404 prep. genit. D. Dolibaina 2010) (DZUP). **Extrema** (Serra do Lopo), 1550–1650 m, 1 ♂ 21.II.2015, T. Pyrcz *leg.* (MZUJ) [♂ Fig 4c]. **Poços de Caldas**, 1250 m, 1 ♂ 02.XI.1966, H. Ebert *leg.*, ex coll. H. Ebert (DZ 36.656), 1 ♂ 10.XII.1966, H. Ebert *leg.*, ex coll. H. Ebert (DZ 36.955), 2 ♂ 23.IV.1967, H. Ebert *leg.*, ex coll. H. Ebert (DZ 36.816, DZ 36.867), 1 ♂ 14.XII.1966, H. Ebert *leg.*, ex coll. H. Ebert (DZ 36.666), 1 ♂ 27.V.1969, H. Ebert *leg.*, ex coll. H. Ebert (DZ 23.534 prep. genit. F.M.S. Dias 2012) (DZUP). *Espírito Santo*: **Santa Teresa**, 750 m, 1 ♀ 26.II.1970, H. Ebert, ex coll. H. Ebert (DZ 36.526), 1 ♀ 25.II.1972, H. Ebert *leg.*, ex coll. H. Ebert (DZ 36.776) (DZUP). *Rio de Janeiro*: **Itatiaia** (Parque Nacional do Itatiaia, South face), 1000 m, 1 ♂ 03.VII.1963, Mielke *leg.* (OM 5.221), 1 ♀ 13.VII.1968, Mielke *leg.* (OM 1.062) (OM), 1800 m, 1 ♀ 24.III.1964, H. Ebert *leg.*, ex coll. H. Ebert (DZ 36.995), 1000–1200 m, 1 ♂ 27.II.1964, H. Ebert *leg.*, ex coll. H. Ebert (DZ 36.967), 1 ♂ and 1 ♀ 07.IX.1964, H. Ebert *leg.*, ex coll. H. Ebert (1 ♂ DZ 23.524 gen. prep. F.M.S. Dias 2012) (DZ 23.524, DZ 36.977), 1100 m, 1 ♂ 03.VIII.1967, H. Ebert *leg.*, ex coll. H. Ebert (DZ 37.017), 1 ♀ 02.XI.1968, H. Ebert *leg.*, ex coll. H. Ebert (DZ 37.037), 1400 m, 1 ♀ 15.I.1969, H. Ebert *leg.*, ex coll. H. Ebert (DZ 36.887), 1 ♂ 22.XII.1957, H. Ebert *leg.*, ex coll. H. Ebert (DZ 27.470 prep. genit. F.M.S. Dias 2012) (DZUP), (Maromba Bridge) 1100 m, 2 ♂ 11-14.II.2011 (DNA vouchers BLU 290, BLU 291), 1 ♂ same data, A. V. L. Freitas *leg.* (ZUEC LEP 9945, ZUEC LEP 9946, ZUEC LEP 9947) (ZUEC). **Petrópolis** (Independência), 900 m, 1 ♀, ex coll. P. Gagarin (DZ 36.697), 1 ♂ 14.VIII.1936, P. Gagarin *leg.*, ex coll. H. Ebert (DZ 23.494 prep. genit. F. Dias 2012), 1 ♀ 20.VIII.1936, H. Ebert *leg.*, ex coll. H. Ebert (DZ 37.007 prep. genit. F. Dias 2016), 1 ♂ 28.II.1937, P. Gagarin *leg.*, ex coll. H. Ebert (DZ 36.937), (Taquara), 1 ♀ 11.VI.1972, ex coll. H. Ebert (DZ 36.927) (DZUP). **Nova Friburgo**, 1 ♂, 07.X.1982, A. Moser *leg.*, (CLAM). *São Paulo*: 1 ♂, ex coll. Staudinger & Bang-Haas (MZUJ). **Amparo**, 650 m, 1 ♂ 23.IV.1966, H. Ebert *leg.*, ex coll. H. Ebert (DZ 36.887). **Araras** 1 ♂ 31.V.1969, K. S. Brown *leg.* (ZUEC LEP 9942) (ZUEC). **Campos do Jordão** (Umuarama), 1800 m, 1 ♂ 08-15-III-1937, P. Gagarin *leg.*, ex coll. H. Ebert (DZ 36.997) (DZUP), (S22°46′1 W45°36′8), 1500 m, 1 ♂ 20.II.2014, P. Boyer *leg.* (MZUJ). **Campinas** (Mata da Santa Genebra), 650 m, 1 ♂ 06.II.2000, B-674, A. V. L. Freitas *leg.*, 1 ♂ (ex larva) IV.2002 (DNA voucher CP 10-04), A. V. L. Freitas *leg.* (ZUEC-AVLF). **Cordeirópolis**, 600 m, 1 ♀ 12.VIII.1962, H. Ebert *leg.*, ex coll. H. Ebert (DZ 36.907), 2 ♂ and 1 ♀ 25.XI.1962, H. Ebert *leg.*, ex coll. H. Ebert (DZ 23.514 prep. genit. F. Dias 2012, DZ 23.514, DZ 36.616, DZ 36.527), 1 ♂ 04.III.1963, H. Ebert *leg.*, ex coll. H. Ebert (DZ 36.927), 1 ♂ 22.IV.1966, H. Ebert *leg.*, ex coll. H. Ebert (DZ 36.657) (DZUP). **Cunha** (Serra da Bocaina, Bairro do Barro), 1150–1200 m, 1 ♀ 19.II.2014, T. Pyrcz *leg.* (MZUJ), (SP 171, km65), 1200 m, 1 ♂ 19.II.2014, P. Boyer *leg.* (MZUJ). **Mongaguá** (Poço das Antas), 200 m, 1 ♀ 17.III.1989, B-103, A. V. L. Freitas *leg.* (ZUEC-AVLF). **Jundiaí** (Serra do Japi), 900–1000 m, 2 ♂ and 1 ♀ 9.IX.2012, (DNA vouchers BLU570, BLU571, BLU572), Junia Y. O. Carreira *leg.* (ZUEC LEP 9939, ZUEC LEP 9940, ZUEC LEP 9941) (ZUEC). **Pindamonhangaba** (Trabijú) 1 ♂ 17.VIII.2013 (DNA voucher YPH 0353), A. H. B. Rosa *leg.* (ZUEC LEP 9943) (ZUEC). **Piquete**, 1 ♂, D’Almeida *leg.*, ex coll. D’Almeida (DZ 36.587), (Barreira do Piquete), 1400–1600 m, 1 ♂ 15.II.1984, Mielke & Casagrande *leg.* (DZ 36.857) (DZUP), (Serra da Mantiqueira), 1300–1350 m, 1 ♀ 23.IV.2005, T. Pyrcz *leg.* (MZUJ), 1450–1500 m, 1 ♂ 23.V.2005, T. Pyrcz *leg.* (prep. genit. 08/ T. Pyrcz) (MZUJ). **São José do Barreiro** (Parque Nacional da Serra da Bocaina, Trilha Principal), 1450–1500 m, 1 ♀ 13.II.2014, T. Pyrcz *leg.* (MZUJ), (Bocaina) [♀ Fig 4d], 1 ♀ 13.X.2015, (DNA voucher BLU 779), R. Raby *leg.* (ZUEC-AVLF). **São Paulo** (Serra da Cantareira), 900–1000 m, 1 ♀ XI.1942, H. Ebert *leg.*, ex coll. H. Ebert (DZ 36.737) (DZUP). **Serra Negra**, 1 ♂ and 1 ♀ 12.IX.1947, D’Almeida *leg.*, ex coll. D’Almeida (DZ 36.557, DZ 36.947) (DZUP), 2 ♂ 27.IX.1957 (OM 1.668, OM 1.669) (OM). *Paraná*: **Campo Bonito** (RPPN Hermínio e Maria), 1 ♂ 09.X.2012, Expedição LABLEP *leg.* (DZ 36.746) (DZUP). **Campo Mourão** (Parque Estadual do Lago Azul), 500–600 m, 1 ♂ and 1 ♀ 09-11.X.2010, Mielke, Dolibaina, Carneiro & Maia *leg.* (DZ 23.805, DZ 36.627) (DZUP). **Cianorte**, 600 m, 1 ♂ and 1 ♀ 09.XII.1975, Moure, Mielke & Wedderhoff *leg.* (DZ 36.547, DZ 36.827), 1 ♂ 11.XII.1975, Moure, Mielke & Wedderhoff *leg.* (DZ 36.607) (DZUP). **Curitiba**, 900 m, 1 ♂ 23.I.1971, H. Ebert *leg.*, ex coll. H. Ebert (DZ 37.038), 1 ♀ 02.IV.1973, Mielke *leg.* (DZ 36.826), 1 ♂ 21.XI.1977, Mielke *leg.* (DZ 36.717) (DZUP). **Fênix** (Parque Estadual de Vila Rica do Espírito Santo), 1 ♂ and 1 ♀ 03-04.X.1987, Mielke & Casagrande *leg.* (DZ 36.978, DZ 36.838) (DZUP). **Foz do Iguaçu**, 1 ♂ III.1952, ex coll. F. Justus (DZ 36.797), 2 ♂ XI.1969, Cardoso *leg.* (DZ 36.598, DZ 36.988), 1 ♂ 12.II.1978, Mielke & Miers *leg.* (DZ 36.828), (Parque Nacional do Iguaçu), 4 ♂ and 1 ♀ 03.XII.1966, Expedição Departamento de Zoologia-UFPR (DZ 36.888, DZ 36.878, DZ 36.707, DZ 5.596, DZ 36.546 prep. genit. O. Mielke), 1 ♂ 04.XII.1966, Expedição Departamento de Zoologia-UFPR (DZ 36.668), 3 ♂ and 1 ♀ 05.XII.1966, Expedição Departamento de Zoologia-UFPR (DZ 36.868, DZ 36.928, DZ 36.798, DZ 36.637), 2 ♂ 07.XII.1966, Expedição Departamento de Zoologia-UFPR (DZ 36.898, DZ 36.908), 1 ♂ 10.XII.1966, Expedição Departamento de Zoologia-UFPR (DZ 36.958), 2 ♂ 06.IX.1985, Mielke & Casagrande (DZ 27.509, DZ 36.938 prep. genit. F. Dias 2012), 250 m, 10 ♂ and 8 ♀ 17.II.1969, Moure & Mielke *leg.* (DZ 36.558, DZ 36.548, DZ 36.698, DZ 36.518, DZ 36.628, DZ 36.818, DZ 36.788, DZ 36.817, DZ 36.747, DZ 36.667, DZ 36.528, DZ 36.738, DZ 36.648, DZ 36.748, DZ 36.718, DZ 37.018, DZ 36.807, DZ 37.028), 2 ♂ and 2 ♀ 21-24.IV.1995, Mielke & Casagrande *leg.* (DZ 36.596, DZ 37.016, DZ 36.906, DZ 37.036) (DZUP), 1 ♀ 09.V.2000, T. Pyrcz *leg.* (prep. genit. 02/25.06.2012 J. Lorenc) (MZUJ). **Guarapuava** 1 ♀ III.1950, H. Schneider *leg.*, ex coll. F. Justus (DZ 36.577), 1000 m, 1 ♂ II.1978, H. Schneider *leg.*, ex coll. H. Ebert (DZ 36.507), 1100 m, 1 ♂ I.1983, H. Schneider *leg.*, ex coll. H. Ebert (DZ 36.597) (DZUP), (Salto São Francisco), 1000 m, 1 ♂ and 2 ♀ 01.V.2008, D. Dolibaina *leg.* (prep. genit. 01/10.06.2015 J. Lorenc) (MZUJ), (Guará), 1200–1250 m, 1 ♂ and 1♀ 04.V.2012, T. Pyrcz *leg.*, (prep. genit. 09/20.05.2012 J. Lorenc) (MZUJ). **Jussara** (Horto CMNP), 500 m, 1 ♂ 16.XI.1975, Mielke & Rosado *leg.* (DZ 36.687) (DZUP). **Londrina**, 1 ♂, Takemura *leg.*, ex coll. D’Almeida (DZ 36.897) (DZUP). **Manoel Ribas** (Rio Ivaí), 450–600 m, 1 ♂ 12.X.2010, Mielke, Dolibaina, Carneiro & Maia *leg.* (DZ 36.556) (DZUP). **Maringá**, 2 ♂ 28.I.1971, Becker *leg.* (DZ 36.617, DZ 36.847) (DZUP). **Moreira Salles** (RPPN Moreira Salles), 1 ♂ 07.X.2012, Expedição LABLEP *leg.* (DZ 36.956) (DZUP). **Morretes** (Serra da Graciosa BR 116, 50 Km NE Curitiba) (DZUP), 870 m, 1 ♂ and 1 ♀ 31.I.2015, P. Boyer *leg.* (MZUJ), 870–880 m [♂ Fig 4a], 3 ♂ and 1♀ 31.I.2015, T. Pyrcz *leg.* (prep. genit. 02/10.06.2015 J. Lorenc) (MZUJ), (Serra da Graciosa, Rio Taquari, S25°19′ W48°56′), 800–850 m [♀ Fig 4b], 1 ♀ 29.V.2012, T. Pyrcz *leg.* (MZUJ), (Serra da Graciosa, Rio Taquari, S25°19′ W48°56′), 850–900 m, 1 ♀ 01.II.2015, P. Boyer *leg.* (MZUJ). **Ponta Grossa** (Pedreira), 1 ♀ 04.VIII.1945, F. Justus *leg.*, ex coll. F. Justus (DZ 36.576) (DZUP). **Rolândia** (Rio Tibagi), 750 m, 1 ♀ II.1949, Walz *leg.*, ex coll. H. Ebert (DZ 36.545), 1 ♀ IV.1949, Walz *leg.*, ex coll. H. Ebert (DZ 36.806), 1 ♂ X.1949, Walz *leg.*, ex coll. H. Ebert (DZ 36.757), 1 ♂ 20.IV.1972, Walz *leg.*, ex coll. H. Ebert (DZ 36.727) (DZUP). **Roncador** (UC São Domingos), 1 ♂ 11.X.2010, Mielke, Dolibaina, Carneiro & Maia *leg.* (DZ 36.677) (DZUP). **São João do Ivaí** (Porto Ubá), 1 ♂ I.1950, ex coll. F. Justus (DZ 36.647) (DZUP). **São Jorge do Ivaí** (Copacabana), 550 m, 1 ♀ 16.IX.1970, Furtado *leg.* (DZ 36.517), 1 ♂ 15.XI.1975, Mielke & Rosado *leg.* (DZ 36.567) (DZUP). **São Pedro do Ivaí** (RPPN Barbacena), 300 m, 3 ♂ and 1 ♀ 08.X.2010, Mielke, Dolibaina, Carneiro & Maia *leg.* (DZ 36.767, DZ 36.777, DZ 36.837, DZ 37.006), 4 ♂ 02.X.2012, Expedição LABLEP (DZ 36.605, DZ 36.866, DZ 36.936, DZ 36.986), 1 ♂ 07.X.2012, Expedição LABLEP (DZ 36.855) (DZUP). **Terra Boa** (CMNP), 650 m, 1 ♂ 11.XII.1975, Moure, Mielke & Wedderhoff *leg.* (DZ 5.597 prep. genit. O. Mielke) (DZUP). **Tijucas do Sul** (Campo do Quiriri, 25 Km S of Tijucas do Sul S26°00′290 W49°02′578) 850 m, 1 ♀ 04.II.2015, P. Boyer *leg.* (MZUJ). **Toledo**, 1 ♂ VIII-IX.1964, Mohr *leg.* (OM 3.030) (OM). **Três Barras do Paraná** (Parque Estadual do Rio Guarani), 1 ♀ 09-11-X.2012, Expedição LABLEP (DZ 36.786). **Tuneiras do Oeste** (REBIO das Peróbas), 1 ♀ 06.X.2012, Espedição LABLEP (DZ 36.846) (DZUP). **Turvo** (Britador), 1000 m, 1 ♀ 21.XI.2007, Dolibaina *leg.* (MZUJ), 2 ♂ 24-30.XII.2010, Dolibaina *leg.* (DD 278, DD 286) (DD), (Fazenda Baitala, Rio Ivaí), 2 ♂ 21.IV.2007, Dolibaina *leg.* (DZ 23.374, DD 283 prep. genit. D. Dolibaina 2010) (DZUP), 1 ♂ 01.XI.2009, Dolibaina *leg.* (DD 281) (DD), (Rio Ivaí), 500 m, 2 ♂ 20.I.2008, Dolibaina *leg.* (MZUJ). *Santa Catarina*: **Guarujá do Sul**, 04.II.1992, A. Moser *leg.*, prep. genit. 02/24.05.2012 J. Lorenc-Brudecka (CLAM). **Santa Cecília** (Campo Alto), 1200 m, 1 ♀ 27.II.1968, Moure & Mielke *leg.* (DZ 36.808) (DZUP). **São Bento do Sul**, 1 ♀ 02.XII.1969, H. Ebert *leg.*, ex coll. H. Ebert (DZ 36.768), 1 ♂ 05.VIII.1971, H. Ebert *leg.*, ex coll. H. Ebert (DZ 36.638), 850 m, 1 ♂ 04.VIII.1975, H. Ebert *leg.*, ex coll. H. Ebert (DZ 37.008), 1 ♂ 14.III.1980, H. Ebert *leg.*, ex coll. H. Ebert (DZ 36.678) (DZUP). **Seara** (Nova Teotônia), 350 m, 1 ♂ 11.II.1973, H. Ebert *leg.*, ex coll. H. Ebert (DZ 36.568) (DZUP). **Urubici** (Vacas Gordas, Serra Geral Km 3 to Bom Jardim), 1450–1500 m, 1 ♀ 10.II.2015, T. Pyrcz *leg.* (MZUJ). **Urupema** (Morro das Antenas 27°55′58″S 49°51′33″W), 1550–1600 m, 1 ♂ 06.II.2015, P. Boyer *leg.* (MZUJ). *Rio Grande do Sul*: **Bom Jesus** (Bom Jesus – São Joaquim, Km 15–25), 800–1000 m, 1 ♀ 13.II.2015, T. Pyrcz *leg.* (MZUJ). **Catuípe** (28°15′21″S 54°00′28″W), 300 m, 1 ♂ 15.XII.2008, F. Santos *leg.* (DD 275), 1 ♂ IV.2009, F. Santos *leg.* (DD 279) (DD). **Derrubadas** (Parque Estadual do Turvo), 1 ♂ 10.XI.1985, Mielke, Araújo & Casagrande *leg.* (DZ 36.716) (DZUP), 1 ♀ 01-06.II.2009, 400 m, A. Moser leg. (UFRGS). **Guarani** (São Luiz Gonzaga), 1 ♂ 08.VII.1939, Pe. Piton *leg.*, ex coll. D’Almeida (DZ 36.636) (DZUP). **Ijuí**, 1 ♂ 25.IX.2003, F. Santos *leg.* (DD 285) (DD). **Pelotas**, 1 ♂ 01.VI.1961, J. L. Mantovani-Biezanko *leg.* (MZUJ), 1 ♂ 27.IX.1961, C. M. Biezanko *leg.* (MZUJ), 1 ♂ 07.V.1964, J. L. Mantovani-Biezanko *leg.*, ex coll. A. Jasiński (MZUJ), 1 ♂ 31.III.1964, ex coll. H. Ebert (DZ 36.646), 1 ♂ 07.III.1966, Biezanko *leg.*, ex coll. H. Ebert (DZ 36.706), 1 ♂ IV.1968, Guerra *leg.*, ex coll. H. Ebert (DZ 36.686) (DZUP). **Porto Mauá**, 4 ♂ 12.X.2008, Thiele *leg.* (DD 277, DD 282, DD 284, DD 287) (DD). **Santa Rosa** (Rio Uruguai), 1 ♂ 07.XI.1954, A. Langwiński *leg.*, ex coll. A. Jasiński (MZUJ), (Pedregulho), 1 ♀ 15.V.1955, A. Langwiński *leg.*, ex coll. A. Jasiński (MZUJ). **São Francisco de Paula** (Floresta Nacional de São Francisco de Paula), 1 ♂ 26.XII.2013 (DNA voucher YPH 0405), L. A. Kaminski *leg.* (ZUEC LEP 9944) (ZUEC), **Carlos Barbosa** (Arroio Santa Clara), 1 ♂ 23.IV.1996, 400 m, A. Moser *leg*. (CLAM). **Tucunduva**, 400 m, 1 ♂ 09.II.1976, Mielke & Buzzi *leg.* (DZ 36.946) (DZUP). **Novo Hamburgo** (Lomba Grande), 1 ♀ 06.IV.1991, A. Moser *leg.*, (Dois Irmaos), 1 ♂ 22.XII.1991, A. Moser *leg*., (Estancia Velha), 1 ♂ 13.XII.1991, A. Moser *leg.* (CLAM). PARAGUAY: *Alto Paraná*: **Itakyry** (General Días), 400 m, 1 ♂ 15-20.I.1980, O.-C. Mielke & Miers *leg.* (DZ 36.506) (DZUP). *Guairá*: **Independencia** (Villarica), 1 ♂ 07.XI.1951, Foester *leg.*, ex coll. H. Ebert (DZ 36.676), 1 ♂ 21.XII.1951, Foester *leg.*, ex coll. H. Ebert (DZ 36.996) (DZUP). ARGENTINA: *Misiones*: **Rio Uruguai**, 1 ♂ IX.1950, Foester *leg.* (DZ 36.896**)** (DZUP). **Bernardo de Irigoyen** (RN 14, km 17 to San Pedro), 700–750 m, 1 ♂10.XII.2000, P. Boyer *leg.* (PBF). **Campo Viera**, 1 ♂ and 1 ♀ 10.XI.1954, Foester *leg.*, ex coll. H. Ebert (DZ 36.916, DZ 36.726) (DZUP). **Garuhape** (RP 220, 26 km S), 550 m, 4 ♂ 07.XII.2000, P. Boyer *leg.* (PBF), 1 ♂ same data as above (prep. genit. 01/01.07.2008, T. Pyrcz) (MZUJ). **General Belgrano** (Almirante Brown, Reserva Yacutinga), 5 ♂ 02-05.III.2007, Mielke & Casagrande *leg.* (DZ 23.705, DZ 36.566, DZ 36.606, DZ 36.786, DZ 36.926) (DZUP). **Leandro N. Alem**, 1 ♂ XI.1950, Foester *leg.*, ex coll. H. Ebert (DZ 36.886), 1 ♂ XI.1959, Foester *leg.*, ex coll. H. Ebert (DZ 36.586) (DZUP). **Loreto**, 1 ♂ II.1955, Walt *leg.*, ex coll. H. Ebert (DZ 36.535) (DZUP). **Panambi** (RP5, km 8, 25–30 km from Obera), 350–400 m, 1 ♀ 06.XII.2000, P. Boyer *leg.* (PBF). **Posadas**, 1 ♂ XII.1950, Foester *leg.*, ex coll. H. Ebert (DZ 36.696) (DZUP). **Puerto Iguazú**, 1 ♂ 1955, Walt *leg.*, ex coll. H. Ebert (DZ 36.536) (DZUP), 200 m, 1 ♂ IX.1996, E. Nuńez-Bustos *leg.* (prep. genit. 03/01.02.2008 T. Pyrcz) (MZUJ) [Fig 4e]. **San José** (Arroyo Liso, RN 14, 20 km NE of San Jose), 200 m, 2 ♂ and 1 ♀ 05.XII.2000, P. Boyer *leg.* (PBF).

### Redescription

*Male* (Figs 4a, c, e, 16a). *Head*. Antennae reaching 2/5 the length of costa, slender, dorsally medium brown, ventrally orange, naked except for some sparse whitish scales in basal one-fourth, club of 12–13 flagellomeres, slightly thicker than shaft, ventrally light orange; eyes chocolate brown and blackish brown, covered with rather short and dense black setae; labial palpi one and a half the length of head, dorsally and laterally covered with chestnut scales and ventrally with sandy yellow scales, very short on third segment. *Thorax.* Dorsally and ventrally black, sparsely hairy; patagia made of grey brown scales; tegulae covered with brown and golden scales; legs grey brown, femur covered with grey and brown scales, tibiae and tarsi covered with sandy yellow scales. *Abdomen.* Dorsally covered with dark brown, ventrally and laterally with greyish brown scales. *Wings.* FW (length: 21–29 mm) with a blunt apex and slightly situate outer margin, produced below apex; fringes short, grey; FWD uniform, varying between chestnut, medium and dark brown (becoming lighter in older individuals), lustrous, with a faint darker submarginal line; androconial patch a shade darker than the ground colour, apparent only in lighter individuals, large and elongated, extending along veins from discal cell distal edge to anal margin, entering slightly discal cell. FWV variable, from chestnut to taupe brown, but generally a shade lighter and duller than on the dorsal surface; a slightly lighter postdiscal to submarginal band extending distally from an irregular postdiscal line, enclosing a row of minute, faint and in some individuals not apparent milky white dots; some lilac scales in apical and subapical areas; a darker, chocolate brown area extending from the submarginal dark brown line to outer margin. HW oval with a scalloped outer margin; fringes short, grey brown; HWD uniform but of variable brown, from medium to dark and taupe, lustrous, sparsely hairy in median half and along anal margin. HWV varying from chestnut to taupe brown with some irregular dark brown patches, postdiscal line forms a characteristic distal notch on vein M_3_, in some individuals produced distally; a minute yellowish submarginal dot in cell CuA_1_-CuA_2_; an irregular dark brown submarginal line, edged basally with some lighter, sandy yellow scales, not apparent in some individuals. Genitalia (Fig 8c–f): Tegumen massive, triangular in lateral view, dorsum flat, slightly produced ventrally between subuncus base and pedunculus; uncus the length of tegumen dorsum, slender, ventrally strongly constricted near base, and slightly arched downwards in the middle, with a sharp tip; subunci massive, wide at base and strongly adhered to tegumen, gradually narrowing towards a sharp tip, three-fourths the length of uncus; pedunculus (syn. appendix angular) short; vinculum slender; saccus deep, the length of uncus, two times as wide as vinculum (understood as a combination of the ventral arm of the tegumen and the dorsal arm of the saccus) in lateral view; valva slender, the length of tegumen + uncus, gradually narrowing towards apex, with a series of protrusions and teeth-like processes along distal half of dorsal surface, of variable number and shape, none of which being significantly more prominent than the others, except for the pointed, upstanding subapical process which is generally longer than the remaining ones; aedeagus slender, the length of saccus + valva, arched, with a sharp tip, smooth, spoon-like at base; proximal opening one-third the length of aedeagus.

**Fig 5:**
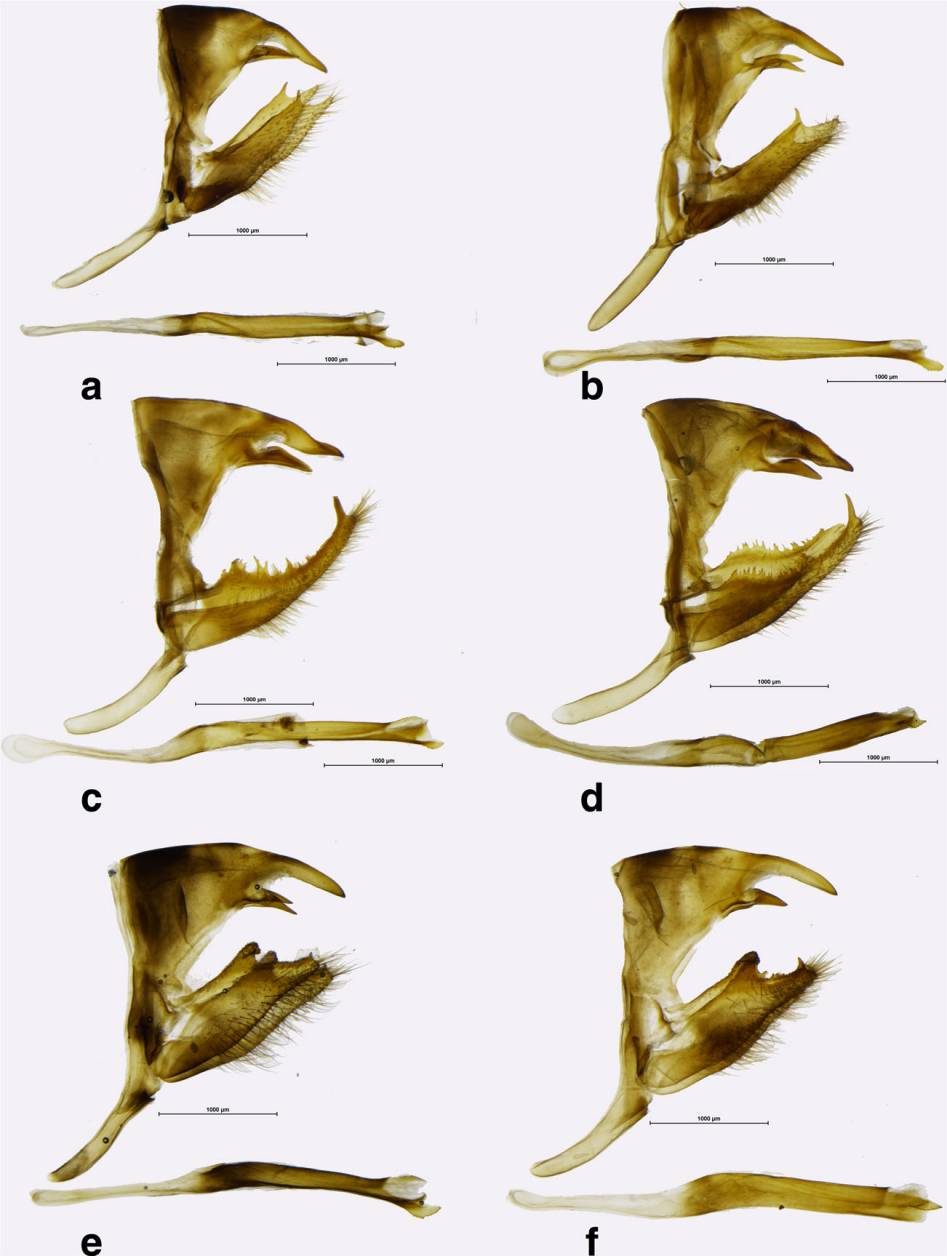
*Praepedaliodes* male genitalia (lateral view, aedeagus extracted from its natural position): **a** *Praepedaliodes amussis* (P.N. da Serra da Bocaina, São José do Barreiro, São Paulo); **b** *Praepedaliodes amussis* (above Piquete, Serrra da Mantiqueira, Piquete, São Paulo); **c** *Praepedaliodes granulata* (Morro do Araçatuba, Tijucas do Sul, Paraná); **d** *Praepedaliodes granulata* (Paraná, Morretes, Serra da Graciosa – Casa de Pedra); **e** *Praepedaliodes sequeirae* n. sp. (Itatiaia, Pico); **f** *Praepedaliodes sequeirae* n. sp. (P. N. do Itatiaia, km 13 da estrada para Agulhas Negras, Itatiaia, São Paulo).

**Fig 6:**
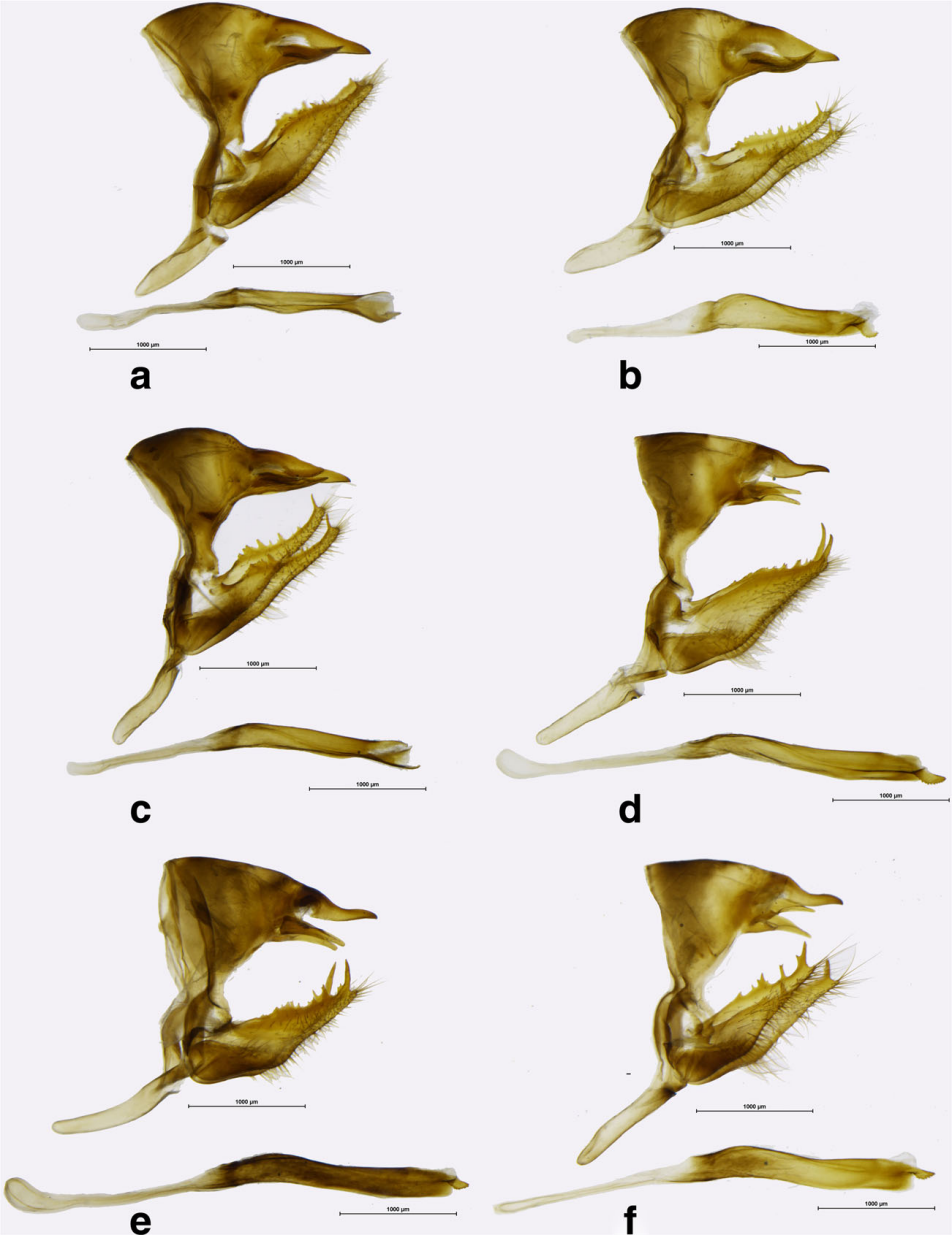
*Praepedaliodes* male genitalia (lateral view, aedeagus extracted from its natural position): **a** *Praepedaliodes landryi* n. sp. (Pico Itapeva, Pindamonhangaba, São Paulo); **b** *Praepedaliodes landryi* n. sp. (Paraná, Morretes, Serra da Graciosa – Casa de Pedra); **c** *Praepedaliodes landryi* n. sp. (Serra do Corvo Branco, Urubici, Santa Catarina); **d** *Praepedaliodes francinii* n. sp. (Mundo Novo, Urubici, Santa Catarina); **e** *Praepedaliodes francinii* n. sp. (Pico Itapeva, Pindamonhangaba, São Paulo); **f** *Praepedaliodes francinii* n. sp. (via parking – Serra do Caparaó, Alto Caparaó, Minas Gerais).

**Fig 7:**
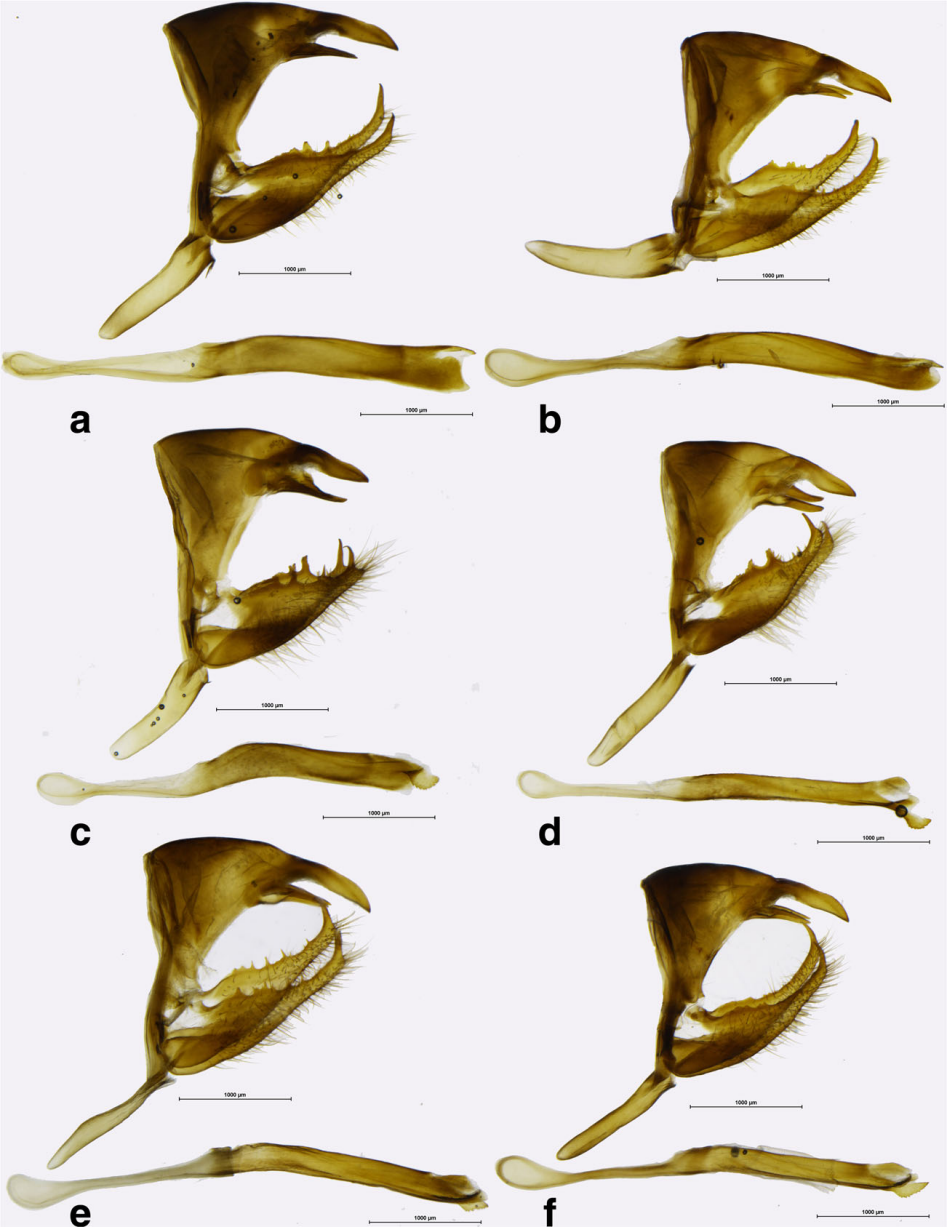
*Praepedaliodes* male genitalia (lateral view, aedeagus extracted from its natural position): **a** *Praepedaliodes exul* (P.N. do Itatiaia, Itatiaia, São Paulo Itatiaia); **b** *Praepedaliodes exul* (Serra do Caparaó, Alto Caparaó, Minas Gerais); **c** *Praepedaliodes pawlaki* n. sp. (La Antena – P.N. da Serra da Bocaina, São José do Barreiro, São Paulo); **d** *Praepedaliodes pawlaki* n. sp. (Pico Itapeva, Pindamonhangaba, São Paulo); **e** *Praepedaliodes zaccae* n. sp. (Paraná, Morretes, Serra da Graciosa – Casa de Pedra); **f** *Praepedaliodes zaccae* n. sp. (Morro das Antenas, Urupema, Santa Catarina).

**Fig 8:**
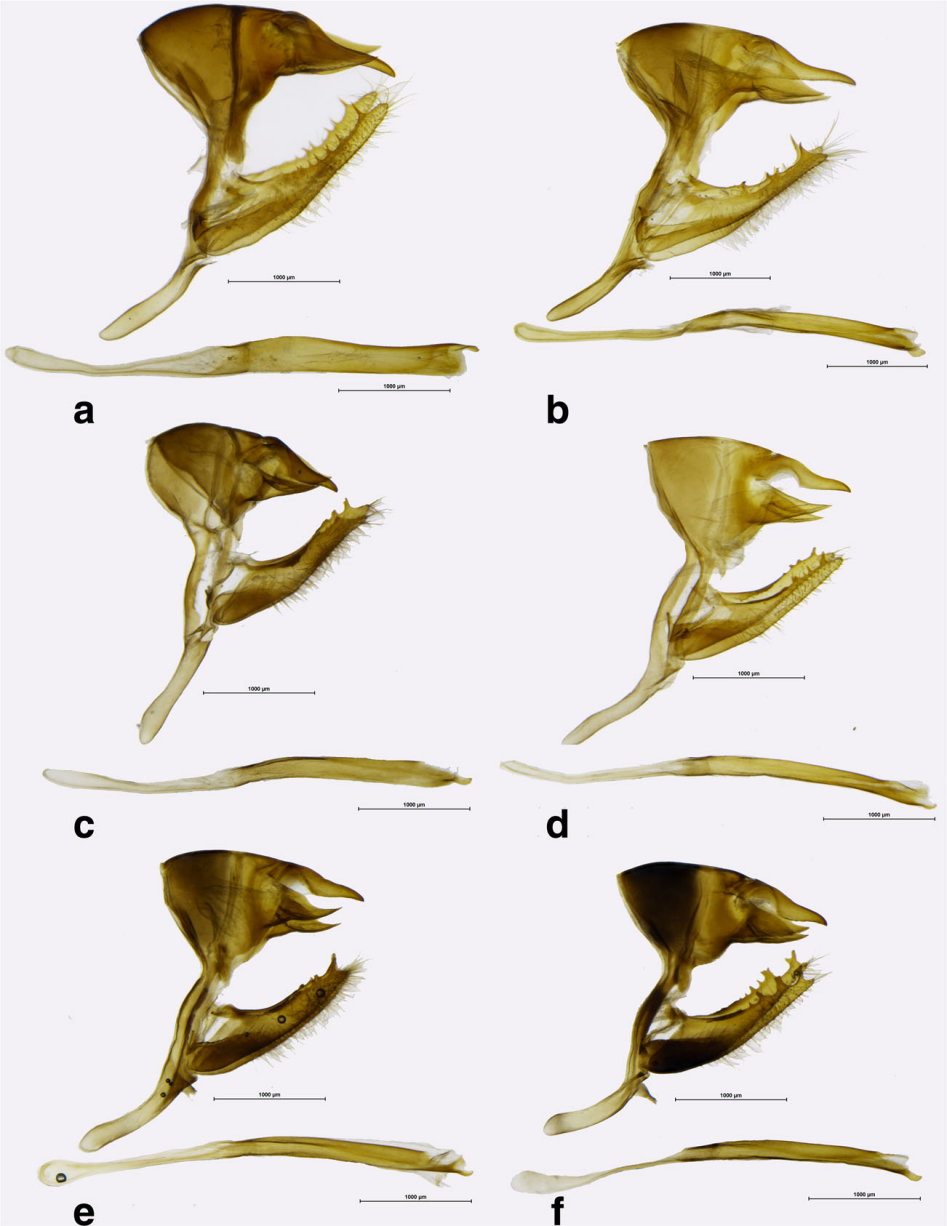
*Praepedaliodes* male genitalia (lateral view, aedeagus extracted from its natural position): **a** *Praepedaliodes duartei* n. sp. (Joinville, Santa Catarina); **b** *Praepedaliodes duartei* n. sp. (Joinville, Santa Catarina); **c** *Praepedaliodes phanias* (Garuhape, Misiones); **d** *Praepedaliodes phanias* (Guaruja do Sul, Santa Catarina); **e** *Praepedaliodes phanias* (Serra da Graciosa – Rio Taquari, Morretes, Paraná); **f** *Praepedaliodes phanias* (Extrema, Serra do Lopo, Minas Gerais).

*Female* (Figs 4 b, d, f, 11c–h). Lighter on both the upper and underside, especially with a much more contrasting and better patterned FWV and HWV with noticeable submarginal yellow dots and much better defined postdiscal and submarginal lines. Genitalia (Figs 11c–h). Genitalia strongly compressed laterally in ventral view; papilla analis prominent, gently rounded in lateral view, covered with short, and delicate hair; proximal unit, in lateral view, consisting of a well-sclerotized slat-like, smooth lamella postvaginalis and two, rather weakly sclerotized prominent lateral pocket-like folds with a delicately rippled surface, compressed towards the entrance of ductus bursae; median unit with a slat-like, strongly sclerotized, lamella antegavinalis with smooth edges, merged ventro-laterally with lamella postvaginalis, enclosing from above the entrance to ductus bursae, with a shallow concavity; ductus bursae two-thirds the length of corpus bursae, tubular, strongly sclerotized, compressed in the middle, entrance of bursa with a strongly sclerotized bulb in ventral position; ductus seminalis originating at the entrance of bursa; bursa copulatrix oval, with two wide signa extending over two-thirds of its length.

**Fig 9:**
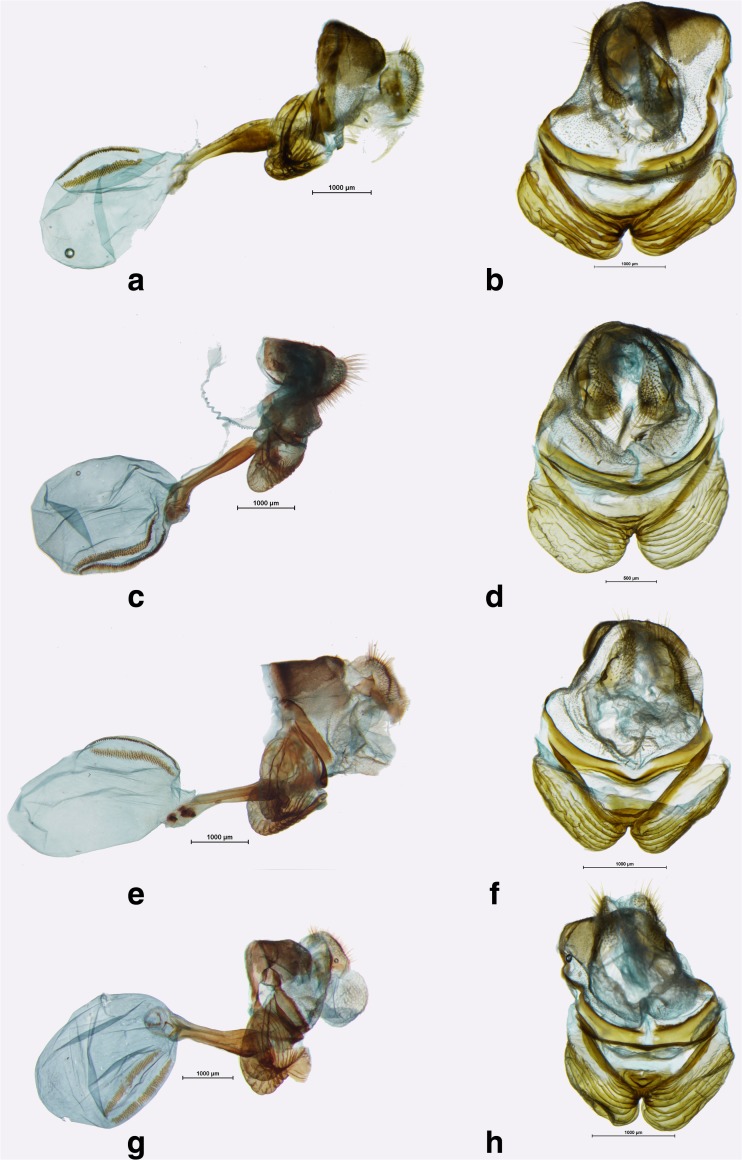
Praepedaliodes female genitalia (lateral view, ventral view): **a**-**b** *Praepedaliodes zaccae* n. sp. (Morro das Antenas, Urupema, Santa Catarina); **c**-**d** *Praepedaliodes pawlaki* n. sp. (Pico Itapeva, Pindamonhangaba, São Paulo); **e**-**f** *Praepedaliodes sequeirae* n. sp. (P. N. do Itatiaia, km 13 da estrada para Agulhas Negras, Itatiaia, São Paulo); **g**-**h** *Praepedaliodes exul* (P. N. do Itatiaia, Itatiaia, São Paulo).

**Fig 10:**
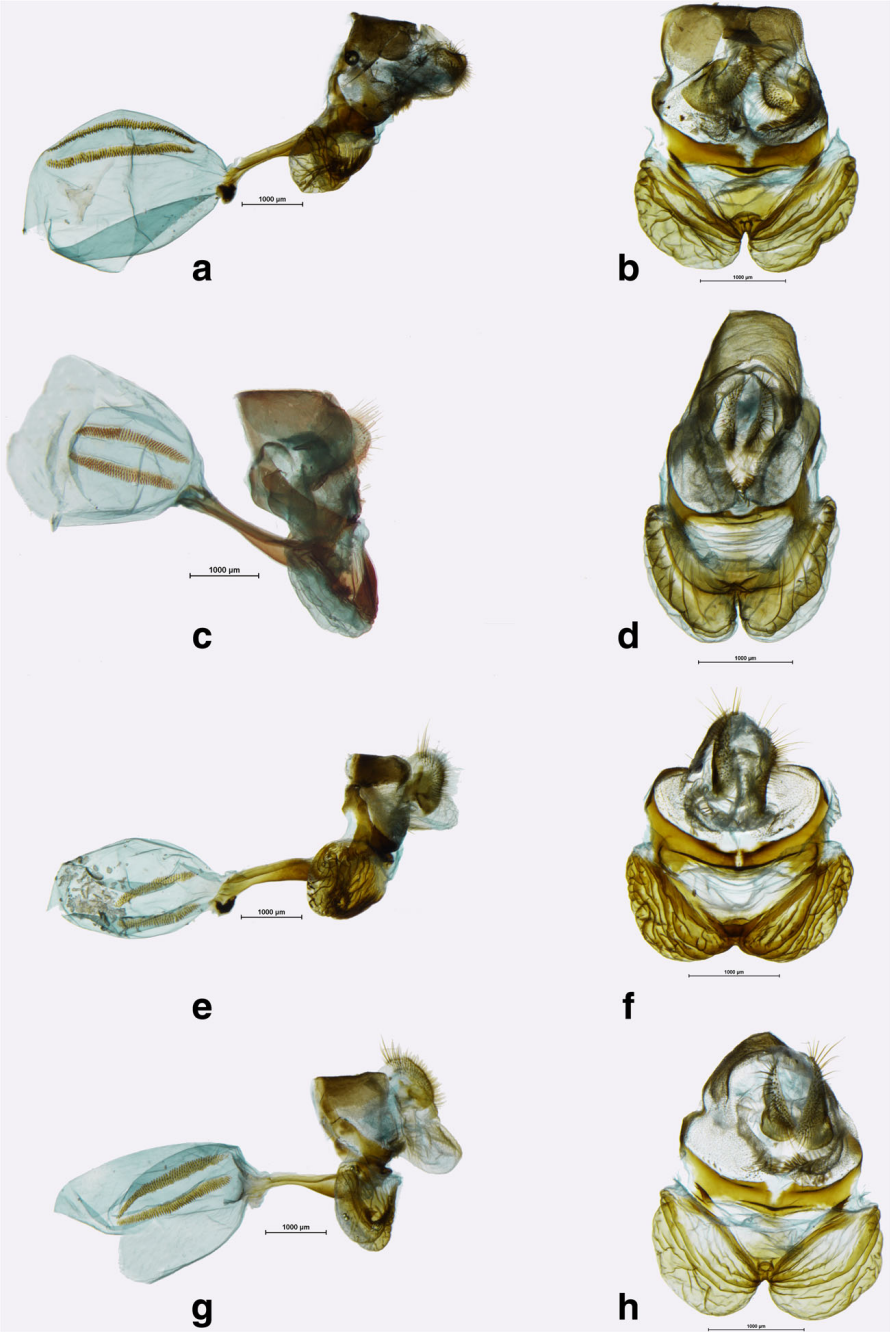
*Praepedaliodes* female genitalia (lateral view, ventral view): **a**-**b** *Praepedaliodes granulata* (Serra da Graciosa – Rio Taquari, Morretes, Paraná); **c**-**d** *Praepedaliodes amussis* (above Piquete, Piquete, São Paulo); **e**-**f** *Praepedaliodes francinii* n. sp. (Serra do Corvo Branco, Urubici, Santa Catarina); **g**-**h** *Praepedaliodes landryi* n. sp. (Pico Itapeva, Pindamonhangaba, São Paulo).

**Fig 11:**
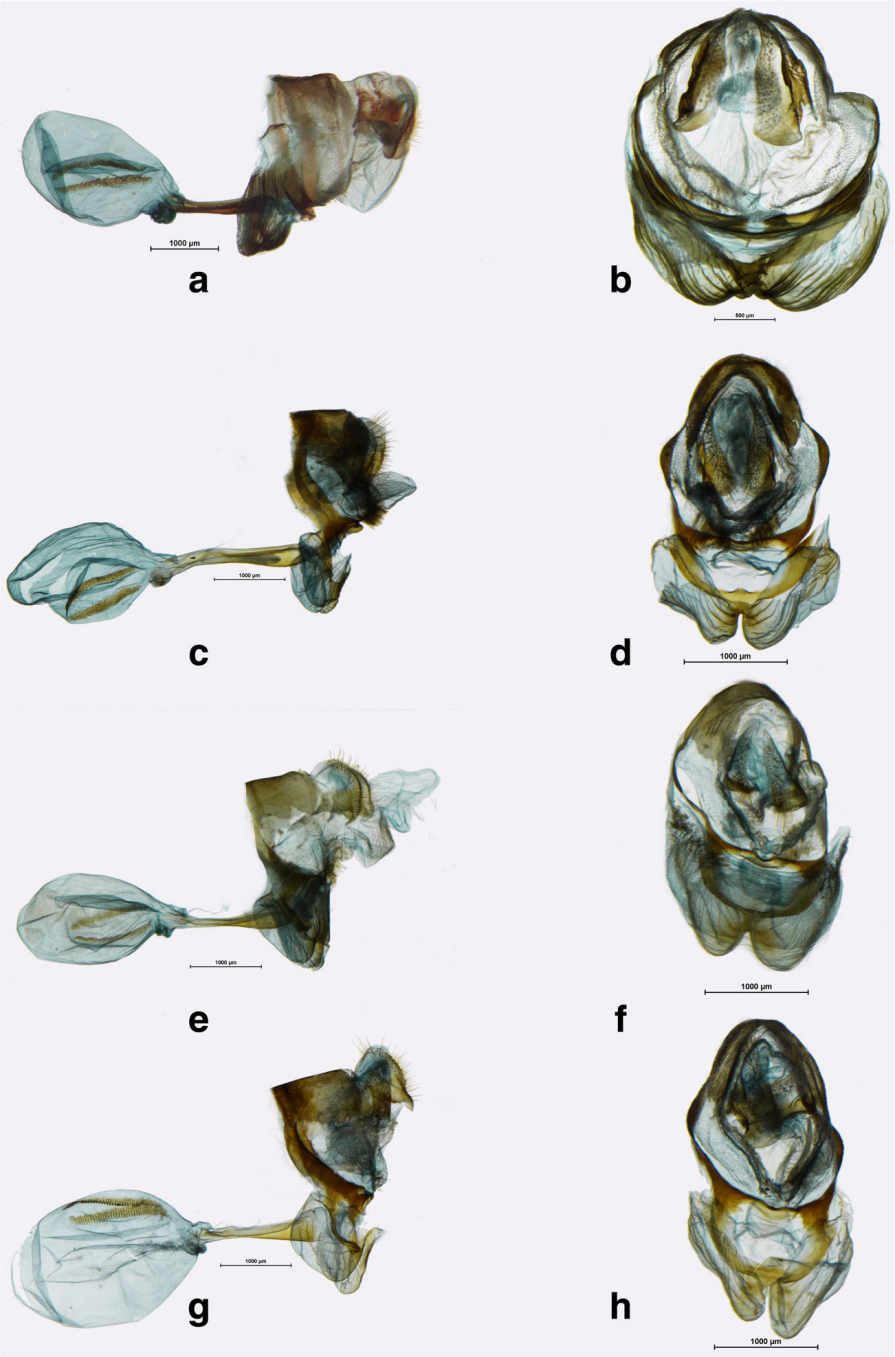
*Praepedaliodes* female genitalia (lateral view, ventral view): **a**-**b** *Praepedaliodes duartei* n. sp. (Joinville, Santa Catarina); **c**-**d** *Praepedaliodes phanias* (Serra da Graciosa – Rio Taquari, Morretes, Paraná); **e**-**f** *Praepedaliodes phanias* (Iguazu, Misiones); **g**-**h** *Praepedaliodes phanias* (Campos do Jordão, Pindamonhangaba, São Paulo).

**Fig 12:**
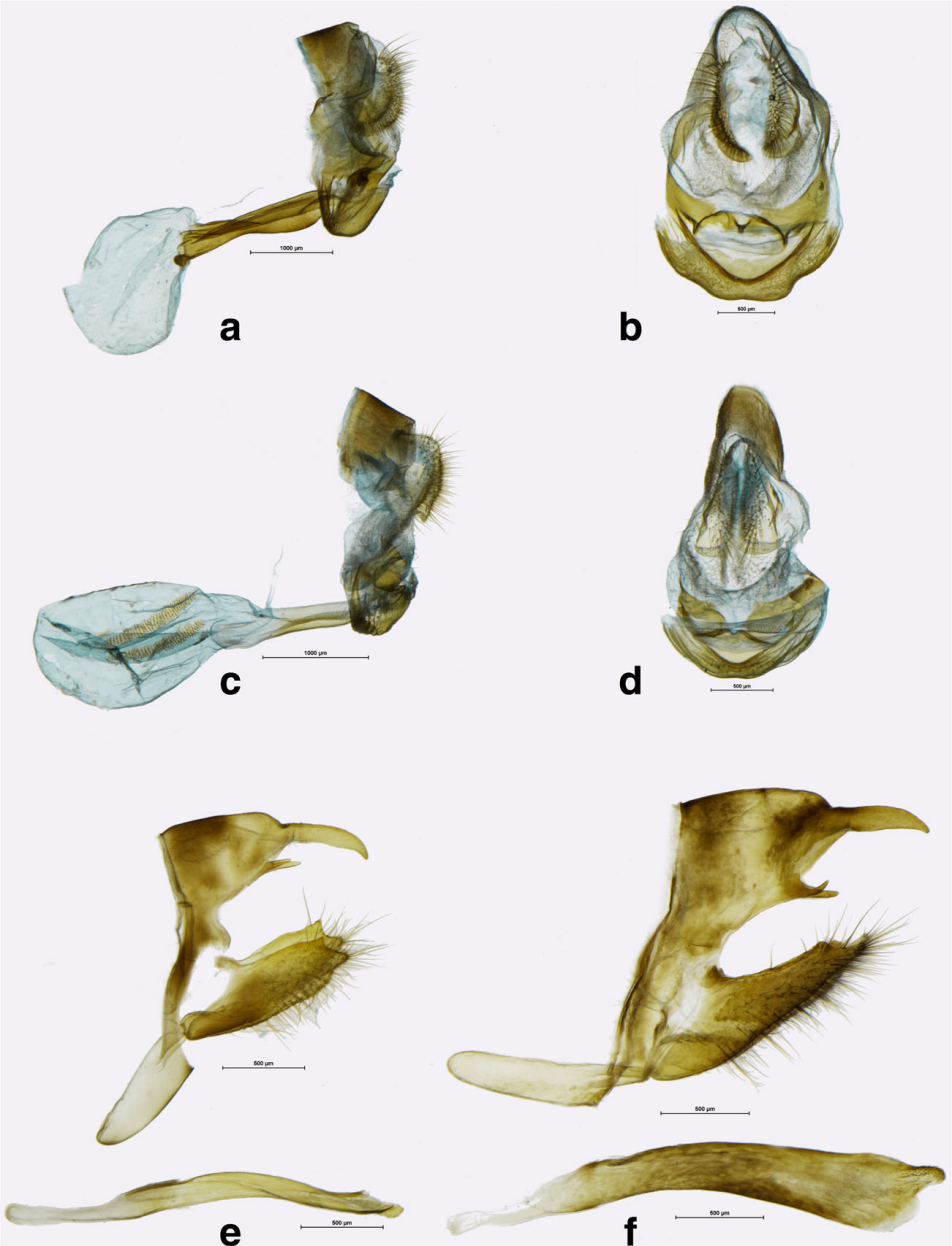
Female genitalia (lateral view, ventral view). **a**, **b** *Corderopedaliodes corderoi corderoi* (Dognin) (Valladolid, Zamora-Chinchipe, Ecaudor). **c**, **d** *Panyapedaliodes drymaea drymaea* (Hewitson) (Rio Chido – Pomacochas, Amazonas, Peru); male genitalia (lateral view, aedeagus extracted). **e** *Panyapedaliodes drymaea drymaea* (Ajanaco-Pillcopata road, Cusco, Peru). **f** *Pedaliodes petri* Pyrcz & Viloria (Bota Caucana, Cauca, Colombia).

**Fig 13:**
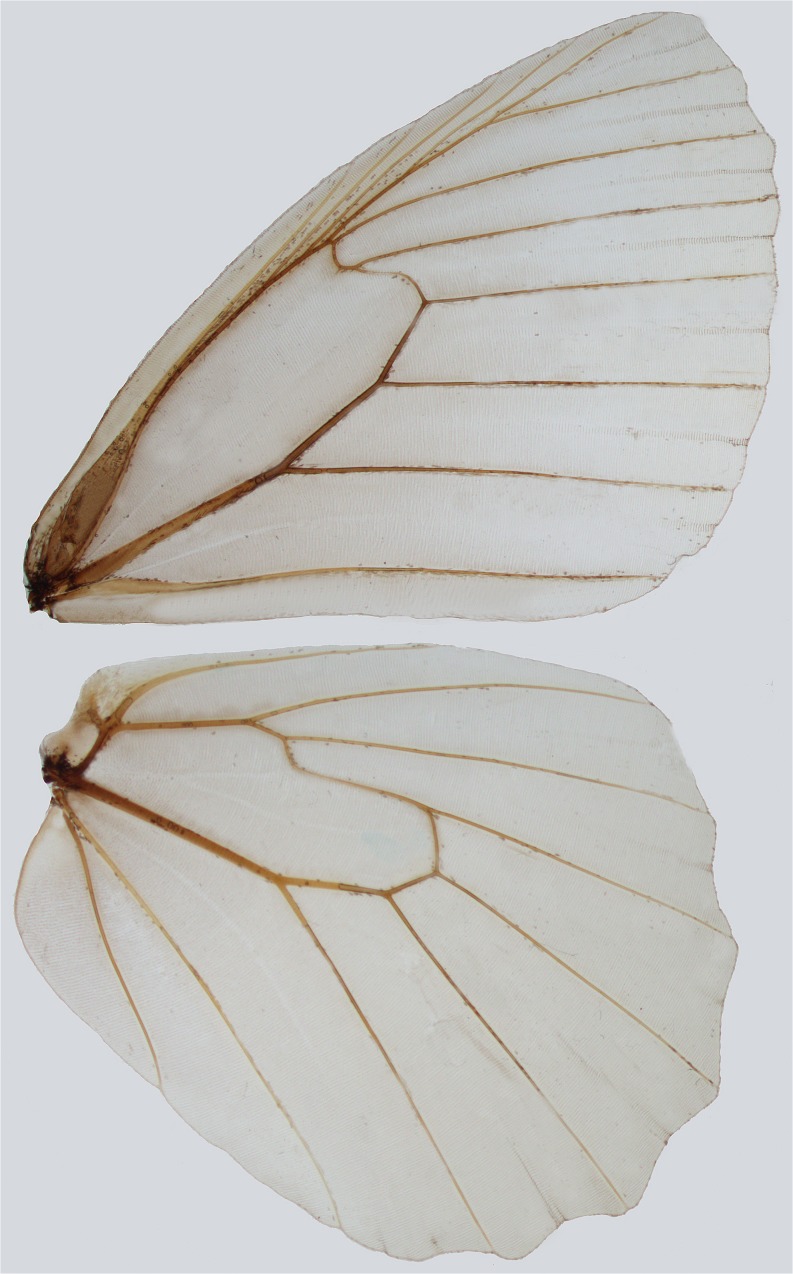
*Praepedaliodes* venation pattern (*Pedaliodes exul,* male).

### Immature stages

The following descriptions and measurements are based on material reared from a female from Serra do Japi, Jundiaí, São Paulo. Additional material from the same place and from Campinas, São Paulo, gave similar results.

**Fig 14:**
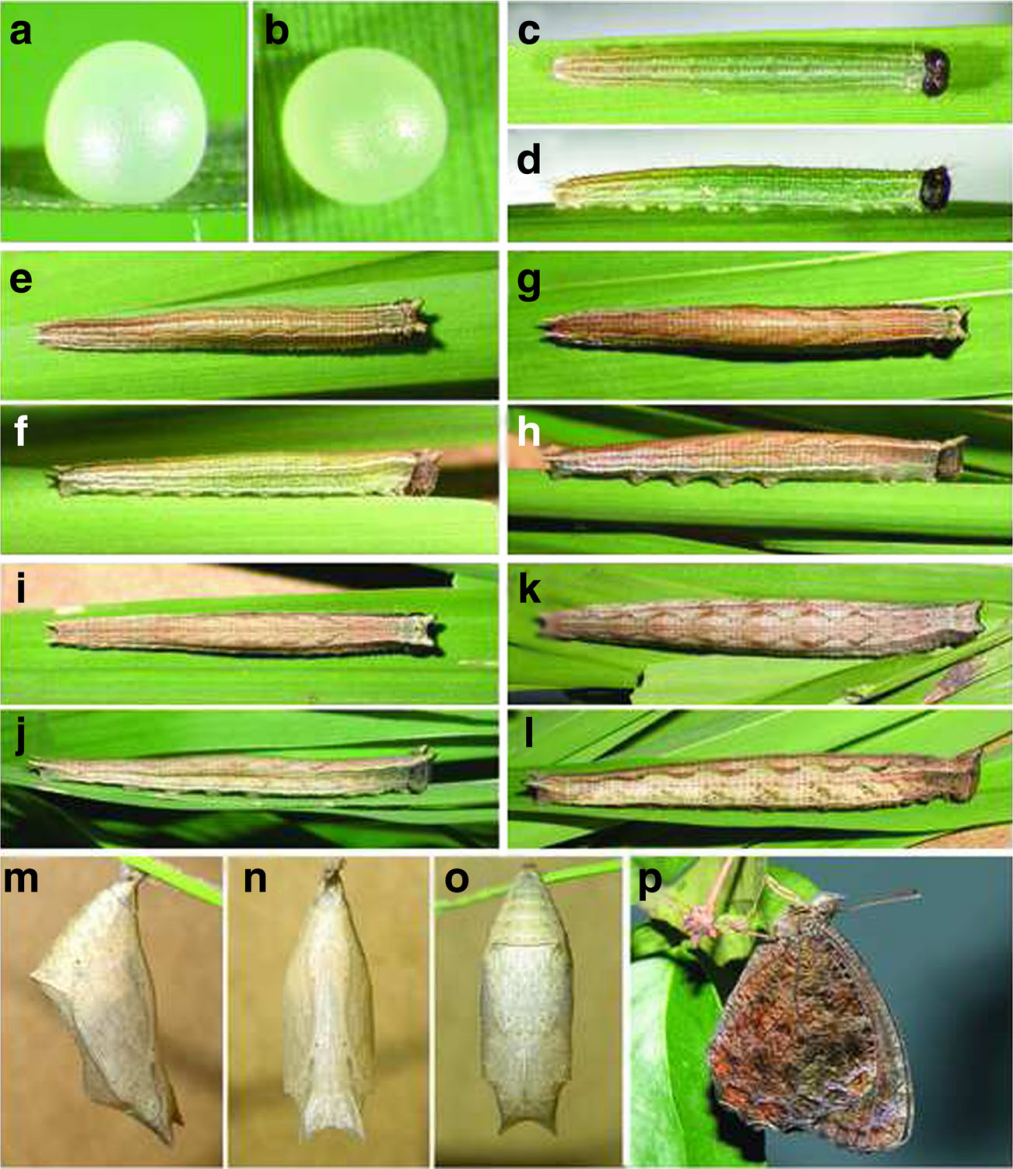
*Praepedaliodes phanias*: immature stages. **a**, **b** Egg. **c**, **d** First instar. **e**, **f** Second instar. **g**, h Third instar. **i**, **j** Fourth instar. **k**, **l** Fifth (last) instar. **m**, **n**, **o** Pupa. **p**. adult.

*Egg* (Fig 14a, b). Spherical, cream, smooth, with a reticle of thin vertical and transversal ridges barely visible under microscope. Height 1.10–1.30 mm (mean = 1.22 mm; SD = 0.076 mm; *n* = 5); diameter 1.00–1.12 mm (mean = 1.09 mm; SD = 0.052 mm; *n* = 5). Duration 4–6 days (*n* = 20).

*First instar* (Fig 14c, d). Head capsule width 0.70–0.76 mm (mean = 0.74 mm; SD = 0.020 mm; *n* = 6); head scoli 0.08–0.10 mm (mean = 0.097 mm; SD = 0.008 mm; *n* = 6). Head capsule dark brown, with enlarged chalazae, bearing a pair of short dark brown scoli on vertex, each with two long narrow setae. Third stemma larger than the other stemmata. Body light green with the last abdominal segments gradually changing to reddish, smooth, with white longitudinal stripes; caudal filaments very short. Setae light brown and elongated. Legs and prolegs light brown. Maximum length 7 mm. Duration 5 days (*n* = 20).

*Second instar* (Fig 14e, f). Head capsule width 1.04–1.12 mm (mean = 1.09 mm; SD = 0.028 mm; *n* = 6); head scoli 0.26–0.30 mm (mean = 0.28 mm; SD = 0.018 mm; *n* = 6). Head brown with two diverging short scoli on vertex. Body brown, greenish laterally, striped longitudinally with white and reddish; caudal filaments short. Legs and prolegs light brown. Maximum length 11 mm. Duration 4 days (*n* = 15).

*Third instar* (Fig 14g, h). Head capsule width 1.50–1.60 mm (mean = 1.57 mm; SD = 0.045 mm; *n* = 6); head scoli 0.40–0.46 mm (mean = 0.44 mm; SD = 0.025 mm; *n* = 6). Head dark brown, light brown on vertex, with two diverging very short scoli on vertex. Body reddish brown, laterally striped with light brown and white; caudal filaments short. Legs and prolegs light brown. Maximum length 17 mm. Duration 4–5 days (*n* = 15).

*Fourth instar* (Fig 14i, j). Head capsule width 2.16–2.30 mm (mean = 2.23 mm; SD = 0.060 mm; *n* = 6); head scoli 0.60–0.70 mm (mean = 0.64 mm; SD = 0.039 mm; *n* = 6). Head dark brown, light on vertex, with two diverging very short scoli on vertex. Body light brown, laterally striped with beige and white, with a discreet subdorsal zig-zag pattern from A1 to A6; caudal filaments short. Legs and prolegs light brown. Maximum length 24 mm. Duration 5–6 days (*n* = 15).

*Fifth (last) instar* (Fig 14k, l). Head capsule width 3.08–3.39 mm (mean = 3.14 mm; SD = 0.157 mm; *n* = 6); head scoli 0.89–1.08 mm (mean = 0.99 mm; SD = 0.077 mm; *n* = 6). Head brown, light brown on vertex, with two diverging short scoli on vertex. Body light brown, laterally striped with beige and white, with a discreet subdorsal zig-zag pattern with light green patches from A1 to A6; caudal filaments short. Legs and prolegs brown. Maximum length 37 mm. Duration 9–11 days (*n* = 10).

*Pupa* (Fig 14m, n, o ). Elongate; mostly beige; conspicuous pointed diverging ocular caps; cremaster light brown; dorsal abdomen with conspicuous transversal protruding ridge on segment A3. Total length 17 mm (*n* = 6). Duration 8–9 days (*n* = 5).

**Fig 15:**
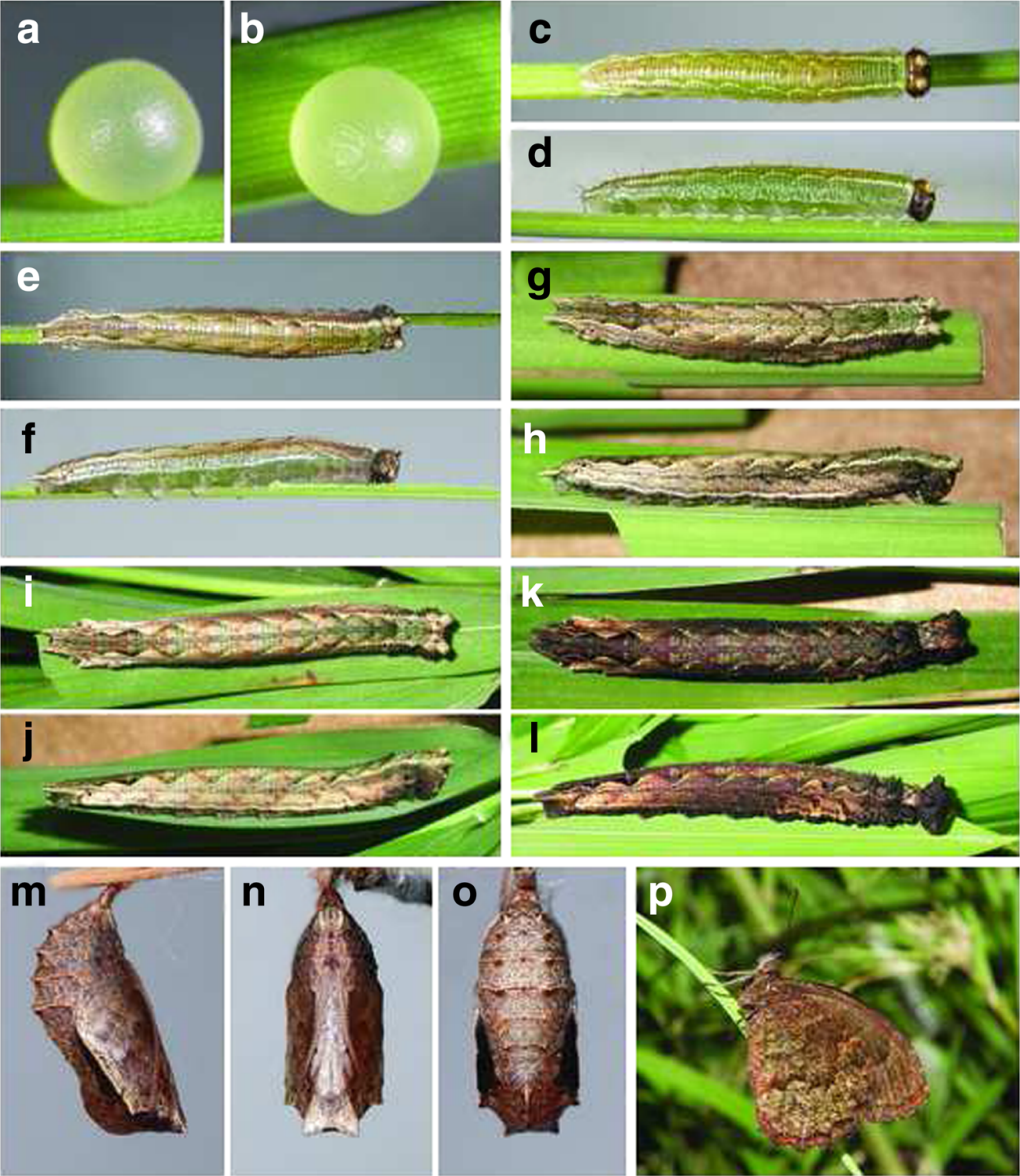
*Praepedaliodes landryi* n.sp: immature stages. **a**, **b** Egg. **c**, **d** First instar. **e**, **f** Second instar. **g**, **h** Third instar. **i**, **j** Fourth instar. **k**, **l** Fifth (last) instar. **m**, **n**, **o** Pupa. **p**. adult.

### Comments

*Praepedaliodes phanias* is the most widely distributed species of the genus. It occurs not only throughout most of southeastern and southern Brazil but also in eastern Paraguay, northern Argentina (Misiones) and Uruguay (Biezanko *et al* 1957) (Fig 19). It is more tolerant of anthropogenic activity than other congeners and persists wherever its bamboo host plants of the genus *Chusquea* exist. It possibly also feeds on secondary grasses, as it is present locally in city gardens and pastures. It is the only species of *Praepedaliodes*, alongside its closest relative *P. duartei* Dias, Dolibaina & Pyrcz n. sp., and indeed the only tropical species of Pronophilina to reach almost sea level in southeastern and southern Brazil and in Argentina. *Praepedaliodes phanias* is common and produces occasional population outbreaks when it is one of the dominant species in its environment. From this perspective, it can be compared ecologically to *Pedaliodes manis* (C. Felder & R. Felder, 1867) in the northern Andes, *Panyapedaliodes drymaea* (Hewitson, 1858) in Colombia and Ecuador and *Pedaliodes palaepolis* (Hewitson, 1878) in Peru.

Viloria (unpublished data) separated from *P. phanias* two supposedly new allopatric species mostly based on male genitalia differences. The examination of the available material, including specimens examined by the above cited author deposited in the NHMUK, shows, however, a great deal of variability in the male genitalia of *P. phanias* across the species geographic range. Consistent morphological characters supporting more than a single species could not be found. It is possible that further studies will demonstrate a more intricate geographical pattern and that stable subspecies could be identified, including the supposedly new taxa recognized by Viloria (unpublished data). This, however, requires more additional morphological, molecular, and ecological data than are currently available.

***Praepedaliodes granulata*** (Butler, 1868)

(Fig 2c, d, 5c, d, 10a, b and 20)

**Fig 16:**
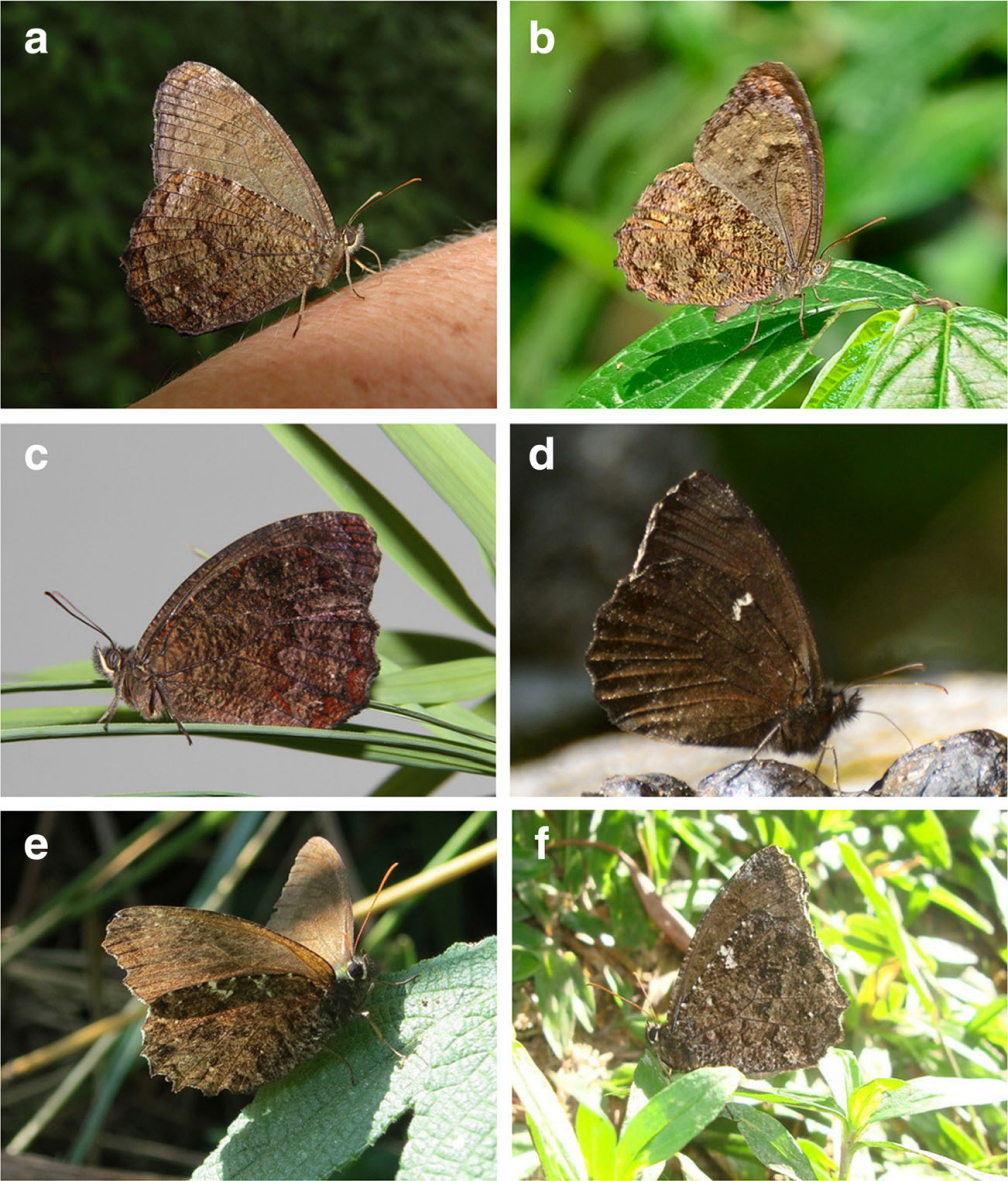
Individuals in natural habitat. **a** *Praepedaliodes phanias*, male, Iguazu (photo Kim Garwood). **b** *Praepedaliodes landryi* n. sp., female, Paranapiacaba (photo André Freitas). **c** *Praepedaliodes* landryi, reared female, Paranapiacaba (photo André Freitas). **d** *Praepedaliodes exul*, male, P. N. Itatiaia (photo Kim Garwood). **e** *Praepedaliodes amussis*, female, P. *N. Serra* da Bocaina (photo Pierre Boyer). **f** *Praepedaliodes amussis*, male, Itatiaia (photo Kim Garwood).

*Pedaliodes perperna* C. Felder & R. Felder 1867 p. 468 (preocc. *Pedaliodes perperna* Hewitson, 1862)

*Pedaliodes granulata* Butler, 1868: 173, pl. 4, fig 8; Kirby, 1871: 104; Thieme, 1905: 77, 79; Riley & Gabriel, 1924: 23; D’Abrera, 1988: 852, figs [9–10]

*Pedaliodes phanias* form *granulata*; Weymer, 1912: 254, pl. 54, row a (as *P. phanias*)

*Pedaliodes phanias* var. *granulata*; Gaede, 1931: 497.

*Muscopedaliodes granulata*; Forster, 1964: 154, fig 187 (male genitalia, erroneous)

*Praepedaliodes granulata*; Adams, 1986: 276; Lamas *et al* 2004: 214; Pyrcz, 2010: 242

Type locality: “Bogotá, Colombia”, [Brazil]

Type material: *Pedaliodes granulata* Butler, 1868 was described based on an unstated number of specimens deposited in the NHMUK. One female syntype with the following labels is here designated lectotype to confirm the identity of the species: / Children’s Sale, 40. 4.2 880 (2062a)/ BMNH type No. Rh 3968, *Pedaliodes granulata* Butl. ♀/ lectotype/ *P. granulata* Butler type/. Characteristic lectotype labels will be added to the specimen.

### Material examined

BRAZIL: *São Paulo*: **Salesópolis** (Estação Biológica de Boracéia), 1 ♀ 06.XII.1941, ex coll. D’Almeida (DZ 23.444), 1 ♂ and 1 ♀ 09.X.1943, D’Almeida *leg.*, ex coll. D’Almeida (DZ 19.805, DZ 23.434 prep. genit. D. Dolibaina 2010) (DZUP), 2 ♂ 24.I.1968, R. Travassos *leg.* (MZUSP), 850 m, 1 ♂ and 1 ♀ 07.III.1968, J. Oliveira Santos *leg.* (MZUSP), 1 ♀ 07.I.1969, J. Oliveira Santos *leg.* (MZUSP), **Santo André** (Parque das Nascentes de Paranapiacaba), 1100–1200 m, 1 ♂ (prep. genit. E. P. Barbosa 2012) 18.XI.2006 (DNA voucher BLU 659), M. Uehara-Prado, A. V. L. Freitas & K. S. Brown Jr. *leg.* (ZUEC LEP 9955) (ZUEC). *Paraná*: **Balsa Nova** (São Luis do Purunã), 950 m, 1 ♂ 15.II.1988, Mielke *leg.* (OM 17.012) (OM). **Morretes** (Alto da Serra), 800 m, 1 ♀ 18.IV.1988, Mielke *leg.* (OM 17.222), 1 ♀ 19.II.1989 (OM 21.401) (OM), (Serra da Graciosa, Rio Taquari, S25°19′ W48°56′), 850–900 m, 1 ♂ 31.I.2015, P. Boyer *leg.* (MZUJ), 6 ♂ and 1 ♀ 01.II.2015, T. Pyrcz *leg.* (prep. genit. 306/16.12.2015 J. Lorenc) (MZUJ) [♂ Fig 2c, ♀ Fig 2d], 3 ♂ and 2 ♀, P. Boyer *leg.* (MZUJ); 1 ♂ 02.II.2015, P. Boyer *leg.* (MZUJ), (Serra da Graciosa, 5 km N Hotel), 870–880 m, 1 ♂ and 1 ♀ 31.I.2015, T. Pyrcz *leg.* (MZUJ), (Casa de Pedra), 900–920 m, 2 ♀ 02.II.2015, T. Pyrcz *leg.* (prep. genit. 01/10.06.2015 J. Lorenc) (MZUJ), (Serra da Graciosa) 850 m 2 ♂ 01.II.2015 (DNA vouchers BLU 713, BLU 714), T. W. Pyrcz *leg.* (ZUEC LEP 9953, ZUEC LEP 9954) (ZUEC). **Tijucas do Sul** (Morro do Araçatuba), 2 ♂ and 1 ♀ 24-25.IV.2007, D. Dolibaina *leg.* (prep. genit. 06/10.06.2015 J. Lorenc, prep. genit. 03/25.08.2012 J. Lorenc) (MZUJ). *Santa Catarina*: **Joinville**, 200 m, 1 ♀ 5.IV.1980, Mielke & Miers *leg.* (DZ 23.424), 100 m, 1 ♂ 25.II.1981, H. Ebert *leg.*, ex coll. H. Ebert (DZ 19.433 prep. genit. D. Dolibaina 2010) (DZUP). *Rio Grande do Sul*: **São José do Hortêncio** (Rio Cadeia), 1 ♀ 03.III.1991, A. Moser *leg.* (CLAM). No *data*: « Río Gran », 1 ♂, Hewitson ex-coll., (NHMUK).

### Redescription

*Male* (Fig 2c). *Head*. Antennae reaching 2/5 the length of costa, slender, dorsally dark brown, covered with rather sparse, minute, silver scales, ventrally orange-brown, mostly naked, except for a few basal flagellomeres with sparse milky white scales, club naked, of 14 flagellomeres, slightly thicker than shaft, ventrally light orange; eyes blackish brown, densely hairy; labial palpi two times the length of head, covered mostly with blackish hairy scales and hair, considerably longer ventrally, except for a lateral row of sandy yellow scales; collar with sandy yellow scales. *Thorax.* Black, covered with golden brown, long scales, tegulae as well; legs brown, tibiae and tarsi covered with grey-brown scales. *Wings.* FW (length: 28–30 mm) apex blunt, outer margin almost straight, slightly wavy towards apex; fringes mostly brown except for some sandy yellow scales in the interspaces; FWD uniform blackish brown, lustrous; androconial patch slightly darker than the ground colour, covering median one-third of wing surface, from discal cell distal edge to anal margin, entering discal cell. FWV cedar brown, lighter and duller than on the dorsal surface; a wave postdiscal line extending from costal margin to CuA_1_ marked basally by dark brown scaling; thinner and less wavy, chocolate brown submarginal and marginal lines; apical area dusted with grey brown scales. HW oval with an undulated outer margin; fringes mostly grey brown, with some sandy yellow, sparse scales; HWD uniform blackish brown, lustrous, sparsely hairy in median half and along anal margin. HWV colour pattern marbled, composed of various shades of brown scales, none of which predominates, except for a concentration of chocolate brown scales in postmedian area, making up an irregular postdiscal line extending from costal to anal margin, shallowly, yet noticeably incurved basally in space CuA_1_-CuA_2_, and a chocolate and golden brown submarginal line; in some individuals a series of minute whitish submarginal dots, one in each space, in others not apparent. *Abdomen.* Dorsally covered with dark brown scales, ventrally and laterally with greyish brown scales. Genitalia (Fig 5c, d). Tegumen compressed and elongated in lateral view, dorsum gently arched; uncus two-thirds the length of tegumen dorsum, slender, ventrally slightly constricted near base, and slightly arched downwards in the middle, with a subacute tip; subunci parallel to uncus, slender, approximately the same width throughout, tip sharp; pedunculus short; vinculum wide, then gradually narrowing towards saccus base; saccus moderately deep, the length of uncus, as wide as vinculum in its widest part; valva the length of tegumen + uncus, wide in basal area, slender in distal two-thirds, with a series of prominent teeth-like processes on dorsal surface from base to distal two-thirds, then smooth, terminated with and prominent, sharp, upstanding subapical process, tip blunt; aedeagus slender, the length of saccus + valva, nearly straight, with a sharp tip, smooth, spoon-like at base, proximal opening two-thirds the length of aedeagus.

*Female* (Fig 2d). Sexual dimorphism is slight and expressed in the larger size of the female (FW length: 30–33 mm) and the slightly lighter wing ground colour, chocolate brown instead of blackish and cedar brown. Genitalia (Fig 10a, b). Genitalia flattened laterally in ventral view; papilla analis small, produced in the middle in lateral view, covered with dense and delicate setae; proximal unit, in lateral view, consisting of a weakly developed postvaginal lamella transforming gradually into two, rather well sclerotized prominent lateral pocket-like folds with a strongly rippled surface, compressed towards the entrance of ductus bursae; median unit with a wide, slat-like, strongly sclerotized, lamella antevaginalis with smooth edges, touching, but not merging ventro-laterally with lamella postvaginalis, enclosing from above the entrance to ductus bursae, where producing a deep incision doubled with a shallow concavity; ductus bursae half the length of corpus bursae, tubular, strongly sclerotized, slightly compressed in the middle with a strongly sclerotized bulb in ventral position at entrance of bursa; ductus seminalis originating at the entrance of bursa; bursa copulatrix oval, with two wide signa extending over two-thirds of its length.

### Comments

There is considerable confusion about the identity and the type locality of *Praepedaliodes granulata*. In the original description, Butler (1868) stated that the species was already described by C. Felder & R. Felder (1867) under the name *Pronophila perperna*, however this name is preoccupied by *Pronophila perperna* Hewitson, (1862). He consequently did not describe in extenso a new species but merely quoted, actually imprecisely, C. Felder & R. Felder’s (1867) description, stating “larger than *P. proerna* Hewits., the margins of the wings more deeply sinuated”, and, in doubt, repeated the type locality specified by the Felders as “Bogota”. Here, nevertheless, Butler’s (1868) description is considered valid. There are two specimens of *P. granulata* at the NHMUK, a male with a locality label “Rio Gran.” (possibly Rio Grande do Sul), and a female with no locality data, but previously recognized as a syntype by the curators of the NHMUK. The male is not considered a syntype of *P. granulata*, since the illustration in Butler’s paper is of a female underside. This female specimen is here designated lectotype. The syntype of *Pronophila perperna* C. Felder & R. Felder 1867 is a male specimen. The correct identification of this species is difficult, especially because there are at least two other species with similar colour patterns but consistently different genital structure (*P*. *phanias* and *P. duartei* n. sp.), and thus there are numerous misidentifications in museums. In addition, Forster (1964) obviously misidentified this species as the genitalia he illustrated as belonging to *P. granulata* (Forster 1964: 154, fig 187) is closely similar to *P. amussis*, whereas those of true *P. granulata* are markedly different.

*Praepedaliodes granulata* females are the largest butterflies among *Praepedaliodes* alongside the females of *P. duartei* n. sp. Historical specimens have vague label data and are not helpful with the identification of the geographic range of *P. granulata*. Until recently *P. granulata* was known only from the Serra do Mar and the Serra da Bocaina in the São Paulo state. However, field research revealed its presence further south in Paraná and Santa Catarina. *Praepedaliodes granulata* does not seem, however, to occur in the parallel Serra da Mantiqueira (Fig 20). Further sampling is needed to establish its range limits both to the north in Rio de Janeiro or even in Espirito Santo, and to the south of Santa Catarina. *Praepedaliodes granulata* occurs at low to intermediate elevations, mostly around 100–1200 m in very dense and well preserved forest where isolated patches of bamboo are present. It is reluctant to stray into open space, and whenever individuals of *P. granulata* have to cross a path or clearing, they fly fast and erratically, clearly looking alarmed. It is uncommon and rarely observed due to its shy behaviour and hardly accessible habitats.

***Praepedaliodes duartei*** Dias, Dolibaina & Pyrcz **n. sp.**

(Fig 2a, b, 8a, b, 11a, b and 21)

Type locality: Rerserva Serra Bonita, Camacan, Bahia, Brazil

### Type material

*Holotype* ♂ with the following labels: /Camacan, Reserva Serra Bonita, Bahia: Brazil, 15°23′S 39°33′W, VIII.2009, 200 m, F. L. Santos col. / DNA voucher BLU 550 / ZUEC LEP 9948 / (prep. genit. E. P. Barbosa 2015) / Deposited in the Museu de Zoologia Adão José Cardoso, Universidade Estadual de Campinas, Campinas, São Paulo, Brazil (ZUEC).

*Paratypes*. (9 ♂ and 31 ♀): BRAZIL: *Espírito Santo*: **Santa Teresa**, 1 ♀ 4.VI.1967, C. Elias & T. Elias *leg.* (DZ 35.109) (DZUP). *São Paulo*: **Salesópolis** (Estação Biológica de Boracéia), 1 ♀ 29.II.1969, R. Travassos *leg.* (MZUSP). **São Paulo**, 1 ♂ (MZUSP 56.254) (MZUSP), (Serra da Cantareira), 900–1100 m, 1 ♀ VI.1948, Wucherpfennig *leg.*, ex coll. H. Ebert (DZ 35.045) (DZUP). **Guarujá**, 1 ♀ 17.VII.1977, A. Moser *leg*. (CLAM). *Paraná*: **Balsa Nova**, 1 ♂ (São Luis do Purunã), 1000 m, 7-8.VII.2007, Beltrami *leg.* (DZ 23.354 prep. genit. D. Dolibaina 2010) (DZUP). **Guaratuba** (Pontal do Itararé), 950 m, 1 ♀ 22.II.2005, Mielke *leg.* (DZ 33.400), 1400 m, 1 ♀ 9.II.2007, Mielke *leg.* (DZ 20.312 prep. genit. D. Dolibaina 2010) (DZUP). **Morretes** (Alto da Serra), 800 m, 1 ♂ 07.II.1989, Mielke *leg.* (OM 21.956) (OM), 1 ♀ 15.IV.2013, Mielke & Callaghan *leg.* (DZ 35.130) (DZUP), (Morro Alto), 300 m, 1 ♂ 12.IV.2003, Mielke *leg.* (OM 60.822) (OM). **Piraquara** (Mananciais da Serra), 800 m, 1 ♀ 29.III.2007, Mielke & Casagrande *leg.* (DZ 23.845) (DZUP). **São José dos Pinhais** (BR 277, km 54, Torre Telepar, 25°33′18″S 48°58′22″W), 1060 m, 1 ♀ ACD, PROAC & RRC *leg.* (DZ 35.090) (DZUP). **Tijucas do Sul** (Morro do Araçatuba), 1600 m, 1 ♀ 25.II.2011, P. Grossi *leg.* (MZUJ) [♀ Fig 2b], (Morro do Araçatuba, base), 900–920 m, 1 ♀ 900–920 m, 03.II.2015, T. Pyrcz *leg.* (MZUJ), (Vossoroca), 850 m, 1 ♀ IV.1971, Moure & Mielke *leg.* (DZ 35.100), 1 ♀ 8.III.1981, Mielke *leg.* (DZ 35.065) (DZUP). *Santa Catarina*: **Ituporanga** (Cruzeiro do Sul), 1 ♂ 1901, ex coll. H. Ebert (DZ 33.257 prep. genit. F. Dias 2016) (DZUP). **Joinville** 1 ♂ and 8 ♀, ex coll. Staudinger & Bang-Haas, Ankauf 1961 (prep. genit. 02/09.11.2010 J. Lorenc, prep. genit. 02/01.09.2008 T. Pyrcz), 1 ♂, ex coll. A. Chaminade (prep. genit. 07/13.04.2015 J. Lorenc) (PBF) [♂ Fig 2a], 2 ♀, ex coll. D’Almeida (1 ♀ DZ 27.376 prep. genit. F.M.S. Dias 2012) (DZ 27.376, DZ 35.059), 1 ♂ VIII, Pohl *leg.*, ex coll. H. Ebert (DZ 23.364 prep. genit. D. Dolibaina 2010), 1 ♀ 27.X.1968, Mielke *leg.* (DZ 19.307 prep. genit. D. Dolibaina 2010), 1 ♀ 22.IV.1972, Mielke & Miers *leg.* (DZ 35.119) (DZUP), 1 ♀ 31.XII.1991, Mielke & Miers *leg.* (OM 28.479) (OM). **Rio dos Cedros** (Alto Rio dos Cedros), 650 m, 1 ♀ 4.II.1972, Lauterjung *leg.* (DZ 35.140) (DZUP). **São Bento do Sul**, 2 ♀ 9.VIII.2014, Rank leg (DZ 35.079, DZ 35.261), 1 ♂ 12.VIII.2014, Rank *leg.* (DZ 35.049) (DZUP), (Rio Natal), 500 m, 1 ♂ 20.IV.2002, Mielke, Rank & Casagrande *leg.* (OM 55.600) (OM), 1 ♀ 01.VII.2004, Rank *leg.* (OM 65.495). (Rio Vermelho), 850 m, 1 ♀ 26.III.1980, Rank *leg.* (DZ 33.237), 1 ♀ 15.III.1981, Rank *leg.* (DZ 33.327) (DZUP).

### Diagnosis

All brown upperside as in other sympatric congeners; differs from most *Praepedaliodes* by its considerably larger size, and more scalloped HW margins. The only species which matches its size is *P. granulata*, which is however darker, without any beige or grey pattern elements, and in addition *P. duartei* n. sp. presents a row of rather conspicuous FWV yellowish submarginal dots that are not apparent in *P. granulata*.

### Description

*Male* (Fig 2a). *Head.* Antennae reaching 2/5 the length of costa, slender, dark brown, ventrally orange, naked, club of 12 flagellomeres, only slightly thicker than shaft, ventrally orange; eyes chocolate brown, densely hairy; labial palpi two times the length of head, covered ventrally with blackish brown, and sandy yellow lateral hairy scales; collar of orange brown and dark brown elongated scales. *Thorax.* Dorsally and ventrally black, covered with rather sparse brown and golden scales, tegulae covered with brown scales with a dark blue sheen; legs brown, femur, tibia (covered with dense spines) and tarsus covered with brown and grey yellow scales. *Wings.* FW (length: 29–31 mm) with a blunt apex and straight outer margins, marginally truncate below apex; fringes very short, intermittently brown and sandy yellow; FWD uniform chocolate brown, lustrous; androconial patch a shade darker, large, covering median one-third of wing surface, from discal cell distal edge to anal margin, including distal half of discal cell. FWV dull, medium brown, lighter than on the dorsal surface; a sinuate, postdiscal darker brown line and a submarginal, thinner dark brown line; a series of faint, barely noticeable subapical, yellowish dots; some lilac scales in the apical area. HW rounded with an undulated outer margin; fringes very short, brown; HWD uniform chocolate brown, lustrous, sparsely hairy in basal and postbasal area. HWV dull, medium brown, liberally speckled with chocolate brown scales, with a sinuous postdiscal line edged basally with chocolate brown, distinctly outcurving in cell M_2_-M_3_, and a chocolate brown marginal areas. Abdomen: black, dorsally covered with dark brown, ventrally and laterally with grey brown scales. *Male genitalia* (Fig 8a, b). Tegumen massive, triangular in lateral view, dorsum slightly arched; uncus the length of tegumen dorsum, slender, ventrally strongly constricted near base, and slightly arched downwards in the middle, with a sharp tip; subunci massive, wide at base and strongly adhered to tegumen, gradually narrowing towards a sharp tip, three-fourths the length of uncus, lifted upwards; pedunculus short and wide; vinculum slender; saccus deep, the length of uncus, two times as wide as vinculum in lateral view; valva slender, the length of tegumen + uncus, only slightly wider at base than towards apex, with a series of sharp processes along distal half of dorsal surface, of approximately the same size, except for the subapical process which is generally slightly longer than the remaining ones, apex blunt; aedeagus slender, longer than saccus + valva, nearly straight, with a sharp tip, smooth, spoon-like at base; proximal opening half the length of aedeagus.

*Female* (Fig 2b). Sexual dimorphism is slight except for the consistently larger size (29–33 mm), lighter brown, and more prominent HWV speckling. Genitalia (Fig 11a, b). Flattened laterally in ventral view; papilla analis prominent, gently rounded in lateral view, covered with sparse, short, and delicate setae; proximal unit, in lateral view, consisting of a well-sclerotized slat-like, smooth lamella postvaginalis extending into two, moderately sclerotized, prominent lateral pocket-like folds with a delicately rippled surface, compressed towards the entrance of ductus bursae; median unit with a slat-like, strongly sclerotized, lamella antegavinalis with smooth edges, touching but not merging ventro-laterally with lamella postvaginalis, enclosing from above the entrance to ductus bursae, with a deep incision and a shallow concavity; ductus bursae half the length of corpus bursae, tubular, strongly sclerotized, wide and approximately the same width throughout, entrance of bursa with a strongly sclerotized bulb in ventral position; ductus seminalis originating at the entrance of bursa; bursa copulatrix oval, with two wide signa extending over two-thirds of its length.

**Fig 17:**
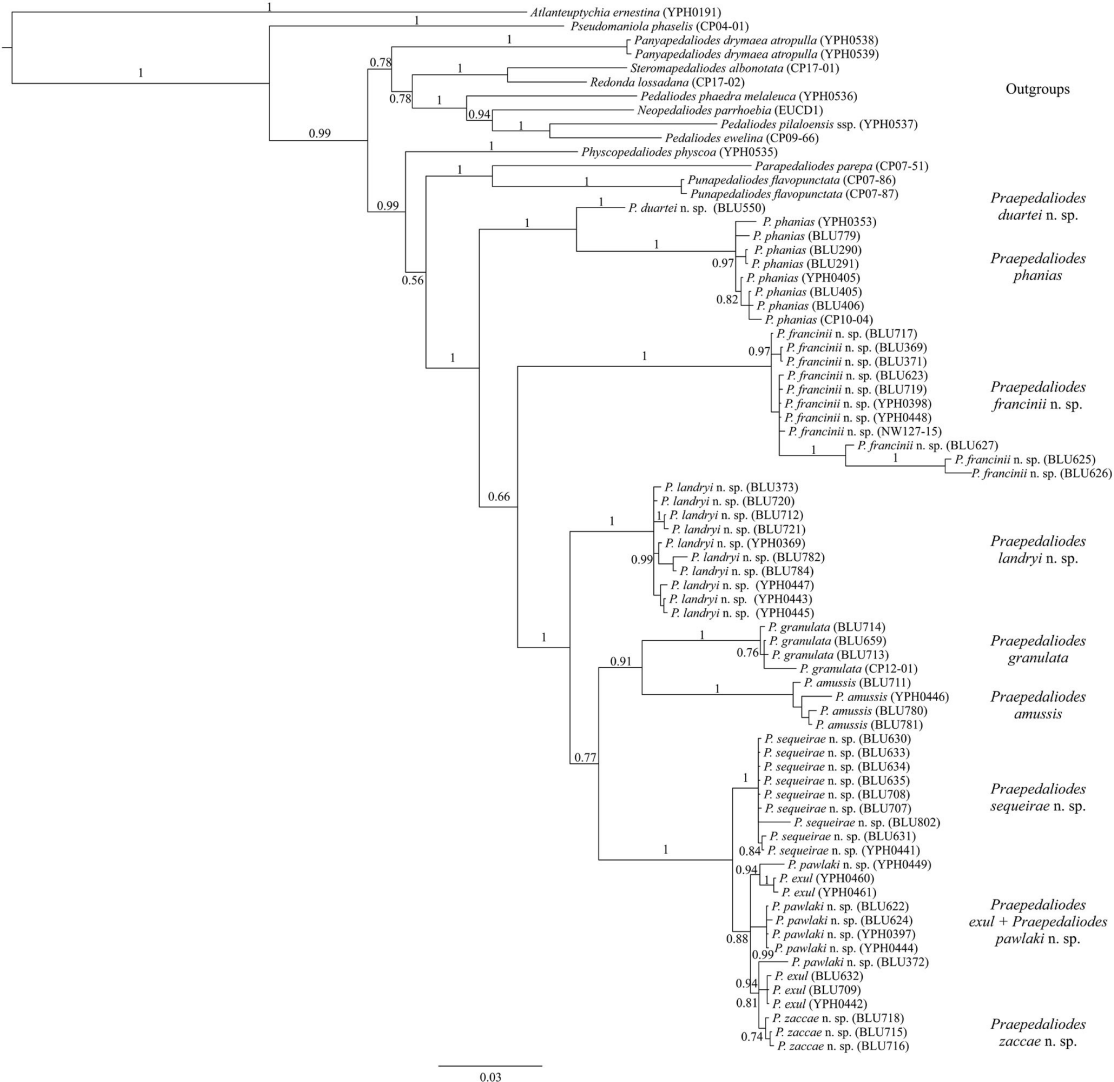
Phylogenetic relationships among the 10 known species of *Praepedalidodes* based on DNA sequences of COI and obtained by Bayesian inference. Posterior probabilities of nodes are given near the branches.

**Fig 18:**
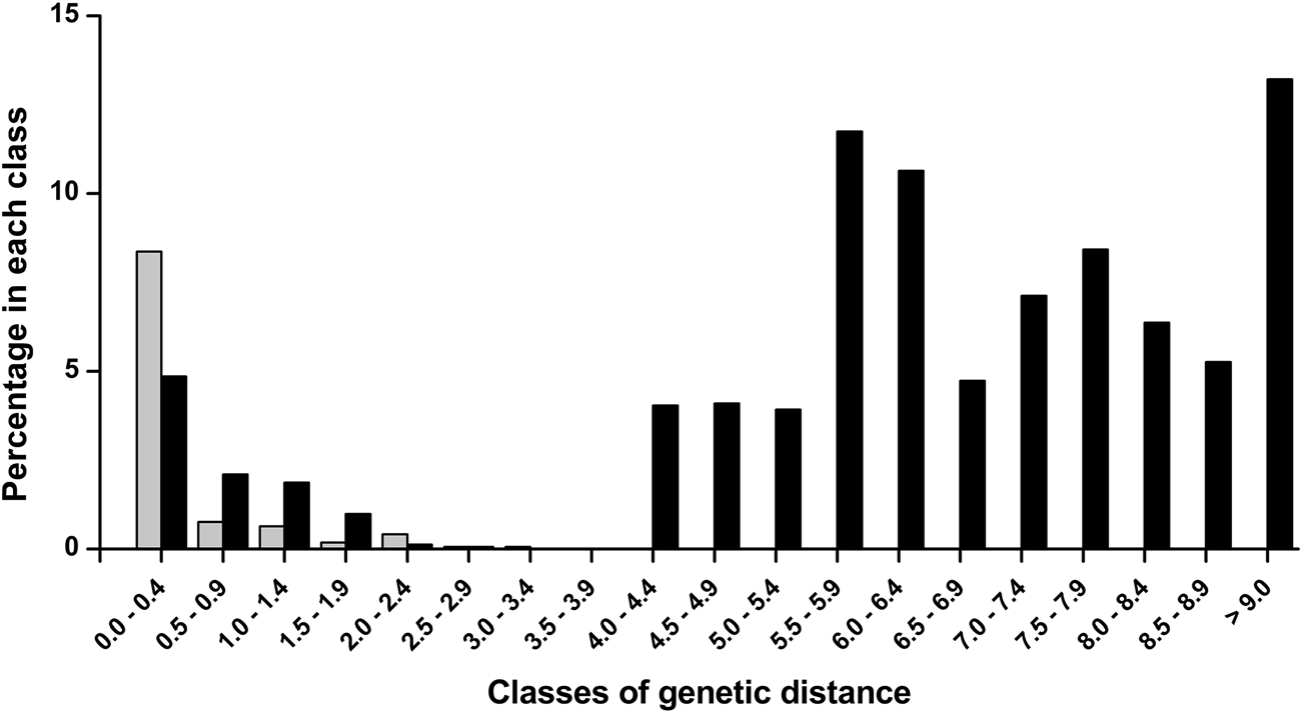
Frequency distribution of pairwise individual genetic distances within (*gray*) and between (*black*) the 10 species of *Praepedaliodes.*

**Fig 19:**
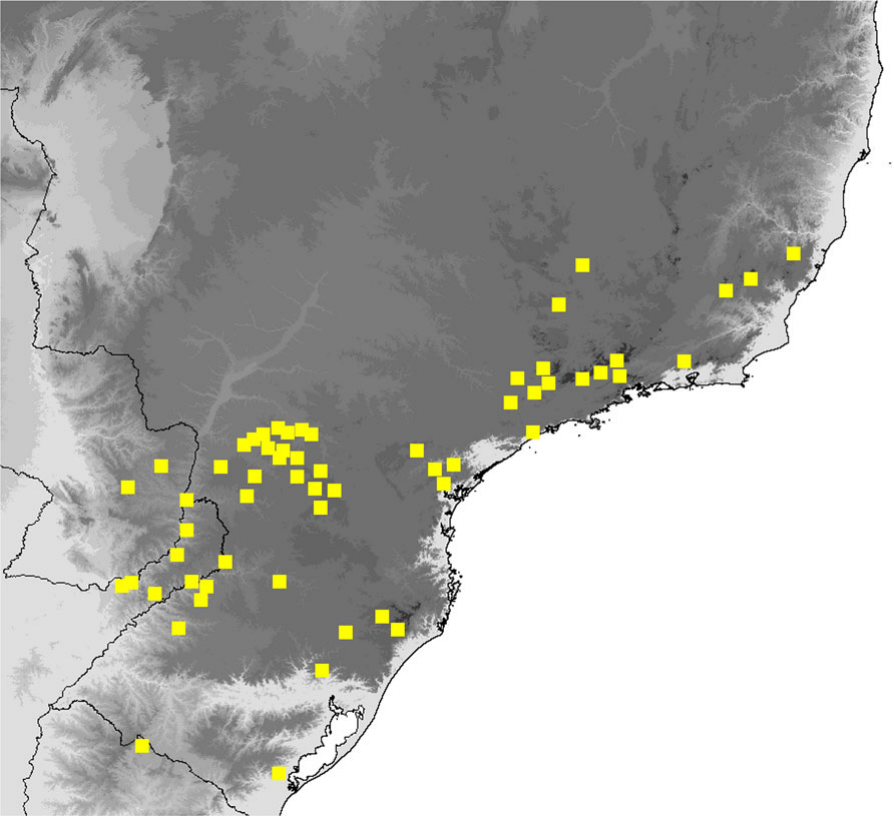
*Praepedaliodes phanias* distribution map.

### Etymology

This species is dedicated to the Brazilian entomologist Dr. Marcelo Duarte, curator of Lepidoptera from Museu de Zoologia da Universidade de São Paulo (MZUSP).

### Comments

*Praepedaliodes duartei* n. sp. has arguably the most unusual distribution pattern of all *Praepedaliodes*. It is found exclusively along the Atlantic coast, and as suggested by some label data, at atypically low elevations for this genus. Reliable collecting data are, however, very few and geographically scattered (Fig 21). The largest known series comes from a local collector who worked for Staudinger in Joinville, Santa Catarina. Other data are consistent with low elevations, with one confirmed report (200 m) from southern Bahia, being also the northernmost known locality for the genus. *Praepedaliodes duartei* n. sp. is most closely related to *P. phanias* as indicated by both molecular and morphological data. The two species share massive subunci, a unique character within the genus, and similar valva, which are slender and bear several small teeth-like dorsal processes. However, *P. duartei* n. sp. is much larger than *P. phanias*, which is more obvious in the females, matching in size the other second largest member of *Praepedaliodes, P. granulata*. The two species, *P. granulata* and *P. duartei* n. sp., are locally sympatric.

**Fig 20:**
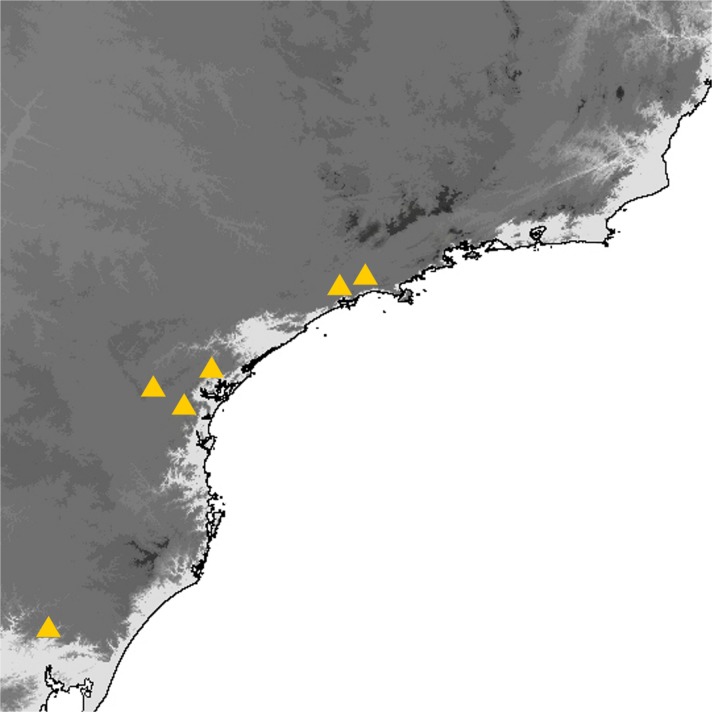
*Praepedaliodes granulata* distribution map.

**Fig 21:**
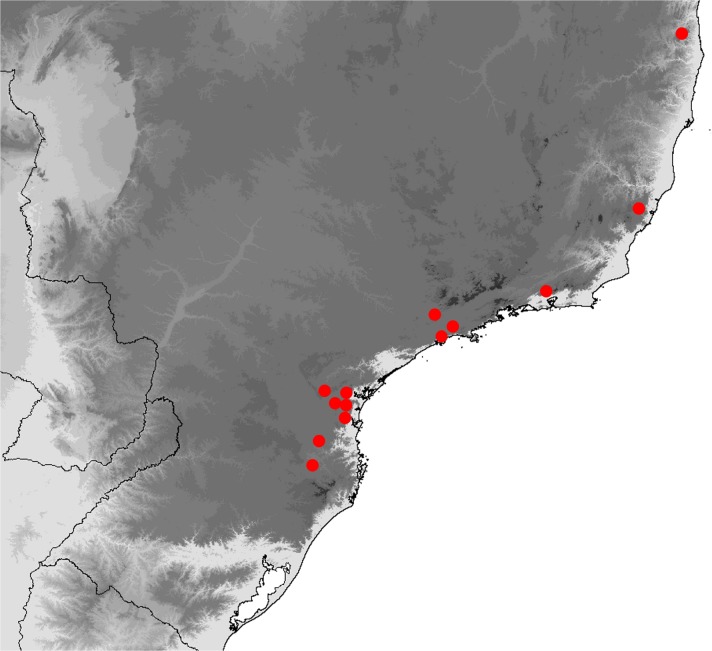
*Praepedaliodes duartei* n. sp. distribution map.

**Fig 22:**
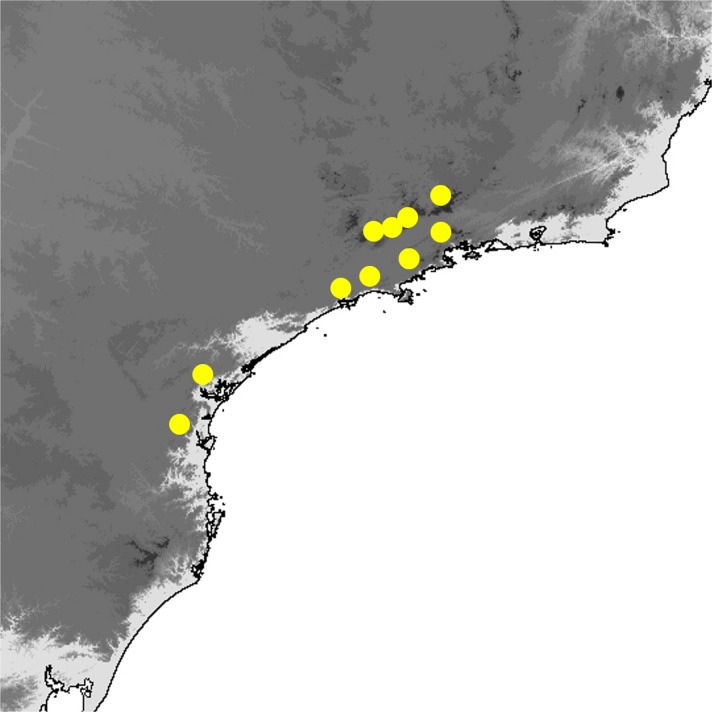
*Praepedaliodes amussis* distribution map.

***Praepedaliodes amussis*** (Thieme, 1905)

(Fig 1a, b, 5a, b, 10c, d, 16e, f and 22)

*Pedaliodes amussis* Thieme, 1905: 77, 78–79, pl. 3, fig 22; Weymer, 1912: 254, pl. 54, row a; Zikán, 1928: 8; Gaede, 1931: 488; D’Abrera, 1988: 851, fig [1]

*Muscopedaliodes amussis*; Forster, 1964: 154, 155, fig 186 (male genitalia)

*Panyapedaliodes amussis*; Adams, 1986: 276;

*Praepedaliodes amussis*; Lamas *et al* 2004: 214; Pyrcz, 2010: 242

Type locality: “Provinz Cauca (Centralcordillere von Colombia Gebirgsstock des Tolima)”, [Brazil]

Type material: *Pedaliodes amussis* Thieme, 1905 was described based on an unstated number of male and female specimens. One male syntype, deposited at the ZSBS, with the following labels is here designated lectotype to confirm the identity of the species / Kolumbia, Prov. Cauca, Tolima, Sammlung L. Martin / ex coll. Erhardt /. Characteristic lectotype labels will be added to the specimen.

### Material examined

BRAZIL: *Minas Gerais*: **Passa Quatro** (Fazenda dos Campos) 1 ♂ 12.X.1915, J. F. Zikán *leg.* (NHMUK), 1 ♂ 29.XI.1915, J. F. Zikán *leg.* (NHMUK), 1 ♂ 02.XII.1915, J. F. Zikán *leg.* (NHMUK), 1 ♂ 11.VIII.1916, J. F. Zikán *leg.* (NHMUK), 1 ♂ 18.II.1920, J. F. Zikán *leg.* (NHMUK). **Delfim Moreira** (15 km SE), 1500–1700 m, 1 ♀ 17-18.I.2004, Mielke & Casagrande *leg.* (DZ 36.394), 1 ♂ 22–23.I.2004, Mielke & Casagrande *leg.* (DZ 23.835) (DZUP). **Itamonte** (Serra do Itatiaia, N face), 1300 m, 2 ♂ XII.1957, H. Ebert *leg.*, ex coll. H. Ebert (DZ 35.791, DZ 36.414) (DZUP). *Rio de Janeiro*: **Itatiaia** (Parque Nacional do Itatiaia, South face), 1400 m, 1 ♂ 06.II.1958, H. Ebert *leg.*, ex coll. H. Ebert (DZ 36.397), 1500 m, 1 ♂ 14.IV.1965, H. Ebert *leg.*, ex coll. H. Ebert (DZ 36.400), 1200 m, 1 ♂ 04.V.1967, H. Ebert *leg.*, ex coll. H. Ebert (DZ 36.427), 1400 m, 3 ♂ 22.XII.1957, H. Ebert *leg.*, ex coll. H. Ebert (DZ 35.731, DZ 36.407, DZ 36.416), 1000–1400 m, 1 ♀ II.1960, H. Ebert *leg.*, ex coll. H. Ebert (DZ 36.426), 1600 m, 1 ♂ 10.II.1959, H. Ebert *leg.*, ex coll. H. Ebert (DZ 36.415), 1300 m, 1 ♂ 14.II.1959, H. Ebert *leg.*, ex coll. H. Ebert (DZ 36.405) (DZUP), 900 m, 1 ♂ and 1 ♀ 15.VII.1963, Mielke *leg.* (OM 5.222, OM 5.223) (OM). **Resende** 1 ♂ 12.II.1966, K. S. Brown Jr. *leg.* (ZUEC LEP 9949), 1 ♂ 21.I.1967, K. S. Brown Jr. *leg.* (ZUEC LEP 9950) (ZUEC). *São Paulo*: **Campos do Jordão**, 2000 m, 2 ♂ 08-12.II.1982, Mielke & Casagrande *leg.* (DZ 16.753, DZ 36.429 prep. genit. D. Dolibaina 2010) (DZUP), 1600–1700 m, 2 ♂ and 1 ♀ 22-25.I.1992, O. Mielke & M. Casagrande *leg.* (OM 28.527, OM 28.533, OM 28.521) (OM), (Umuarama), 1 ♂ 08-15.III.1937, P. Gagarin *leg.*, ex coll. H. Ebert (DZ 36.425) (DZUP), (Parque Estadual de Campos do Jordão) 1 ♂, 12.I.2001, A. V. L. Freitas & K. S. Brown Jr. *leg.* (ZUEC LEP 9951) (ZUEC). **Salesópolis** (Reserva Biológica de Boracéia), 1 ♀ 10.II.1942, ex coll. D’Almeida (DZ 35.821) (DZUP). **Santo André** (Vila de Paranapiacaba, Alto da Serra), 762 m, 1 ♂ 11-12.1919, A. Hall *leg.* (NHMUK). **São José do Barreiro** (Bananal, Serra da Bocaina), 2 ♂ and 1 ♀ 08.I.1937, Travassos *leg.*, ex coll. D’Almeida (DZ 35.801, DZ 36.424, DZ 36.417), (Serra da Bocaina), 1600 m, 1 ♂ 01.IV.2010, Melo *leg.* (DZ 23.895) (DZUP), 1 ♂ II.2014 (DNA voucher YPH 0446), T. W. Pyrcz *leg.* (ZUEC LEP 9952) (ZUEC). (Parque Nacional da Serra da Bocaina, 22°44′0″S 44°37′1″W), 1450 m, 3 ♂ 13.II.2014, P. Boyer *leg.* (MZUJ), (Parque Nacional da Serra da Bocaina, antenas 22°42′0″S 44°37′9″W) 1750 m, 1 ♂ 13.II.2014, P. Boyer *leg.* (MZUJ), (Parque Nacional da Serra da Bocaina, Trilha Principal), 1450–1500 m, 2 ♂ and 1 ♀ 13.II.2014, T. Pyrcz *leg.* (prep. genit. 03/01.04.2014 J. Lorenc) (MZUJ) [♂ Fig 1a, ♀ Fig 1b], (Parque Nacional da Serra da Bocaina, Trilha do Ouro), 1650–1700 m, 1 ♂ 14.II.2014, T. Pyrcz *leg.* (MZUJ), (Sertão da Bocaina), 1 ♀ 26.I.2015 (DNA voucher BLU 781), 1 ♀ 14.X.2015 (DNA voucher BLU 780), R. Raby *leg.* (ZUEC-AVLF). **Piquete** (Serra da Mantiqueira), 1300–1350 m, 1 ♂ and 3 ♀ 23.IV.2005, T. Pyrcz *leg.* (prep. genit. 01/09.11.2010 J. Lorenc, prep. genit. 05/24.08.2012 J. Lorenc, prep. genit. 04/09.05.2005 T. Pyrcz) (MZUJ). *Paraná*: **Morretes** (Alto da Serra), 800 m, 2 ♂ 12.IV.1988, Mielke *leg.* (OM 17.224, OM 17.225), 1 ♂ 25.II.1989, Mielke *leg.* (OM 20.956), 3 ♂ 17.III.1990, Mielke *leg.* (OM 25.141, OM 25.147, OM 25.159), 1 ♂ 08.V.1990, Mielke *leg.* (OM 25.863), 1 ♀ 01.V.1991, Mielke *leg.* (OM 25.787), 1 ♀ 27.II.1993, Mielke *leg.* (OM 34.456) (OM), (Serra da Graciosa, Rio Taquari), 850–900 m, 1 ♂ and 1 ♀ 01.II.2015, T. Pyrcz *leg.* (prep. genit. 303/16.12.2015 J. Lorenc, prep-molec no number (legs)) (MZUJ). **Piraquara** (Mananciais da Serra), 850 m, 1 ♂ 29.III.2007, Mielke & Casagrande *leg.* (DZ 23.384 prep. genit. D. Dolibaina 2010) (DZUP). **Tijucas do Sul** (Vossoroca), 850 m, 1 ♂ 08.II.1981, Mielke *leg.* (DZ 36.395), 1 ♂ 03.IV.1997, Mielke *leg.* (OM 45.601) (OM). *Santa Catarina*: **São Bento do Sul** (Rio Vermelho), 1 ♀ 27.III.1980, Rank *leg.* (DZ 36.430), 1 ♂ 17.XII.2015, Rank *leg.* (DZ 36.406) (DZUP). *Rio Grande do Sul*: **Porto Alegre**, 1 ♀, ex coll. A. Chaminade (PBF).

### Redescription

*Male* (Fig 1a, 16f). *Head*. Antennae reaching 2/5 the length of costa, slender, dorsally blackish brown, covered with black scales becoming sparser towards club, ventrally orange, mostly naked, except for a few basal flagellomeres with a few sparse snow white scales, club mostly naked, with 12 flagellomeres, slightly thicker than shaft, dorsally brown orange, ventrally light orange; eyes blackish brown, densely covered with short setae; labial palpi two times the length of head, covered mostly with blackish scales and hairy scales, considerably longer ventrally, except at the base and along lateral sides where covered with sandy yellow scales; collar with black elongated scales. *Thorax.* Dorsally and ventrally black, sparsely scaly, tegulae covered with brown and golden scales; legs black, femore covered with blackish scales, tibiae and tarsi with brown and, predominantly with sandy yellow scales. *Wings.* FW (length: 26–29 mm) with a subacute apex and straight outer margins, produced below apex; fringes dark brown at vein ends, sandy yellow scales in the interspaces; FWD lustrous, varying between uniform chestnut and dark brown; androconial patch not darker than the ground colour, compact, covering median one-fourth of wing surface, from discal cell distal edge to anal margin, entering discal cell. FWV taupe brown, with an overcast of chocolate brown in basal half and in the submarginal area; grey with sparse sandy yellow scaling along costal margin; black along distal margin; subapical, apical and marginal area from apex to vein CuA_2_ dusted with snow white scales; a row of five milky white postdiscal dots from R_5_-M_1_ to CuA_1_-CuA_2_. HWD uniform varying between chestnut and dark brown, lustrous, sparsely hairy in median half and along anal margin. HWV black, grey and snow white with a concentration of black in median area, and more prominent white scaling along outer and costal margin, forming two costal streaks, postbasal and median; a row of five to six submarginal milky white minute dots. *Abdomen.* Black, dorsally covered with dark brown, ventrally and laterally with brown and sandy yellow scales. *Male genitalia* (Fig 5a, b). Tegumen subtriangular in lateral view, dorsum flat, elongated vertically which is noticeable in the distance between subuncus base and pedunculus, same as tegumen dorsum length; uncus marginally shorter than tegumen dorsum, slender, ventrally slightly constricted near base, almost straight, with an acute tip; subunci slender, about the same width throughout, with sharp tip, two-thirds the length of uncus; pedunculus pointed, prominent; vinculum wide; saccus deep, the length of uncus, same width as vinculum in lateral view; valva slender, slightly shorter than the length of tegumen + uncus, almost the same width throughout, with a smooth dorsum except for a single prominent, acute subapical process pointing distally, tip elongated and sharp; aedeagus slender, marginally longer than length of saccus + valva, almost straight, with a sharp tip, smooth, spoon-like at base; proximal opening two-thirds the length of aedeagus.

*Female* (Fig 1b, 16e). Sexual dimorphism expressed in the lighter wing colours than of the male, similar size (FW length: 26–29 mm), dorsally chestnut with a darker distal margin; FWV with a light orange brown area extending from postdiscal to submarginal lines. HWV also considerably lighter with a white postdiscal-submarginal beige band, and a larger white mid-costal streak. Genitalia (Fig 10c, d). Compressed laterally in ventral view; papilla analis small, gently rounded in lateral view, covered with numerous and delicate setae; proximal unit, in lateral view, consisting of a weakly sclerotized, narrow lamella postvaginalis extending into two, moderately sclerotized, prominent lateral pocket-like folds with a several parallel incisions strongly compressed towards the entrance of ductus bursae; median unit with a slat-like, rather narrow but strongly sclerotized, lamella antevaginalis with smooth edges, merging ventro-laterally with lamella postvaginalis, enclosing from above the entrance to ductus bursae, with a shallow concavity; ductus bursae two-thirds the length of corpus bursae, tubular, strongly sclerotized, compressed in the middle, entrance of bursa slightly more sclerotized dorsally; ductus seminalis originating at the entrance of bursa; bursa copulatrix oval, with two wide signa extending over two-thirds of its length.

### Comments

The identity of *P. amussis* is not in doubt as this species is very characteristic and clearly illustrated by Thieme (1905). Viloria (unpublished data) indicates that the specimen curated in the ZSBS from the collection of Robert Erhardt is a syntype of *Pedaliodes amussis*. Even though the first choice of Thieme’s types and specimens of Neotropical Satyrinae were acquired by the MfN, most of his collection were sold to various collectors. Erhardt was an active collector at this time and his collection was later acquired by the ZSBS (Horn *et al.* 1935–1937); furthermore, Erhardt’s specimen and label data agrees perfectly with Thieme’s original description. Although unlikely, additional syntypes may be still found at the MfN, as suggested by Horn *et al.* (1935–1937). The incorrect type locality specified as Cauca in Colombia generated a great deal of systematic confusion. Forster (1964) included this species in his Andean genus *Muscopedaliodes* Forster, 1964, later synonymized with *Panyapedaliodes* by Adams (1986). Forster’s original decision was not only based on biogeographical grounds but also on a male genitalia comparative study. Indeed, the genitalia of *P. amussis* differ from all other species of *Praepedaliodes*. The uncus and subunci are slender, and the valvae are elongated with one prominent dorsal process. These, combined with the long aedeagus and the speckled HWV pattern, are the characters of the genus *Panyapedaliodes* as identified by Forster (1964) and refined by Adams (1986) (Fig 12e). The position of *P. amussis* in *Praepedaliodes* is upheld based on biogeographical and molecular evidence. *Praepedaliodes amussis* occurs in the Serra de Mantiqueira and the parallel Serra do Mar (Serra da Graciosa and Serra da Bocaina). It has not been reported, however, from the most northerly Serra do Caparaó, where other species also found in the Serra da Mantiqueira occur (Fig 22). This species is not rare, and most individuals were observed at 1400–1600 m. This species occurs syntopically with *P. francinii* Freitas & Pyrcz n. sp.

**Fig 23:**
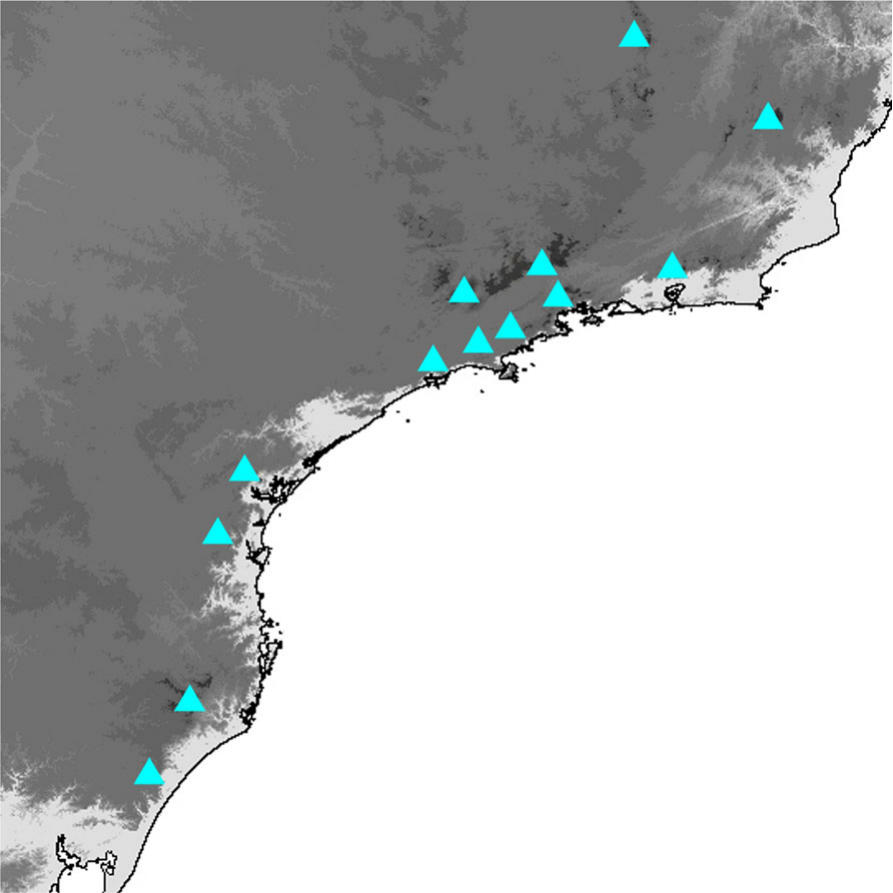
*Praepedaliodes landryi* n. sp. distribution map.

***Praepedaliodes landryi*** Pyrcz & Freitas **n. sp.**

(Figs 1e, f, 6a–c, 10g–h, 15a–p, 16b, c and 23)

Type locality: Parque Municipal Nascentes de Paranapiacaba, Serra de Paranapiacaba, Santo André, São Paulo, Brazil

### Type material

*Holotype* ♂ with the following labels: /Parque Municipal Nascentes de Paranapiacaba – Torres, Paranapiacaba, Santo André, São Paulo: Brazil, 1050–1150 m, 28.XI.2015 – A.V.L. Freitas *leg.*, 23°47′10″S 46°15′50″W, / DNA voucher BLU 784 / ZUEC LEP 9980 / Deposited in the Museu de Zoologia Adão José Cardoso, Universidade Estadual de Campinas, Campinas, São Paulo, Brazil (ZUEC).

*Paratypes.* (54 ♂ and 13 ♀): BRAZIL: *Minas Gerais*: **Alto Caparaó** (Serra do Caparaó, via parking), 1550–1600 m, 1 ♂ 06.II.2014, T. Pyrcz *leg.* (MZUJ), (Parque Nacional do Caparaó, S, 20°29′5″S 41°49′4″W) 1500 m, 1 ♂ 09.II.2014, P. Boyer *leg.* (MZUJ), 1 ♂ XII.2012 (DNA voucher BLU 373) (prep. genit. E. P. Barbosa 2013), 1 ♂ XII.2012, A. V. L. Freitas, L. A. Kaminski & C. A. Iserhard *leg.* (prep. genit. E. P. Barbosa 2013), (ZUEC LEP 9982, ZUEC LEP 9983), 1 ♀ 16.II.1969, K. S. Brown *leg.* (ZUEC LEP 9984) (ZUEC). **Delfim Moreira** (15 km SE), 1500-1700 m, 5 ♂ 17-18.I.2004, Mielke & Casagrande *leg.* (DZ 33.510, DZ 35.069, DZ 35.099, DZ 35.110, DZ 35.271), 5 ♂ 22-23.I.2004, Mielke & Casagrande *leg.* (DZ 19.242 prep. genit. D. Dolibaina 2010, DZ 23.504 prep. genit. F. Dias 2012, DZ 23.815 prep. genit. F. Dias 2012, DZ 23.595, DZ 34.965) (DZUP). *Espírito Santo*: **Dores do Rio Preto** (Pedra Menina, Serra do Caparaó, via refúgio), 1600–1800 m, 1 ♂ 06.II.2014, T. Pyrcz *leg.* (MZUJ). *Rio de Janeiro*: **Petrópolis** (Serra dos Órgãos), 1100 m, 1 ♀ 14.II.1979, H. Ebert *leg.*, ex coll. H. Ebert (DZ 35.811) (DZUP). *São Paulo*: 1 ♂, coll. Ch. J. Pitard (prep. genit. 11/24.03.2010 A. Zubek) (MNHG). **Campos do Jordão**, 1700 m, 1 ♂ 03.I.1966, H. Ebert *leg.*, ex coll. H. Ebert (DZ 35.721), 1650 m, 1 ♂ 18.IX.1966, H. Ebert *leg.*, ex coll. H. Ebert (DZ 33.317), (S22°46′1 W45°36′8) (DZUP), 1500 m, 2 ♂ 20.II.2014, P. Boyer *leg.* (MZUJ), (Alto Capivari), 1700–1750 m, 2 ♂ 04.II.2014, T. Pyrcz *leg.* (prep. genit. 02/03.04.2014 J. Lorenc) (MZUJ), (Trilha do Zigue-Zague), 1400–1450 m, 1 ♂ 28.IV.2012, T. Pyrcz *leg.* (prep. genit. 01/20.05.2012 J. Lorenc) (MZUJ), (Umuarama), 1800 m, 1 ♂ 03-15.II.1937, P. Gagarin *leg.*, ex coll. H. Ebert (DZ 20.148 prep. genit. D. Dolibaina 2010), (Parque Estadual de Campos do Jordão), 1 ♂ 11.II.1968, K. S. Brown Jr. *leg.* (ZUEC LEP 9981) (ZUEC), (Alto da Boa Vista), 1820 m, 1 ♂ 1 ♀ 3-4.II.2014 (DNA voucher YPH 0445, YPH 0443), B-882/CJABV, A. V. L. Freitas *leg.* (ZUEC-AVLF). **Pindamonhangaba** (Pico do Itapeva), 1850 m, 1 ♂ and 1 ♀ 28.I.1967, A. Barroso & R. Travassos *leg.* (MZUSP), 1850–1900 m, 2 ♂ and 1 ♀ 11.IV.2012, T. Pyrcz *leg.* (prep. genit. 02/11.04.2012 J. Lorenc, prep. genit. 05/11.04.2012 J. Lorenc) (MZUJ), 1 ♂ 27.IV.2012, T. Pyrcz *leg.* (MZUJ), (forest trail), 3 ♂ 03.II.2014, T. Pyrcz *leg.* (prep. genit. 02/24.02.2015 J. Lorenc) (MZUJ). **Salesópolis** (Serra da Bocaina), 1550 m, 1 ♂ 02-04.III.1967, H. Ebert *leg.*, ex coll. H. Ebert (DZ 35.089). **Santo André** (Parque das Nascentes de Paranapiacaba), 1100–1200 m, 2 ♂ 28.XI.2015 (DNA vouchers BLU 782, BLU 783), A. V. L. Freitas *leg.* (ZUEC LEP 9978, ZUEC LEP 9979) (ZUEC), 1 ♀ 23.XII.2013 (DNA voucher YPH 0369), B-875/TORRE, A. V. L. Freitas *leg.* (ZUEC-AVLF). **São José do Barreiro** (Parque Nacional da Serra da Bocaina, Trilha Principal), 1450–1500 m, 1 ♂ 13.II.2014, T. Pyrcz *leg.* (prep. genit. 01/03.04.2014 J. Lorenc) (MZUJ). *Paraná*: **Morretes** (Alto da Serra), 800 m, 2 ♂ 12.IV.1988, Mielke *leg.* (OM 17.223, OM 17.226), 1 ♂ 04.II.1989, Mielke *leg.* (OM 21.712), 1 ♂ 28.II.1989, Mielke *leg.* (OM 20.750), 1 ♂ and 1 ♀ 08.II.1990, Mielke *leg.* (OM 25.001, OM 25.007), 2 ♂ 28.I.1993, Mielke *leg.* (OM 33.715, OM 33.853) (OM), 1 ♀ 22.II.1997, 850 m, A. Moser leg. (CLAM), (Serra da Graciosa, Casa de Pedra), 900–920 m, 1 ♂ 31.I.2015, T. Pyrcz *leg.* (prep. genit. 307/16.12.2015 J. Lorenc) (MZUJ), 1 ♂ and 1 ♀ 01.II.2015, T. Pyrcz *leg.* (prep. genit. 304/16.12.2015 J. Lorenc) (MZUJ), (Rio Taquari, 25°19′S 48°56′W), 850–900 m, 1 ♂ 01.II.2015, P. Boyer *leg.* (MZUJ). *Santa Catarina*: **Joinville** (Serra Dona Francisca), 770–800 m, 1 ♂ and 2 ♀ 19.II.2015, T. Pyrcz *leg.* (prep. genit. 305/16.12.2015 J. Lorenc) (MZUJ) [♀ Fig 1f]. **Urubici** (Morro da Igreja), 1400 m, 1 ♀ 13-14.I.1998, Mielke *leg.* (OM 48.495), 1 ♂ 22.II.2002, Mielke & Casagrande *leg.* (OM 56.139) (OM), (SC-370 road to Gravatal, 28°03′49″S 49°22′20″W), 1250–1300 m, 1 ♂ 11.II.2015, P. Boyer *leg.* (MZUJ), (Serra do Corvo Branco), 1200–1300 m, 2 ♂ 11.II.2015, T. Pyrcz *leg.* (prep. genit. 12/13.03.2015 J. Lorenc) (MZUJ) [♂ Fig 1e], 1 ♂ 11.II.2015 (DNA voucher BLU 720), T. W. Pyrcz *leg.* (ZUEC LEP 9985) (ZUEC). **São Bento do Sul** (road to Joinville, Campo Alegre, 1 ♀ 19.II.2015 (DNA voucher BLU 721), T. W. Pyrcz *leg.* (ZUEC LEP 9986) (ZUEC). *Rio Grande do Sul*: **Itaimbezinho** (Reserva Araucária), 1 ♂ IX.1979, ex coll. Gifford (DZ 16.774 gen. prep. D. Dolibaina 2010) (DZUP).

### Diagnosis

All brown upperside as in other sympatric congeners; differs from *P. pawlaki* Pyrcz & Boyer n. sp. and *P. francinii* Freitas & Pyrcz n. sp. in the produced outer margin below apex; however, most notably it differs from other congeners in the HWV pattern, particularly in the chocolate brown ground colour, and the well-defined, wide, darker median band.

### Description

*Male* (Fig 1e). *Head*: Antennae reaching 2/5 the length of costa, slender, dark brown, ventrally orange, naked, except for a few basal flagellomere covered with sparse milky white scales, club naked, with 12 flagellomeres, slightly thicker than shaft, ventrally orange; eyes chocolate brown, densely setose; labial palpi two times the length of head, covered with brown sandy yellow hairy scales, with some yellow scales dorsally; collar with brown and golden elongated scales. *Thorax.* Dorsally and ventrally black, sparsely scaly, tegulae covered with brown and golden scales; legs brown, tibiae and tarsi covered with brown and sandy yellow scales. *Wings.* FW (length: 24–27 mm) with a subacute apex and outer margins straight but produced below apex; fringes intermittently brown and sandy yellow; FWD uniform seal brown, lustrous; androconial patch a shade darker, covering median one-fourth of wing surface, from discal cell distal edge to anal margin, marginally entering discal cell. FWV taupe brown, lighter and duller than on the dorsal surface; a shade darker median patch corresponding to the area covered by the androconial patch on the dorsum; a faint, sinuate dark brown submarginal line; in some individuals a minute yellowish subapical dot; HW oval with a gently undulating outer margin; fringes intermittently brown and sandy yellow; HWD uniform taupe brown, lustrous, sparsely hairy in median half and along anal margin. HWV chocolate brown with a darker basal and postbasal area, a wide median band with an irregular, outer edge, and a marginal area; a series of minute yellowish submarginal dots. *Abdomen.* Dorsally covered with dark brown, ventrally and laterally with taupe brown scales. Genitalia (Fig 6a–c). Tegumen massive, triangular in lateral view, dorsum arched; uncus the length of tegumen dorsum, slender, ventrally strongly constricted near base, nearly straight, with a sharp tip; subunci stout, wide at base and strongly adhered to tegumen, gradually narrowing towards a sharp tip, three-fourths the length of uncus, gently lifted upwards; pedunculus short, wide ended with a sharp tip; vinculum slender; saccus moderately deep, the length of subunci, two times as wide as vinculum in lateral view; valva slender, marginally shorter than tegumen + uncus, only slightly wider at base than towards apex, with a series of sharp, spiny processes along distal half of dorsal surface, of roughly similar same size, quite variable among specimens, with the subapical process generally slightly longer than the remaining ones, apex blunt; aedeagus slender, marginally shorter than saccus + valva, gently bent in the middle, with a sharp tip, smooth, spoon-like at base; proximal opening half the length of aedeagus.

*Female* (Fig 1f, 16b, c). The female is noticeably larger than the male (FW length: 27–30 mm) and its wings are differently shaped, wider and give the impression of being rounder, which is due especially to the convex FW outer margin. Wings colour patterns are similar, although generally the HWV pattern is slightly more contrasting. Genitalia (Fig 10g, h). Flattened laterally in ventral view; papilla analis prominent, gently rounded in lateral view, covered with sparse, but rather stout and long setae; proximal unit, in lateral view, consisting of a weakly sclerotized narrow lamella postvaginalis transforming gradually into two, moderately sclerotized, massive lateral pocket-like folds with a strongly rippled surface, compressed towards the entrance of ductus bursae; median unit with a slat-like, wide and strongly sclerotized lamella antevaginalis with smooth edges, touching but not merging ventro-laterally with lamella postvaginalis, enclosing from above the entrance to ductus bursae, with a deep incision and a shallow concavity; ductus bursae two-thirds the length of corpus bursae, tubular, sclerotized (but less so than in other congeners), wide and approximately the same width throughout, entrance of bursa with a sclerotized bulb in ventral position; ductus seminalis originating at the entrance of bursa; bursa copulatrix rounded, with two wide signa extending over two-thirds of its length.

### Immature stages

The following descriptions and measurements are based on material reared from one female from Paranapiacaba, Santo André, São Paulo. Additional material from the same place and from Campos do Jordão, São Paulo, gave similar results.

*Egg* (Fig 15a, b). Spherical, light yellowish, smooth, with a fine ornamentation of small concavities barely visible under microscope. Height 1.20–1.24 mm (mean = 1.21 mm; SD = 0.018 mm; *n* = 5); diameter 1.20–1.22 mm (mean = 1.20 mm; SD = 0.009 mm; *n* = 5). Duration 7–8 days (*n* = 12).

*First instar* (Fig 15c, d). Head capsule width 0.80–0.84 mm (mean = 0.82 mm; SD = 0.023 mm; *n* = 6); head scoli 0.06–0.10 mm (mean = 0.07 mm; SD = 0.015 mm; *n* = 6). Head capsule dark brown, with enlarged chalazae, bearing a pair of short light brown scoli on vertex, each with two long narrow setae. Third stemma larger than the other stemmata. Body light green, greenish dorsally, smooth, with a white longitudinal subdorsal stripe; caudal filaments very short. Setae light brown and elongated. Legs and prolegs light. Maximum length 6 mm. Duration 5–6 days (*n* = 20).

*Second instar* (Fig 15e, f). Head capsule width 1.06–1.20 mm (mean = 1.12 mm; SD = 0.050 mm; *n* = 6); head scoli 0.26–0.34 mm (mean = 0.30 mm; SD = 0.032 mm; *n* = 6). Head brown with two diverging short scoli light brown scoli on vertex. Body brown, striped longitudinally with white and light brown; caudal filaments short. Legs and prolegs light. Maximum length 11 mm. Duration 5 days (*n* = 15).

*Third instar* (Fig 15g, h). Head capsule width 1.60–1.80 mm (mean = 1.69 mm; SD = 0.091 mm; *n* = 6); head scoli 0.44–0.52 mm (mean = 0.47 mm; SD = 0.035 mm; *n* = 6). Head dark brown, light on vertex, with two diverging light brown short scoli on vertex. Body light brown, laterally striped with dark brown and white, greenish on dorsal thoracic segments, with a conspicuous subdorsal zig-zag pattern from A1 to A6; caudal filaments short. Legs and prolegs light. Maximum length 15 mm. Duration 7–8 days (*n* = 15).

*Fourth instar* (Fig 15i, j). Head capsule width 2.20–2.44 mm (mean = 2.38 mm; SD = 0.110 mm; *n* = 6); head scoli 0.60–0.70 mm (mean = 0.64 mm; SD = 0.034 mm; *n* = 6). Head dark brown, light brown on vertex, with two diverging light brown short scoli on vertex. Body light brown, laterally striped with dark brown and white, greenish on dorsal thoracic segments, with a conspicuous subdorsal zig-zag pattern from A1 to A6; caudal filaments short. Legs and prolegs light. Maximum length 22 mm. Duration 8 days (*n* = 12).

*Fifth (last) instar* (Fig 15k, l). Head capsule width 3.39–3.64 mm (mean = 3.48 mm; SD = 0.116 mm; *n* = 4); head scoli 0.92–1.05 mm (mean = 0.96 mm; SD = 0.062 mm; *n* = 4). Head dark brown, slightly lighter on vertex, with two diverging dark brown short scoli on vertex. Body dark brown, with a discreet subdorsal zig-zag pattern with green patches from A1 to A6; caudal filaments short. Legs and prolegs brown. Maximum length 37 mm. Duration 9–11 days (*n* = 10).

*Pupa* (Fig 15m–o). Short; mostly brown; short pointed diverging ocular; cremaster brown; dorsal abdomen with paired pointed projections from A1 to A7. Total length 16–17 mm (*n* = 6). Duration 12–13 days (*n* = 5).

### Etymology

This species is dedicated to the Canadian entomologist, Dr. Bernard Landry, currently the curator of Lepidoptera at the Muséum d’Histoire Naturelle de la Ville de Genève, and specialist in the taxonomy of several families of Microlepidoptera, and the fauna of the Islands of Galápagos.

### Comments

*Praepedaliodes landryi* Pyrcz & Freitas n. sp. is another widely distributed member of the genus. It is found in the northern Caparaó range, Serra da Mantiqueira and in several localities of the Serra do Mar, including the most southerly known locality for the genus, except for *P. phanias*, in São Francisco de Paula (Rio Grande do Sul) (Fig 23). In the north, it flies at higher elevations (1400–1600 m) than in the south (900–1300 m). It is more seldom encountered in the field than some of its congeners, such as *P. francinii* n. sp. or *P. pawlaki* n. sp., but it is especially common in the type locality (Paranapiacaba, Santo André, São Paulo). However, it is more tolerant of disturbed areas and occasionally can be seen flying over pastures and forest clearings, which are avoided by other species of *Praepedaliodes* excluding *P. phanias*.

**Fig 24:**
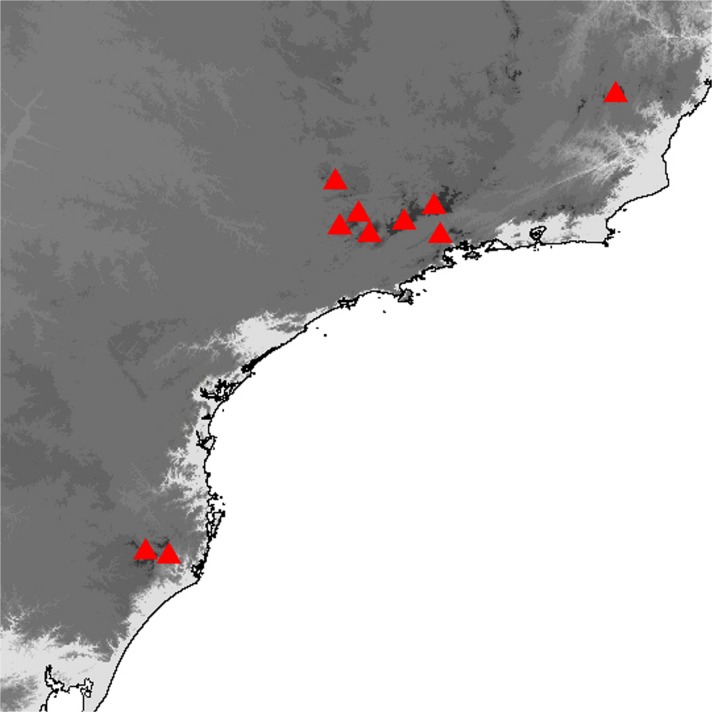
*Praepedaliodes francinii* n. sp. distribution map.

***Praepedaliodes francinii*** Freitas & Pyrcz n. sp.

(Figs 1c, d, 6d–f, 10e, f and 24)

Type locality: Barreira do Piquete, Piquete, São Paulo, Brazil

### Type material

Holotype ♂ with the following labels: /15.II.1984, Barreira do Piquete, [Piquete,] S[ão]. Paulo 1400–1600 m, Mielke & Casagrande *leg.*/ DZ 16.176/ HOLOTYPUS/ HOLOTYPE *Praepedaliodes francinii* Freitas & Pyrcz, det 2016/ (prep. genit. D. Dolibaina 2010). Deposited in the Coleção Entomológica Padre Jesus Santiago Moure, Departamento de Zoologia, Universidade Federal do Paraná, Curitiba, Paraná, Brazil (DZUP).

Allotype ♀ with the following labels: /15.II.1984, Barreira do Piquete, [Piquete,] S[ão]. Paulo 1400–1600 m, Mielke & Casagrande *leg.*/ DZ 23.875/ ALLOTYPUS/ ALLOTYPE *Praepedaliodes francinii* Freitas & Pyrcz, det 2016/ (prep. genit. D. Dolibaina 2010). Deposited in the Coleção Entomológica Padre Jesus Santiago Moure, Departamento de Zoologia, Universidade Federal do Paraná, Curitiba, Paraná, Brazil (DZUP).

*Paratypes*. (113 ♂ and 19 ♀): BRAZIL: *Minas Gerais*: **Passa Quatro** (Fazenda dos Campos) 1 ♂ 13.VIII.1918, J. F. Zikán *leg.* (NHMUK), 1 ♂ 29.IX.1921 (NHMUK). **Alto Caparaó** (Parque Nacional do Caparaó, 20°24′4″S 41°50′8″W), 1750 m, 2 ♂ 08.II.2014, P. Boyer *leg.* (MZUJ), (Serra do Caparaó, via parking), 1550–1600 m, 7 ♂ 08.II.2014, T. Pyrcz *leg.* (prep. genit. 04/03.04.2014 J. Lorenc) (MZUJ), 3 ♂ XII.2012 (DNA vouchers BLU 369, BLU 370, BLU 371) (prep. genit. E. P. Barbosa 2013), 1 ♂ XII.2012, A. V. L. Freitas, L. A. Kaminski & C. A. Iserhard *leg.* (ZUEC LEP 9992, ZUEC LEP 9993, ZUEC LEP 9994, ZUEC LEP 9995), 2100 m, 1 ♂ (prep. genit. – slide no. 1729 – Lee D. Miller), 16.II.1969, 1 ♂ (prep. genit. – slide no. 1732 – Lee D. Miller), 10.II.1968 (incorrect), K. S. Brown Jr. *leg.* (ZUEC LEP 9996, ZUEC LEP 9997), 1 ♀ 15.II.1969, 1 ♀ 16.II.1969, K. S. Brown Jr. *leg.* (ZUEC LEP 9998, ZUEC LEP 9999) (ZUEC). **Camanducaia** (Monteverde), 1650 m, 1 ♂ 22.XII.1968, H. Ebert *leg.*, ex coll. H. Ebert (DZ 36.422), 1 ♀ 08.III.1971, H. Ebert *leg.*, ex coll. H. Ebert (DZ 35.075) (DZUP). **Extrema** (Serra do Lopo, 22°53′26″S 46°18′94″W), 1550–1650 m,1 ♂ 21.II.2015, P. Boyer *leg.* (MZUJ), 3 ♂ and 1 ♀ same data as above, T. Pyrcz *leg.* (MZUJ). **Itamonte** (Serra do Itatiaia, N face), 1600 m, 1 ♀ 21.II.1959, H. Ebert *leg.*, ex coll. H. Ebert (DZ 36.615) (DZUP). **Poços de Caldas**, 1250 m, 1 ♂ 28.V.1962, H. Ebert *leg.*, ex coll. H. Ebert (DZ 35.160), 1 ♂ and 2 ♀ 01-05.V.1963, H. Ebert *leg.*, ex coll. H. Ebert (DZ 36.402, DZ 36.399, DZ 36.419), 1 ♂ 12.IX.1964, H. Ebert *leg.*, ex coll. H. Ebert (DZ 36.409), 2 ♂ 23.IV.1967, H. Ebert *leg.*, ex coll. H. Ebert (DZ 23.324 prep. genit. D. Dolibaina 2010, DZ 32.344 prep. genit. D. Dolibaina 2010) (DZUP). *Espírito Santo*: **Dores do Rio Preto** (Parque Nacional do Caparaó, S region), 1750 m, 1 ♂ 09.II.2014, P. Boyer *leg.* (MZUJ). *Rio de Janeiro*: **Itatiaia** (Parque Nacional do Itatiaia, South face), 1 ♂ X.1931, B. Pohl *leg.* (MZUSP), 1900 m, 1 ♂ 23.III.1927, ex coll. H. Ebert (DZ 36.655), 1400 m, 1 ♀ 22.XII.1957, ex coll. H. Ebert (DZ 36.615), 1500 m, 1 ♂ 14.IV.1965, H. Ebert *leg.*, ex coll. H. Ebert (DZ 36.625), 1900 m, 3 ♂ and 1 ♀ 27.II.1964, H. Ebert *leg.*, ex coll. H. Ebert (DZ 36.685, DZ 36.715, DZ 36.735, DZ 36.555), 1 ♂ 19.III.1971, H. Ebert *leg.*, ex coll. H. Ebert (DZ 36.905 prep. genit. F. Dias 2016) (DZUP), (Parque Nacional do Itatiaia, km1 da estrada para Agulhas Negras), 1700 m,1 ♂ 06.II.2014, P. Boyer *leg.* (MZUJ), (Parque Nacional do Itatiaia, estrada para Agulhas Negras, prepáramo), 2300–2350 m, 1 ♀ 05.II.2014, T. Pyrcz *leg.* (prep. genit. 03/24.02.2015) (MZUJ). *São Paulo*: **Campos do Jordão**, 1700 m, 5 ♂ I.1966, H. Ebert *leg.*, ex coll. H. Ebert (1 ♂ DZ 27.505 prep. genit. F.M.S. Dias 2012) (DZ 27.505, DZ 35.150, DZ 35.851, DZ 36.401, DZ 36.421), 1 ♂ 11.XI.1966, H. Ebert *leg.*, ex coll. H. Ebert (DZ 36.398) (DZUP), (S22°46′1 W045°36′8), 1500 m, 1 ♂ 02.II.2014, P. Boyer *leg.* (MZUJ), 1 ♂ 03-04.II.2014, P. Boyer *leg.*, (MZUJ), 1 ♂ 20.II.2014, P. Boyer *leg.* (MZUJ), (Trilha do zigue-zague), 1400–1450 m, 1 ♂ 02.II.2014, T. Pyrcz *leg.* (MZUJ), (Travessia Pinda Forest), 1550–1600 m, 1 ♂ 03.II.2014, T. Pyrcz *leg.* (MZUJ), (Umuarama) 1800 m, 5 ♂ 03-15.II.1937, P. Gagarin *leg.*, ex coll. Ebert (DZ 36.408, DZ 36.418, DZ 36.428, DZ 36.431, DZ 36.432), (Parque Estadual de Campos do Jordão), 1 ♂ 10.II.1968, K. S. Brown Jr. *leg.* (ZUEC LEP 9991), 3 ♂ 04.II.2014 (DNA vouchers BLU 623, BLU 625, BLU 627), A. V. L. Freitas *leg.* (ZUEC LEP 9987, ZUEC LEP 9988, ZUEC LEP 9989), (Alto da Boa Vista), 1 ♂ 03.II.2014 (DNA voucher BLU 626), A. V. L. Freitas *leg.* (ZUEC LEP 9990) (ZUEC), (Alto Capivari), 1790 m, 1 ♂ 17-20.I.2014 (DNA vouchers YPH 0398), B-870/CJACP, A. V. L. Freitas *leg.* (ZUEC-AVLF), 1 ♂ VII.1977, 2000 m, A. Moser *leg.* (CLAM). **Pindamonhangaba** (Pico do Itapeva), 1850–1900 m, 6 ♂ and 2 ♀ 11.IV.2012, T. Pyrcz *leg.* (prep. genit. 07/20.05.2012 J. Lorenc) (MZUJ), 4 ♂ and 2 ♀ 13.IV.2012, T. Pyrcz *leg.* (prep. genit. 05/20.05.2012 J. Lorenc) (MZUJ), 2 ♂ 27.IV.2012, T. Pyrcz *leg.* (MZUJ), 2 ♂ 20.II.2014, T. Pyrcz *leg.* (MZUJ), (Pico do Itapeva, Forest trail), 1850–1900 m, 2 ♂ 03.II.2014, T. Pyrcz *leg.* (MZUJ), (Pico do Itapeva, lago grassland), 1800–1850 m, 1 ♀ 02.II.2014, T. Pyrcz *leg.* (MZUJ). **Piquete** (Serra da Mantiqueira), 1450–1500 m, 1 ♂ 23.IV.2005, T. Pyrcz *leg.* (MZUJ). **São Bento do Sapucaí** (Campos do Serrano), 1100 m, 1 ♂ 11.V.1964, H. Ebert *leg.*, ex coll. H. Ebert (DZ 27.335), 1600 m, 1 ♂ 11.V.1964, H. Ebert *leg.*, ex coll. H. Ebert (gen. prep. F.M.S. Dias 2012) (DZ 35.120) (DZUP). **São José do Barreiro** (Parque Nacional da Serra da Bocaina, trilha do ouro), 1650–1700 m, 1 ♂ 14.II.2014, T. Pyrcz *leg.* (DNA voucher YPH 0448) (MZUJ), (Parque Nacional da Serra da Bocaina, trilha principal), 1450–1500 m, 1 ♂ 13.II.2014, T. Pyrcz *leg.* (MZUJ), (Serra da Bocaina), 1750 m, 1 ♀ 5.III.1966, H. Ebert *leg.*, ex coll. H. Ebert (DZ 36.411) (DZUP). *Santa Catarina*: **Urubici** (Morro da Igreja), 1400 m, 1 ♂ 13-14.I.1998, Mielke *leg.* (OM 48.527) (OM), (Mundo Novo, 10 Km S of Urubici, 28°04′18″S 49°36′53″W), 1450–1470 m, 3 ♂ 09.II.2015, P. Boyer *leg.* (MZUJ), same data as above 2 ♂ and 1♀ T. Pyrcz *leg.* (prep. genit. 299/16.12.2015 J. Lorenc) (MZUJ) [♀ Fig 1d], 1 ♀ 10.II.2015, P. Boyer *leg.* (MZUJ), (Serra do Corvo Branco, 28°03′49″S 49°22′20″W), 1180 m, 3 ♂ 11.II.2015, A. Moser *leg.* (MZUJ), 1200–1300 m, 11 ♂ and 1 ♀ 11.II.2015, T. Pyrcz *leg.* (prep. genit. 300/16.12.2015 J. Lorenc, prep. genit. 301/16.12.2015 J. Lorenc, prep. genit. 05/10.06.2015 J. Lorenc) (MZUJ) [♂ Fig 1c], 10 ♂ 11.II.2015, P. Boyer *leg.* (MZUJ), 1500 m, 1 ♂ 09.II.2015 (DNA voucher BLU 719), T. W. Pyrcz *leg.* (ZUEC LEP 1001) (ZUEC). *No data*: 1 ♂ 23.IV.1934 (MZUSP).

### Diagnosis

Wings upperside all brown, as in other sympatric congeners; it differs from the most similar *P. pawlaki* n. sp. in the straight FW outer margin and the subacute apex, slightly truncate below apex in *P. pawlaki* n. sp., which gives it the impression of a shallow concavity; it most notably differs from other congeners by the darker and more uniform HWV colour, compared to *P. landryi* Pyrcz & Freitas n. sp., and by the diagnostic HWV silver speckling and no clearly defined postdiscal line, the latter deeply incurved basally in space CuA_1_-CuA_2_ in *P. pawlaki* n. sp., and forming a sharp notch along M_3_ in *P. landryi* n. sp.

### Description

*Male* (Fig 1c). Head: Antennae reaching 2/5 the length of costa, slender, dorsally dark brown, ventrally orange, with sparse milky white scales at the base of each flagellomere, club naked, with 11 flagellomeres, slightly thicker than shaft; eyes chocolate brown, densely setose; labial palpi two times the length of head, covered with blackish hairy scales and laterally with some milky white scales; collar with taupe brown elongated scales. *Thorax*. dorsally and ventrally black, densely scaly, tegulae covered with taupe brown scales; legs black, tibiae and tarsi covered with sandy yellow scales. *Wings.* FW (length: 24–27 mm) with a subacute apex and straight outer margin, the impression of slight undulation produced by the intermittently brown and milky white fringes; FWD uniform taupe brown, lustrous; androconial patch a shade darker, covering median one-third of wing surface, from subapical area to anal margin, entering discal cell. FWV taupe brown, a shade lighter and duller than on the dorsal surface; a shade darker median patch corresponding to the area covered by the androconial patch on the dorsum; a few silver scales scattered on the apical area and along distal margin; marginal area a shade darker. HW rounded with a gently scalloped outer margin; fringes milky white from apex to vein M_3_, then brown to tornus. HWD uniform taupe brown, lustrous, slightly hairy in basal and postbasal area. HWV blackish brown with silver scales, liberal, sparse dotting of variable expression in different individuals, not forming any concentration or pattern except for a short mid-costal streak, in some individuals faint, and a row of slightly more noticeable submarginal dots; in some individuals the postdiscal - submarginal area a shade lighter. *Abdomen.* dorsally black, ventrally and laterally with grey scales. *Genitalia* (Fig 6d–f). Tegumen massive with a flat dorsal surface; uncus three-fifths the length of tegumen dorsum, with a small basal ventral constriction, bent downwards in the middle, with a slightly uplifted, sharp tip; subunci massive, two-thirds the length of uncus, with a subacute tip; pedunculus moderately long; vinculum short, saccus straight and long, approximately the length of tegumen dorsum; valva slender, gradually narrowing towards apex, the length of tegumen + uncus, with a series of 5–7 sharp, spiny dorsal processes of variable length, the most prominent of which being invariably the subapical one, two times as long the second longest; aedeagus the length of valve + half the length of saccus, nearly straight and slender, with the proximal opening almost half its length, and a spiny apical crest.

*Female* (Fig 1d). Sexual dimorphism is slight, size is nearly similar (FW length: 24–28 mm). The female differs in the slightly more rounded, and wider wings, a more scalloped HW outer margin, and the lighter, medium brown colour of both the dorsal and ventral wing surface, particularly on the HWV postdiscal area, which is dusted with even lighter, yellowish scales; and in the lack of the diagnostic to the male silver speckling of the HWV. *Genitalia* (Fig 10e, f). Strongly flattened laterally in ventral view; papilla analis prominent, gently rounded in lateral view, covered with dense setae, some of which are considerably longer than the others; proximal unit, in lateral view, consisting of a weakly sclerotized lamella postvaginalis transforming gradually into two, strongly sclerotized, massive lateral pocket-like folds with a heavily rippled surface, slightly compressed towards the entrance of ductus bursae; median unit with a slat-like, strongly sclerotized, wide lamella antegavinalis with smooth edges, touching but not merging ventro-laterally with lamella postvaginalis, enclosing from above the entrance to ductus bursae, with a narrow and deep incision and a shallow, barely marked concavity; ductus bursae three-fifths the length of corpus bursae, tubular, strongly sclerotized, wide and slightly compressed in the middle, entrance of bursa with a strongly sclerotized bulb in ventral position; ductus seminalis originating at the entrance of bursa; bursa copulatrix oval, with two wide signa extending over three-fifths of its length.

### Etymology

Dedicated to the Brazilian lepidopterist, Dr. Ronaldo Bastos Francini, from Santos, São Paulo State, Brazil, whose major contribution has been in the taxonomy, ecology and biology of Neotropical Acraeini (Nymphalidae). Dr. Francini was the first scientific advisor of the second author (AVLF) and had strong influence in his biological career.

### Comments

This species has a rather large distribution. It is found in all three widely separated high elevation mountain ranges of south and southeastern Brazil, Caparaó, Mantiqueira and Serra do Mar (Fig 24). The specimens found in the south part of Serra do Mar (Serra Geral) are darkest and least patterned with pale markings but otherwise there is little morphological and genetic evidence that would support their subspecific status. *Praepedaliodes francinii* n. sp. occurs at mid-elevations, and slightly higher in altitude in the north of the range (1500–1800 m) than in the south (1300–1500 m). It is generally among the most common species of the genus. It is associated with both primary and secondary cloud forests and can be observed wherever species of *Chusquea* bamboo is abundant.

**Fig 25:**
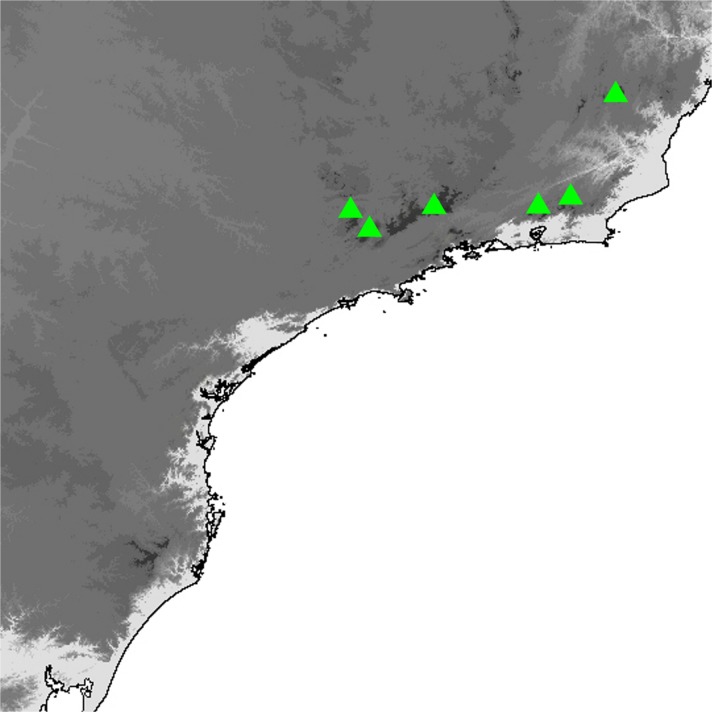
*Praepedaliodes exul* distribution map.

***Praepedaliodes exul*** (Thieme, 1905)

(Figs 3a, b, 7a, b, 9g, h, 16d and 25)

*Pedaliodes exul* Thieme, 1905: 59, 63–64, pl. 3, fig 32; D’Abrera, 1988: 850, fig [6]

*Pedaliodes poetica* form *exul*; Weymer, 1912: 252, pl. 53, row e

*Pedaliodes poetica exul*; Zikán, 1928: 8

*Pedaliodes poetica* var. *exul*; Gaede, 1931: 500

*Praepedaliodes exul*; Lamas *et al* 2004: 214; Pyrcz, 2010: 242

Type locality: Rio de Janeiro, Brazil

### Type material

*Pedaliodes exul* Thieme, 1905 was described based on three males and one female from Rio de Janeiro, Brazil, from Staudinger’s collection, currently in the MfN. One syntype, illustrated by Warren *et al* (2016), with the following labels, is here designated lectotype to confirm the identity of the species: /Prov[ince of]. Rio [de Janeiro, Brazil], ‘[18]95, Fött[erle]/ Lectotype ♂ *Pedaliodes exul* Thieme designated by Lee D. Miller, 1989/ genit. vial 9008 ♂ Lee D. Miller/. Characteristic labels will be added to the specimen; the remaining syntypes are designated paralectotypes and will be labelled accordingly. Lectotype and paralectotypes designations by Lee D. Miller were never published, and therefore, are invalid.

### Material examined

BRAZIL: *Minas Gerais*: **Alto Caparaó** (Parque Nacional do Caparaó), 1900 m, 1 ♂ 6-8.II.1987, Mielke & Casagrande *leg.* (OM 13.379), 2000 m, 1 ♂ (OM 13.751), 2000–2100 m, 1 ♀ (OM 13.747), 2000–2200 m, 1 ♂ (OM 13.749), 2000–2500 m, 1 ♂ (OM 13.746), 2500–2700 m, 1 ♂ (OM 13.381) (all with the same date and collectors as above) (OM), (Parque Nacional do Caparaó, 20°24′8″S 041°49′9″W), 2200 m, 1 ♂ 08.II.2014, P. Boyer *leg.* (MZUJ). **Itamonte** (Parque Nacional do Itatiaia, Pedra do Camelo) 1 ♀ 06.II.2014 (DNA voucher BLU 632), E. P. Barbosa *leg.* (ZUEC LEP 9961) (ZUEC); (Parque Nacional do Itatiaia, Estrada para Agulhas Negras), 2100 m, 1 ♀ 06.II.2014 (DNA voucher YPH 0442), E. P. Barbosa & A. Tacioli *leg.* (ZUEC-AVLF). **Camanducaia** (Monteverde), 1500–1800 m, 1 ♂ 03.XII.1988, O. & E.J. Mielke *leg.* (OM 19.467). *Espírito Santo*: **Dores do Rio Preto** (Parque Nacional do Caparaó, S region, 20°29′1″S 41°49′3″W), 1900 m, 1 ♂ 09.II.2014, P. Boyer *leg.* (MZUJ), (20°27′8″S 041°48′5″W), 2150 m, 6 ♂ and 1 ♀ 09.II.2014, P. Boyer *leg.* (MZUJ), (Pedra Menina, Serra do Caparaó via refúgio), 2100–2150 m, 14 ♂ and 1♀ 09.II.2014, T. Pyrcz *leg.* (prep. genit. 02/26.03.2014, 03/26.03.2014 J. Lorenc) (MZUJ), 2 ♂ 08.II.2014 (DNA vouchers YPH 0460, YPH 0461) 1 ♂ 08.II.2014, T. W. Pyrcz *leg.* (ZUEC LEP 9956, ZUEC LEP 9957, ZUEC LEP 9958) (ZUEC). *Rio de Janeiro*: 3 ♂ and 1 ♀ (syntypes) (ZMHB). **Itatiaia**, 1 ♂ I.1926, B. Pohl *leg.* (MZUSP), 1 ♂ X.1931, B. Pohl *leg.* (MZUSP), 1 ♂ 17.VII.1961, Mielke *leg.*, ex coll. D’Almeida (DZ 36.796), (Parque Nacional do Itatiaia) 1300–1700 m, 1 ♂ 21.I.1969, Mielke & Brown *leg.* (DZ 36.875), 1500 m, 1 ♂ 14.IV.1965, H. Ebert *leg.*, ex coll. H. Ebert (DZ 35.751 prep. genit. F. Dias 2016), 1700 m, 1 ♂ 08.VII.1956, H. Ebert *leg.*, ex coll. H. Ebert (DZ 36.856) (DZUP), 1 ♂ 17.VII.1961, Mielke *leg.* (OM 3.941), 2 ♂ 1800 m, 17.VII.1961, Mielke *leg.* (OM 3.940, OM 4.047) (OM), 2 ♂ 24.III.1967, H. Ebert *leg.*, ex coll. H. Ebert (DZ 27.362 prep. genit. F. Dias 2016, DZ 36.835), 1900 m, 2 ♂ 14.IV.1965, H. Ebert *leg.*, ex coll. H. Ebert (DZ 36.575, DZ 36.845), (S face), 2 ♂ 04.XI.1968, H. Ebert *leg.*, ex coll. H. Ebert (DZ 23.334 prep. genit. D. Dolibaina 2010) (DZ 23.334, DZ 36.785), 1 ♂ 08.XI.1968, H. Ebert *leg.*, ex coll. H. Ebert (DZ 36.665), 2000 m, 1 ♂ II.1960, H. Ebert *leg.*, ex coll. H. Ebert (prep. genit. F. Dias 2016) (DZ 36.765), 3 ♂ 13.IV.1965, H. Ebert *leg.*, ex coll. H. Ebert (DZ 36.420, DZ 36.876, DZ 36.885), 7 ♂ 24.III.1967, H. Ebert *leg.*, ex coll. H. Ebert (DZ 36.626, DZ 36.736, DZ 36.756 prep. genit. F. Dias 2016, DZ 36.795, DZ 36.865, DZ 36.985, DZ 37.026), (S face), 3 ♂ 16.I.1969, H. Ebert *leg.*, ex coll. H. Ebert (DZ 36.585, DZ 36.595, DZ 37.015), 1 ♀ 16.II.1979, O. & C. Mielke *leg.* (DZ 36.915) (DZUP), (Parque Nacional do Itatiaia, S22°21′8, W044°43′8), 2100 m, 1 ♂ 05.II.2014, P. Boyer *leg.* (MZUJ), (Parque Nacional do Itatiaia, 22°21′9″S 44°43′8″W), 2250 m, 11 ♂ and 1 ♀ 5.II.2014, P. Boyer *leg.* (MZUJ), 2300 m [♂ Fig 3a, ♀ Fig 3b], 5 ♂ 16.II.1979, O. & C. Mielke *leg.* (DZ 35.781, DZ 36.396, DZ 36.516, DZ 36.745, DZ 36.976), 2400 m, 8 ♂ and 1 ♀ 22.I.1969, O. Mielke & K. Brown Jr. *leg.* (DZ 36.525, DZ 36.565, DZ 36.725, DZ 36.775, DZ 36.895, DZ 36.945, DZ 36.975, DZ 37.005, DZ 37.025 prep. genit. F. Dias 2016), (Parque Nacional do Itatiaia, Brejo da Lapa, 22°21′22″S, 44°44′07″W), 2150 m, 2 ♂ 6–10.III.2011, O. Mielke & D. Dolibaina *leg.* (DZ 23.855 prep. genit. F. Dias 2016, DZ 36.675) (DZUP), (Parque Nacional do Itatiaia, km 6 da Estrada para Agulhas Negras), 2000 m, 1 ♂ 06.II.2014, P. Boyer *leg.* (MZUJ), (Parque Nacional do Itatiaia, km 13 da estrada para Agulhas Negras, 22°21′9″S 044°43′5″W), 2350 m, 3 ♂ 06.II.2014, P. Boyer *leg.* (MZUJ), (Parque Nacional do Itatiaia, estrada para Agulhas Negras), 2200–2250 m, 3 ♂ and 1 ♀ 05.II.2014, T. Pyrcz *leg.* (MZUJ), 13 ♂ and 6 ♀ 06.II.2014, T. Pyrcz *leg.* (prep. genit. 01/24.02.1015 J. Lorenc) (MZUJ), (Parque Nacional do Itatiaia, Morro do Couto) 1 ♀ 07.XII.2014 (DNA voucher BLU 709), A. H. B. Rosa *leg.* (ZUEC LEP 9960) (ZUEC). **Resende** 1 ♂ (prep. genit. – slide no. 1731 – Lee D. Miller) 21.I.1969, K. S. Brown *leg.* (ZUEC LEP 9959) (ZUEC). **Nova Friburgo** (Morro Mury), 1 ♂ 13.II.1957, Mielke *leg.* (OM 589), 1 ♂ 16.II.1969, Mielke *leg.* (OM 595) (OM). **Teresópolis**, 1500 m, 1 ♀ 16.II.1967, H. Ebert *leg.*, ex coll. H. Ebert (DZ 36.695 prep. genit. F. Dias 2016) (DZUP). *São Paulo*: **Campos do Jordão**, 2000 m, 1 ♂ 05–06.IV.1992, A. Moser *leg.* (CLAM).

### Redescription

*Male* (Fig 3a, 16d). *Head.* Antennae reaching 2/5 the length of costa, slender, dorsally dark brown, covered with rather sparse, minute, silver scales, ventrally orange, mostly naked, except for a few basal flagellomeres with sparse milky white scales, club mostly naked, made of 12 flagellomeres, slightly thicker than shaft, ventrally light orange; eyes blackish brown, densely hairy; labial palpi two times the length of head, covered mostly with blackish scales and hairy scales, considerably longer ventrally, except for a lateral row of sandy yellow scales; collar made of black elongated scales. Thorax: dorsally and ventrally black, sparsely scaly, tegulae covered with brown and golden scales; legs brown, tibiae and tarsi covered with greyish scales. *Wings.* FW (length: 25–28 mm) with a subacute apex and straight outer margins, very slightly produced below apex; fringes mostly brown except for some sandy yellow scales in the interspaces; FWD uniform blackish brown, lustrous and a shade lighter in the outer one-third; androconial patch not darker than the background, covering median one-fourth of wing surface, from discal cell distal edge to anal margin, entering discal cell. FWV grey brown and taupe brown, duller than on the dorsal surface, almost uniform, except for a few white scales in apical and subapical area, and occasionally for a faint, slightly lighter postdiscal line which separates a shade lighter distal from basal area; HW rounded with an undulating outer margin; fringes grey; HWD uniform blackish brown, lustrous, covered with long and rather dense hair in median half and along anal margin. HWV chocolate brown to blackish brown with a shade lighter postdiscal to submarginal band and a wavy blackish brown submarginal line; a mid-costal snow white streak of variable size, broken at Rs, extending to M_1_. *Abdomen.* dorsally covered with dark brown, ventrally and laterally with greyish brown scales. *Male genitalia* (Fig 7a, b). Tegumen triangular in lateral view, dorsum gently arched; uncus two-thirds the length of tegumen dorsum, slender, ventrally slightly constricted near base, almost straight, with an acute tip; subunci slender, about the same width throughout, with sharp tip, two-fifths the length of uncus, slightly uplifted; pedunculus blunt, prominent; vinculum wide, short; saccus deep, the length of tegumen dorsum, same width as vinculum in lateral view; valva slender, the length of tegumen + uncus, almost gradually narrowing from base to apex, with a number or rather small protrusions on dorsum terminating with a prominent apical teeth-like process slightly uplifted; aedeagus slender, the length of saccus + valva, gently arched, with a sharp tip, smooth, spoon-like at base; proximal opening one-third the length of aedeagus.

*Female* (Fig 3b). Marginally larger (FW length: 26–29 mm), and lighter on both the upper and underside, medium brown, with somewhat more noticeable HWV pattern, otherwise similar. *Female genitalia* (Fig 9g, h). Genitalia moderately flattened laterally in ventral view; papilla analis prominent, slightly irregular in lateral view, covered with rather dense setae, some of which, especially towards the apex considerably longer than the others; proximal unit, in lateral view, consisting of a well-sclerotized, narrow slat-like, smooth lamella postvaginalis, and two, strongly sclerotized, prominent lateral pocket-like folds with a heavily rippled surface, strongly compressed towards the entrance of ductus bursae; median unit with a wide slat-like, strongly sclerotized, lamella antegavinalis with smooth edges, touching but not merging ventro-laterally with lamella postvaginalis, enclosing from above the entrance to ductus bursae, with a deep and rather wide incision and a very shallow concavity; ductus bursae half the length of corpus bursae, tubular, strongly sclerotized, wide and approximately the same width throughout, entrance of bursa with a slightly sclerotized bulbous structure in ventral position; seminal duct originating at the entrance of bursa; bursa copulatrix rounded, with two wide signa extending over two-thirds of its length.

### Comments

This species was described based on three males and one female (Thieme 1905) overlooked the female specimen, recognizing errouneously all syntypes as male specimens, from the Brazilian state of Rio de Janeiro. There are, however, two high mountainous areas in that state, the Serra dos Órgãos, immediately north of the city of Rio de Janeiro, and the Serra da Mantiqueira shared between the states of Rio de Janeiro, São Paulo and Minas Gerais (Fig 25). All examined specimens are from the latter range. This species occurs at higher elevations than most sympatric congeners and is found generally above 2000 m, most frequently at 2200–2400 m at the upper limit of cloud forests, where it is the dominant representative of *Praepedaliodes*.

**Fig 26:**
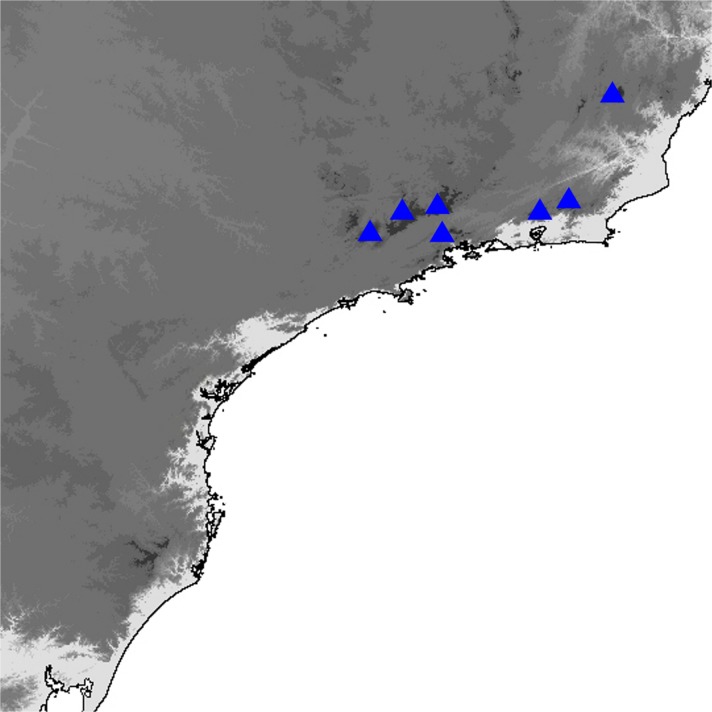
*Praepedaliodes pawlaki* n. sp. distribution map.

***Praepedaliodes pawlaki*** Pyrcz & Boyer n. sp.

(Figs 3c, d, 7c, d, 9c, d and 26)

Type locality: Brejo da Lapa, Parque Nacional do Itatiaia, Itatiaia, Rio de Janeiro, Brazil

### Type material

Holotype ♂ with the following labels: /Brasil, Rio de Janeiro, Itatiaia, P.N. Itatiaia 2150 m, 22°21′22″S/44°44′03″W, Brejo da Lapa, 06-10.iii.2011, O.-C.Mielke & Dolibaina *leg.*/ DZ 23.715/ HOLOTYPUS/ HOLOTYPE *Praepedaliodes pawlaki* Pyrcz & Boyer, det 2016/ (prep. genit. F. Dias 2016). Deposited in the Coleção Entomológica Padre Jesus Santiago Moure, Departamento de Zoologia, Universidade Federal do Paraná, Curitiba, Paraná, Brazil (DZUP).

Allotype ♀ with the following labels: /P.N. Itatiaia Resende [Itatiaia], R[io de]J[aneiro], 12.I.1973, 1400 m, Mielke/ DZ35.771/ ALLOTYPUS/ ALLOTYPE *Praepedaliodes pawlaki* Pyrcz & Boyer, det 2016/. Deposited in the Coleção Entomológica Padre Jesus Santiago Moure, Departamento de Zoologia, Universidade Federal do Paraná, Curitiba, Paraná, Brazil (DZUP).

*Paratypes*. (40 ♂ and 18 ♀): BRAZIL: *Minas Gerais*: **Alto Caparaó** (Parque Nacional do Caparaó), 1500 m, 1 ♂ 06-08.II.1987, Mielke & Casagrande *leg.* (OM 13.750), 2000–2200 m, 2 ♂ and 1 ♀, same data as above (OM 13.744, OM 13.745, OM 13.748) (OM), 1 ♂ XII.2012 (DNA voucher BLU 372 prep. genit. E. P. Barbosa 2012), A. V. L. Freitas, L. A. Kaminski & C. A. Iserhard *leg.* (ZUEC LEP 9966) (ZUEC). **Delfim Moreira** (15 Km SE), 1500–1700 m, 1 ♂ and 2 ♀ 16-17.I.2004, Mielke & Casagrande *leg.* (DZ 23.765 prep. genit. F. Dias 2012, DZ 23.635, DZ 34.885), 1 ♂ 22-23.I.2004, Mielke & Casagrande *leg.* (DZ 36.825) (DZUP). *Rio de Janeiro*: **Itatiaia** (Parque Nacional do Itatiaia, South face), 1800 m, 1 ♀ 24.III.1967, H. Ebert *leg.*, ex coll. H. Ebert (DZ 36.965), 1900 m, 1 ♀ 14.IV.1965, H. Ebert *leg.*, ex coll. H. Ebert (DZ 36.635), (Serra do Itatiaia, S face), 2200 m, 1 ♀ 29.VII.1969, H. Ebert *leg.*, ex coll. H. Ebert (DZ 36.505), 1400 m, 1 ♀ 12.I.1973, Mielke *leg.* (DZ 35.771), 1600 m, 1 ♂ 12.I.1973, Mielke *leg.* (DZ 36.515, DZ 36.755), (DZUP), 2 ♂ 03.II.1968, K. S. Brown Jr. *leg.* (ZUEC LEP 9967, ZUEC LEP 9968) (ZUEC). **Nova Friburgo** (Pico Caledônia), 2219 m, 1 ♀ 24.I.1996, O. & C. Mielke *leg.* (OM 41.999) (OM). **Petrópolis**, 1200 m, 1 ♀ 30.VII.1968, H. Ebert *leg.*, ex coll. H. Ebert (DZ 36.410) (DZUP). *São Paulo*: **Campos do Jordão**, 1600–1700 m, 1 ♂ 22-25.I.1992, Mielke & Casagrande *leg.* (OM 28.503), 1700 m, 1 ♂ and 4 ♀ I.1966, H. Ebert *leg.*, ex coll. H. Ebert (DZ 37.445 prep. genit. F. Dias 2013, DZ 36.645, DZ 36.705, DZ 36.925, DZ 37.035 prep. genit. F. Dias 2013), 1800–2000 m, 1 ♀ 08-12.II.1982, Mielke & Casagrande *leg.* (DZ 36.935) (DZUP), (22°46′1″S 45°36′8″W), 1500 m, 3 ♂ and 1 ♀ 03-04.II.2014, P. Boyer *leg.* (MZUJ), 4 ♂, same data as above, P. Boyer *leg.* (MZUJ), (Trilha do Zigue Zague), 1450–1500 m, 1 ♂ 13.II.2013, T. Pyrcz *leg.* (MZUJ), (Alto do Capivari), 1790 m, 1 ♂ 17-20.I.2014 (DNA voucher YPH 0397), B-879/CJACP, A. V. L. Freitas *leg.* (ZUEC-AVLF). **Pindamonhangaba** (Pico do Itapeva), 1850–1900 m, 10 ♂ and 1 ♀ 11.IV.2012, T. Pyrcz *leg.* (prep. genit. 03/20.05.2012, 04/20.05.2012, 08/20.05.2012, 02/29.03.2013, 01/13.06.2013, 02/13.03.2015 J. Lorenc) (MZUJ) [♂ Fig 3c, ♀ Fig 3d], 1 ♂ 13.IV.2012, T. Pyrcz *leg.* (MZUJ), 1 ♂ 27.IV.2012, T. Pyrcz *leg.* (prep. genit. 03/29.03.2013 J. Lorenc) (MZUJ), (Pico do Itapeva, forest trail), 1850–1900 m, 2 ♂ 03.II.2014, T. Pyrcz *leg.* (prep. genit. 01/26.03.2015 J. Lorenc) (MZUJ), 2 ♂ 04.II.2014, T. Pyrcz *leg.* (MZUJ), 1 ♂ 03.II.2014 (DNA voucher BLU 624), A. V. L. Freitas *leg.* (ZUEC LEP 9964), 1 ♀ 01.II.2014 (DNA voucher BLU 622), A. V. L. Freitas leg (ZUEC LEP 9965) (ZUEC); 1 ♀ 03.II.2014 (DNA voucher YPH 0444), A. V. L. Freitas *leg.* (ZUEC-AVLF). **Piquete** (Barreira do Piquete), 1400–1600 m, 1 ♂ 15.II.1984, Mielke & Casagrande *leg.* (DZ 36.815) (DZUP). **São José do Barreiro** (Parque Nacional da Serra da Bocaina, Antena), 1750–1800 m, 1 ♂ 13.II.2014, T. Pyrcz *leg.* (prep. genit. 308/28.12.2015, J. Lorenc) (MZUJ), (DNA voucher YPH 0449).

### Diagnosis

All brown upperside as in other sympatric congeners; differs from *P. francinii* n. sp. and *P. landryi* n. sp. in wider wings, the slightly concave FW outer margin and a blunt apex; most notably differs from other congeners in the HWV pattern, particularly in the lack of any silver scaling or submarginal dots, and the shape of the postdiscal line which is well incurved basally in space CuA_1_-CuA_2_, and from *P. exul* by lacking a HWV white costal patch.

### Description

*Male* (Fig 3c). Head: Antennae reaching 2/5 the length of costa, slender, dark brown, naked, orange ventrally on the club, with 12 flagellomeres, only slightly thicker than shaft; eyes chocolate brown, densely setose; labial palpi two times the length of head, covered with blackish and brown hairy scales and laterally with some milky white scales; collar with taupe brown elongated scales. *Thorax.* Dorsally and ventrally black, sparsely scaly, tegulae covered with taupe brown scales; legs black, tibiae and tarsi covered with brown scales. *Wings.* FW (length: 24–26 mm) with a blunt apex, a gently concave outer margin slightly truncate below apex, fringes all brown except for a few whitish scales on tornus; FWD uniform seal brown, lustrous; androconial patch a shade darker, very large, covering median half of wing surface, from subapical area to anal margin, widely entering discal cell. FWV seal brown, lighter and duller than on the dorsal surface; a shade darker median patch, smaller than the area covered by the androconial patch on the dorsum; marginal area a shade darker, chocolate brown; HW rounded with an undulating outer margin; fringes brown; HWD uniform seal brown, lustrous, slightly hairy in basal, postbasal area and along anal margin. HWV seal brown, dull, with a light chocolate brown overcast from base to postdiscal line and along outer margin, the remaining area, from postdiscal to submarginal line a shade lighter; postdiscal line with a noticeable protrusion in CuA_1_-CuA_2_, directed basally; in some specimens a short and faint, whitish mid-costal streak, in some individuals faint, submarginal dots not apparent; in some individuals the postdiscal - submarginal area a shade lighter; marginal area marked with crimson red. *Abdomen*. Dorsally covered with black, ventrally and laterally with grey scales. *Genitalia* (Fig 7c, d). tegumen subtriangular in lateral view, elongated vertically which is noticeable in its length between subunci and pedunculus base, as long as the gently arched dorsum; uncus two-fifths the length of tegumen dorsum, slender, ventrally slightly constricted near base, almost straight, with an acute tip; subunci slender, about the same width throughout, with a sharp tip, two-thirds the length of uncus, almost straight; pedunculus blunt, small; vinculum wide, short; saccus deep, the length of tegumen dorsum, same width as vinculum in lateral view; valva slender, marginally shorter than tegumen + uncus, noticeably wider in basal half, with three or teeth-like processes on dorsum, gradually larger from basal to distal one, one or two in basal one third, marking the end of the wider section, one, more prominent in the middle, and one subapical, noticeably longer than the remaining ones, uplifted, tip blunt; aedeagus slender, the length of saccus + valva, nearly straight, with a sharp tip, smooth, spoon-like at base; proximal opening two-fifths the length of aedeagus.

*Female* (Fig 3d). Similar in size (FW length: 25–26 mm), wing colours are lighter brown, medium or chestnut, particularly on the HWV, which makes out better the pattern of lines and bands, and also the white costal spot is more noticeable, the reddish HWV pattern is, however, not apparent. *Genitalia* (Fig 9c, d). Flattened laterally in ventral view; papilla analis prominent, gently rounded in lateral view, covered with sparse, and rather delicate setae of similar length throughout; proximal unit, in lateral view, consisting of a moderately sclerotized slat-like, narrow lamella postvaginalis extending into two, moderately sclerotized, prominent lateral pocket-like folds with a moderately rippled surface, strongly compressed towards the entrance of ductus bursae; median unit with a slat-like, rather wide and strongly sclerotized, lamella antevaginalis with smooth edges, touching but not merging ventro-laterally with lamella postvaginalis, enclosing from above the entrance to ductus bursae, with an incision and a barely noticeable shallow concavity; ductus bursae two-thirds the length of corpus bursae, tubular, strongly sclerotized, compressed in the middle, entrance of bursa with a stout, strongly sclerotized bulb in dorsal position; ductus seminalis originating at the entrance of bursa; bursa copulatrix round, with two wide signa extending over two-thirds of its length.

### Etymology

Dedicated to a Polish medical doctor and amateur lepidopterist, Leszek Pawlak, the first author’s companion on several entomological expeditions.

### Comments

**Fig 27:**
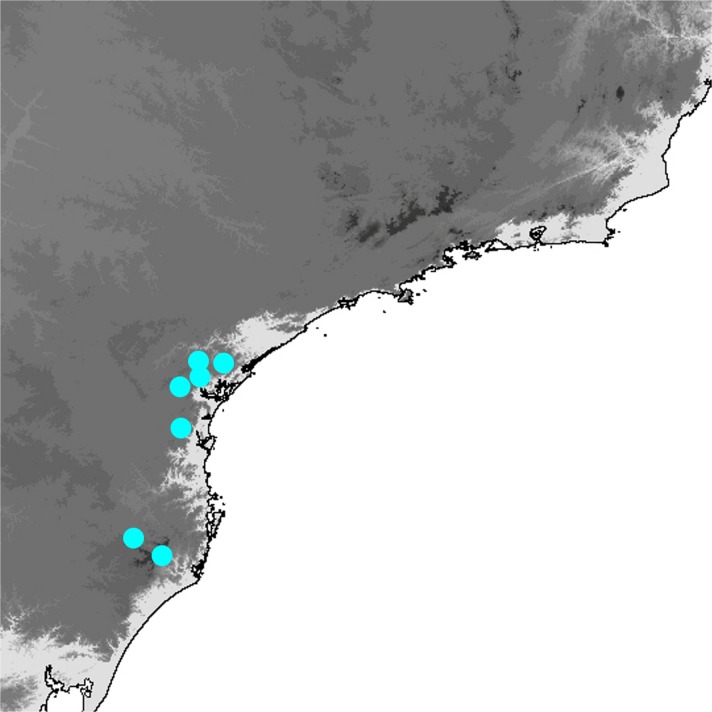
*Praepedaliodes zaccae* n. sp. distribution map.

*Praepedaliodes pawlaki* n. sp. is found in the Serra da Mantiqueira, the Serra do Caparaó and the northern portion of Serra do Mar (Bocaina region) (Fig 26), where it is parapatric below its relative *P. exul* (Fig 25) Southwards in the Serra do Mar it is replaced allopatrically by another ally, *P. zaccae* Dolibaina, Dias & Pyrcz n. sp. (Fig 27). Genetic evidence clearly shows that *P. pawlaki* n. sp., *P. exul, P. sequeirae* n. sp. and *P. zaccae* n. sp. are closely related species, but there is enough morphological evidence sustaining their separate status, and when it comes the former three, they are ecologically separated in altitude. *Praepedaliodes pawlaki* n. sp. occurs at 1500–1600 m generally. *Praepedaliodes exul* occurs at higher elevations, and *P. sequeirae* n. sp. even higher, above 2300 m. *Praepedaliodes pawlaki* n. sp. is rather uncommon and restricted to well-preserved patches of forest where it flies in small clearings with abundant species of *Chusquea*. On the Pico do Itapeva, in the Serra da Mantiqueira, it is syntopic with *P. francinii* n. sp. and *P. landryi* n. sp. It never comes to the ground level, and can be observed frequently when feeding on the nectar of different species of trees in the subcanopy.

***Praepedaliodes zaccae*** Dolibaina, Dias & Pyrcz n. sp.

(Figs 3e, f, 7e, f, 9a, b and 27)

Type locality: Morro do Anhangava, Quatro Barras, Paraná, Brazil

### Type material

Holotype ♂ with the following labels: /Brasil, Paraná, Quatro Barras Morro do Anhangava – 1470 m, 15.IV.2010, D. R. Dolibaina *leg.* /DZ 16.746 /HOLOTYPUS /HOLOTYPE *Praepedaliodes zaccae* Dolibaina, Dias, Pyrcz, det. 2016 /Gen. Prep. Dolibaina 2010/ (prep. genit. D. Dolibaina 2010). Deposited in the Coleção Entomológica Padre Jesus Santiago Moure, Departamento de Zoologia, Universidade Federal do Paraná, Curitiba, Paraná, Brazil (DZUP).

Allotype ♀ with the following labels: /Brasil, Paraná, Quatro Barras Morro do Anhangava – 1470 m, 15.IV.2010, D. R. Dolibaina *leg.* /DZ 23.414 /ALLOTYPUS /ALLOTYPE *Praepedaliodes zaccae* Dolibaina, Dias, & Pyrcz, det. 2016/. Deposited in the Coleção Entomológica Padre Jesus Santiago Moure, Departamento de Zoologia, Universidade Federal do Paraná, Curitiba, Paraná, Brazil (DZUP).

*Paratypes*. (27 ♂ and 10 ♀): BRAZIL: *Paraná*: 1 ♂ **Campina Grande do Sul** (Pico Paraná), 1300–1500 m, 1 ♂ 27.II.1994, O. & C. Mielke *leg.* (OM 38.065) (OM). **Guaratuba** (Pontal do Itararé), 1350 m, 1 ♀ 31.I.2004, Mielke *leg.* (OM 64.388) (OM), 1400 m, 1 ♀ 14.I.2005, Mielke *leg.* (DZ 23.464) (DZUP). **Morretes** (Alto da Serra), 800 m, 06.II.1966, Mielke *leg.* (OM 8.808) (OM), 1 ♂ 16.II.1975, Mielke (DZ 16.767) (DZUP), (Serra Graciosa, Rio Taquari), 800–850 m, 29.IV.2012, T. Pyrcz *leg.* (prep. genit. 01/13.11.2012 J. Lorenc.). **Quatro Barras** (Morro do Anhangava), 1 ♀ 25.II.2009, Dolibaina *leg.* (DD 291) (DD), 1325 m, 2 ♂ 20.I.2010, 08.V.2009, Dolibaina *leg.* (DD 289, DD 287) (DD), 1370 m, 1 ♂ 11.III.2010, Dolibaina *leg.* (DD 290) (DD), 1470 m, 5 ♂ and 2 ♀ 15.IV.2010, Dolibaina *leg.* (DZ 19.819 prep. genit. D. Dolibaina 2010, DZ 16.746 prep. genit. D. Dolibaina 2010, DZ 20.261 prep. genit. D. Dolibaina 2010, DZ 19.330 prep. genit. D. Dolibaina 2010, DZ 23.414) (DZUP), (DD 298, DD 299) (DD), 2 ♂ 7.II.2011, Dolibaina *leg.* (DD 295, DD 296) (DD), 1 ♂ 7.IV.2011, Dolibaina *leg.* (DD 292) (DD), 900-1600 m, 1 ♀ 13.II.2014 (DD 276) (DD), (Morro do Anhangava, Campos de Altitude), 1425 m, 4 ♂ 07.V.2009, 04.XI.2009, 25.III.2009, Carneiro *leg.* (DD 293 prep. genit. Dolibaina 2010, DD 294, DD 280, DD 288) (DD). **Tijucas do Sul** (Morro do Araçatuba), 1670 m, 2 ♀ 14.II.2014, Carneiro, Dias & Dolibaina *leg.* (DZ 35.741, DZ 35.761) (DZUP). *Santa Catarina*: **Urubici** (Serra do Corvo Branco), 1200–1300 m, 1 ♂ 11.II.2015, T. Pyrcz *leg.* (prep. genit. 03/08.04.2015 J. Lorenc) (MZUJ), 1 ♂ 11.II.2015 (DNA voucher BLU 718), T. W. Pyrcz *leg.* (ZUEC LEP 9963) (ZUEC). **Urupema** (Morro das Antenas, 27°55′58″S 49°51′33″W), 1550–1600 m, 5 ♂ and 2 ♀ 06.II.2015, P. Boyer *leg.* (MZUJ), 1500–1700 m, 5 ♂ and 3 ♀ 06.II.2015, T. Pyrcz *leg.* (prep. genit. 04/08.04.2015 J. Lorenc, prep. genit. 04/10.06.2015 J. Lorenc, prep. genit. 302/16.12.2015 J. Lorenc) (MZUJ), 1300 m, 1 ♂ 08.II.2015 (DNA voucher BLU 715), T. W. Pyrcz *leg.* (ZUEC LEP 9962) (ZUEC).

### Diagnosis

All brown upperside as in other sympatric congeners; differs from *P. pawlaki* n. sp.*, P. landryi* n. sp. and *P. francinii* n. sp. in wider, rounded hindwings; little patterned underside most similar to *P. pawlaki* n. sp. but without any trace of reddish margins, and to *P. exul* but with lighter HWV ground colour and without the characteristic HWV white costal patch; considerably smaller than *P. duartei* n. sp.

### Description

*Male* (Fig 3e). *Head*. Antennae reaching 2/5 the length of costa, slender, dark brown, ventrally orange, naked, club with 12 flagellomeres, only slightly thicker than shaft, ventrally orange; eyes chocolate brown, densely setose; labial palpi two times the length of head, covered with blackish brown hairy scales, with a lateral row of sandy yellow scales; collar with brown elongated scales. *Thorax.* Dorsally and ventrally black, covered with rather dense brown and golden scales, tegulae covered with brown scales with a dark blue sheen; legs brown, tibiae and tarsi covered with brown and grey yellow scales. *Wings.* FW (length: 24–27 mm) with a blunt apex and straight outer margins, marginally truncate below apex; fringes very short, intermittently brown and sandy yellow; FWD uniform chocolate brown, lustrous; androconial patch a shade darker, very large, covering median half of wing surface, from discal cell distal edge to anal margin, including distal half of discal cell. FWV chocolate brown, a shade lighter than on the dorsal surface, lustrous; without any pattern except for a faint, sinuate, submarginal darker brown line. HW rounded with an undulated outer margin; fringes very short, brown; HWD uniform chocolate brown, lustrous, sparsely hairy along anal margin. HWV chocolate brown with a darker basal, postbasal and marginal areas, a faint, slightly lighter wide median band with an irregular, outer edge, and a noticeable basal notch on vein CuA_2_. *Abdomen.* Black, dorsally covered with dark brown, ventrally and laterally with grey brown scales. *Genitalia* (Fig 7e, f). Tegumen subtriangular in lateral view, elongated vertically which is noticeable in its length between subunci and pedunculus base, as long as the gently arched dorsum; uncus two-fifths the length of tegumen dorsum, slender, ventrally slightly constricted near base, almost straight, with an acute tip; subunci slender, about the same width throughout, with a sharp tip, two-thirds the length of uncus, almost straight; pedunculus sharp, small; vinculum wide, short; saccus deep, the length of tegumen dorsum, slightly wider than vinculum in lateral view; valva slender, as long as tegumen + uncus, wider in basal half, then gradually narrowing, dorsal surface irregular with several small protrusions, terminating with a long apical, sharp, uplifted process; aedeagus slender, marginally longer than saccus + valva, nearly straight, with a sharp tip and a noticeable crest, otherwise smooth, spoon-like at base; proximal opening two-fifths the length of aedeagus.

*Female* (Fig 3f). Slightly larger (FW length: 27–28 mm), with a much more scalloped HW outer margin. FWD and HWD colour pattern lighter, sepia brown. FWV and HWV also lighter, pale sepia brown with a much better marked pattern; FWV with an irregular postdiscal line extending from costa to vein CuA_2_, basally dusted with chocolate brown; a series of minute subapical, yellow dots; and a submarginal irregular line defining a darker brown marginal area; some whitish scales in the apical area. HWV with a similar pattern of bands as in the male, but more contrasting, because of the chocolate edging of the postdiscal and submarginal band; a row of seven minute, yellow submarginal dots. *Genitalia* (Fig 9a, b). Moderately flattened laterally in ventral view; papilla analis prominent, gently rounded in lateral view, covered mostly with short and delicate setae, except for some considerably longer ones near apex; proximal unit, in lateral view, consisting of a sclerotized narrow, smooth lamella postvaginalis extending into two, strongly sclerotized, prominent lateral pocket-like folds with a heavily rippled surface, compressed towards the entrance of ductus bursae; median unit with a slat-like, wide and strongly sclerotized lamella antevaginalis with smooth edges, touching but not merging ventro-laterally with lamella postvaginalis, enclosing from above the entrance to ductus bursae, with a wide incision; ductus bursae half the length of corpus bursae, tubular, strongly sclerotized, extremely wide at base, twice as the width in the middle, entrance of bursa with a moderately sclerotized irregular bulbous structure in ventral position; ductus seminalis originating at the entrance of bursa; bursa copulatrix oval, with two wide signa extending over three-fifths of its length.

### Etymology

This species is dedicated to our friend Dr. Thamara Zacca, a Brazilian entomologist, author of several papers dedicated to Satyrinae butterflies.

### Comments

This species is the only *Praepedaliodes* found exclusively in the south of the Atlantic Forest, most specifically in the Serra do Mar (Serra da Graciosa, Morro do Anhangava) and the Serra Geral from Paraná and Santa Catarina (Fig 27). It is most closely related to *P. exul* + *P. pawlaki* n. sp., as indicated by molecular evidence, and their affinities can be seen in male genitalia and HWV colour patterns. Their separate specific status is sustained by consistent external morphological differences. In the Serra do Mar in Paraná, the species occurs from 800 to 1700 m, where males are frequently seen on the top of the Morro do Anhangava (~1400 m) and Morro do Araçatuba (~1700 m), where their fast and irregular flight made their collection difficult. In Urupema, further south, the species flies at altitudes around 1500–1700 m where it is not rare. The species has not been found east of Urupema in the Serra do Corvo Branco range at 1200–1300 m where *P. francinii* n. sp. was common and *P. landryi* n. sp. was also recorded.

**Fig 28:**
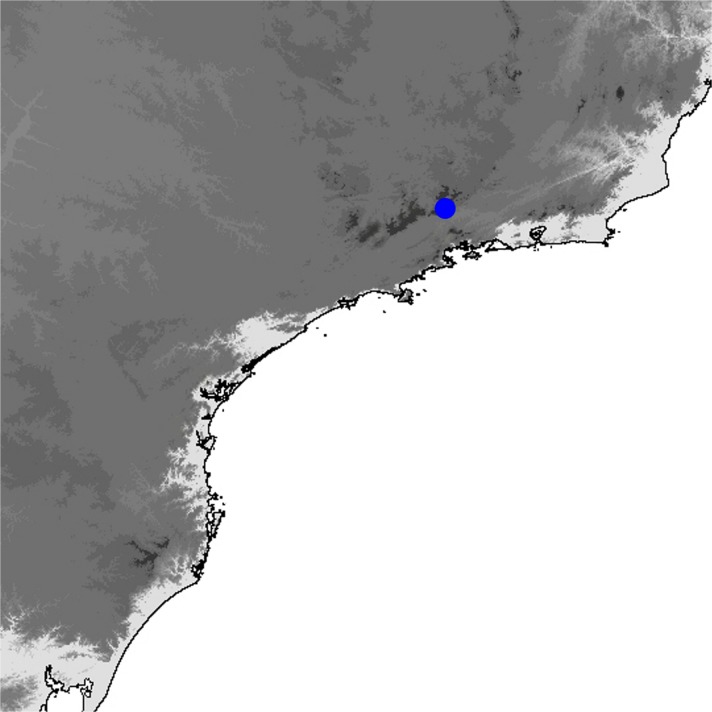
*Praepedaliodes sequeirae* n. sp. distribution map.

***Praepedaliodes sequeirae*** Pyrcz, Dias & Dolibaina **n. sp.**

(Figs 2e, f, 5e, f, 9e, f and 28)

*Faunula monticola* Zikan & Zikan, 1968: 49; nomen nudum

Type locality: Morro do Couto, Parque Nacional do Itatiaia, Itatiaia, Rio de Janeiro, Brazil

### Material examined

Holotype ♂ with the following labels: /Parte Alta, Morro do Couto, Parque Nacional do Itatiaia, Itatiaia, Rio de Janeiro, Brazil, 07.XII.2014, Rosa A. H. B., BLU 707 / ZUEC LEP 9974 /. Deposited in the Museu de Zoologia Adão José Cardoso, Universidade Estadual de Campinas, Campinas, São Paulo, Brazil (ZUEC).

*Paratypes* (28 ♂ and 5 ♀): BRAZIL: *Minas Gerais*: **Itamonte** (Parque Nacional do Itatiaia), 2300 m, 2 ♂ 05.II.2014 (DNA vouchers BLU 630, BLU 633), E. P. Barbosa *leg.* (ZUEC LEP 9969, ZUEC LEP 9970), 2 ♂ 1 ♀ 06.II.2014 (DNA vouchers BLU 634, BLU 635, BLU 631), E. P. Barbosa *leg.* (ZUEC LEP 9971, ZUEC LEP 9972, ZUEC LEP 9973) (ZUEC), 1 ♂ 05.II.2014 (DNA voucher YPH 0441), E. P. Barbosa & A. Tacioli *leg.* (ZUEC-AVLF). *Rio de Janeiro*: **Itatiaia**, 2200 m, 1 ♂ II.1960, Barth *leg.* (DZ 23.484), (Parque Nacional do Itatiaia) 2 ♂ 21.I.1969, K. S. Brown Jr. *leg.* (ZUEC LEP 9976, ZUEC LEP 9977), 1 ♂ 8.I.1971, Mielke *leg.* (DZ 23.474), 2300 m, 1 ♂ and 1 ♀ 16.II.1979, O. & C. Mielke *leg.* (DZ 19.773 prep. genit. D. Dolibaina 2010, DZ 19.456), (Parque Nacional do Itatiaia, km 13 da estrada para Agulhas Negras, 22°22′5″S 44°42′1″W), 2350 m, 7 ♂ and 1 ♀ 6.II.2014, P. Boyer *leg.* (MZUJ), same data as above 10 ♂ and 2 ♀ T. Pyrcz *leg.* (prep. genit. 01/24.04.2014, 03/13.03.2015, J. Lorenc) (MZUJ) [♂ Fig 23, ♀ Fig 2f], (Parque Nacional do Itatiaia, Morro do Couto), 1♂ 07.XII.2014 (DNA voucher BLU707, BLU708), A. H. B. Rosa *leg.* (ZUEC LEP 9975), (ZUEC).

### Diagnosis

Differs from other sympatric congeners in the all blackish-brown upperside which is darker than in other species, and most notably in the narrower wings, and truncate FW apex, as well as in the lack of any HWV pale pattern, except for a faint costal streak in some individuals.

### Description

*Male* (Fig 2e). *Head*. Antennae reaching 2/5 the length of costa, slender, composed of 39 flagellomeres, orange, covered with dense black scales, particularly in basal half and on dorsal side, and sparse sandy yellow scales, club, with 12 flagellomeres, only slightly thicker than shaft; eyes lustrous, chocolate brown, densely setose; labial palpi two times the length of head, covered with blackish and chestnut hairy scales and laterally with some sparse golden scales; collar with taupe brown and sandy yellow elongated scales. *Thorax.* Dorsally and ventrally black, covered with rather dense and long brown and golden brown scales, tegulae covered with taupe brown scales; legs black, femora, tibiae and tarsi covered with dense brown scales, with sparse sandy yellow scales and numerous black spines. *Wings.* FW (length: 24–26 mm) elongated, with an acute apex, outer margin truncate below apex and a gently concave, fringes all grey brown except some golden scales at vein ends; FWD uniform blackish brown, lustrous; androconial patch a shade darker, limited to median part of the wing, along distal edge of discal cell, marginally penetrating into it, compact. FWV dark brown, a shade lighter than on the FW, almost uniform except for the blackish costa and some sparse white scales along outer margin. HW oval with a slightly undulating outer margin; fringes grey brown; HWD uniform blackish brown, lustrous, densely scaly in basal, postbasal and median areas. HWV uniform dark brown, with lilac and grey scales concentrated along outer margin along veins, in some individuals a faint, dirty white mid-costal streak. *Abdomen.* Black, dorsally covered with brown scales and dense hair scales, especially in basal part, ventrally and laterally covered with grey brown scales. *Genitalia* (Fig 5e, f). Tegumen subtriangular in lateral view, elongated vertically which is noticeable in its length between subunci and pedunculus base, as long as the gently arched dorsum; uncus two-fifths the length of tegumen dorsum, slender, with a small ventral constriction near base, almost straight, with an subacute tip; subunci stout, gradually narrowing towards a subacute apex, half the length of uncus, almost straight; pedunculus sharp, small; vinculum wide, short; saccus deep, marginally shorter than tegumen dorsum, same width as vinculum in lateral view; valva stout, slightly shorter than tegumen + uncus, noticeably wider in basal half, dorsally covered with minute spines in apical two-thirds, with one massive dorsal process marking the end of the wider section and one short, teeth-like subapical process, tip blunt; aedeagus slender, slightly longer than saccus + valva, nearly straight, with a sharp tip and a spiny crest, otherwise smooth, spoon-like at base; proximal opening two-fifths the length of aedeagus.

*Female* (Fig 2f). Sexual dimorphism slight, size nearly similar (FW length: 25–26 mm), and expressed in the lighter auburn brown of the FWD and HWD of the female, and russet brown FWV and HWV, with a light overcast of golden yellow scales, on the FW subapical area and on most of the median area of the HW, underlying a lighter median band. *Genitalia* (Fig 9e, f). Flattened laterally in ventral view; papilla analis prominent, gently rounded in lateral view, covered with short but rather dense, delicate setae, somewhat longer at apex; proximal unit, in lateral view, consisting of a well-sclerotized slat-like, smooth lamella postvaginalis extending into two, moderately sclerotized, prominent lateral pocket-like folds with a strongly rippled surface, compressed towards the entrance of ductus bursae; median unit with a slat-like, strongly sclerotized and wide lamella antevaginalis with smooth edges, narrowly merging ventro-laterally with lamella postvaginalis, enclosing from above the entrance to ductus bursae, with a shallow but well-marked concavity; ductus bursae half the length of corpus bursae, tubular, strongly sclerotized, moderately wide and slightly compressed in the middle, entrance of bursa with a moderately sclerotized bulbous structure in ventral and a fold in dorsal position; ductus seminalis originating at the entrance of bursa; bursa copulatrix oval, with two wide signa extending over half of its length.

### Etymology

This species is dedicated to the late Dr. Vanessa Sequeira, a Portuguese-German-Brazilian biologist, ecologist and environmental activist.

### Comments

This is perhaps the most intriguing species of *Praepedaliodes*, the only narrow endemic species, which is found only in the Itatiaia massif (Fig 28), and it is also the species that occurs at the highest elevations within the genus. Its habitat is the forest – alpine grassland transitional zone covered with dense, dwarf species of *Chusquea*. *Praepedaliodes sequeirae* n. sp. is morphologically quite exceptional. Its wings are elongated and the pattern is simplified, and the overall appearance is strongly reminiscent of some Andean paramo dwelling Pronophilina suggesting that adaptations to high elevation open habitat may lead to parallel evolution. *Praepedaliodes sequeirae* n. sp. externally resembles the Andean páramo and puna genera *Altopedaliodes* Forster, 1964 or *Punapedaliodes* Forster, 1964. Morphologically *P. sequeirae* n. sp. is so different from all other *Praepedaliodes* that Zikán & Zikán (1968) mentioned this taxon as an undescribed species of the genus *Faunula* C. Felder & R. Felder, 1867 *.* Its male genitalia is unlike any congener, with a short valva culminating dorsally with a massive process. Its relationships with *P. exul* + *P. pawlaki* n. sp. are revealed by the slender uncus and subunci and supported by molecular data.

### Genetic divergence and phylogenetic inference

The Bayesian analysis of molecular data showed that the genus *Praepedaliodes* is a strongly supported monophyletic clade. The internal organization showed that the genus can be roughly separated into five groups: (1) *P. phanias* + *P. duartei* n. sp.; (2) *P. francinii* n. sp.; (3) *P. landryi* n. sp.; (4) *P. amussis* + *P granulata*; and (5) *P. sequeirae* n. sp. + (*P. exul* + *P. pawlaki* n. sp. + *P. zaccae* n. sp.) (Fig 17). Except by the last three, all species of *Praepedaliodes* are well supported by molecular data. However, DNA barcodes not always allow to discriminate between closely related but ecologically and morphologically different species (Burns *et al* 2007). Intraspecific barcode distances ranged from 0 to 2.6%, and interspecific distances ranged from 0 to 10.5%, in some cases overlapping with the former. The genetic distances among the analysed species of *Praepedaliodes* are presented (Fig 18 and Table 2).

**Table 2:** Genetic distances among all *Praepedaliodes* species (based on barcode sequences).

Species	1	2	3	4	5	6	7	8	9
1. *P. phanias*									
2. *P. duartei*	0.065								
3. *P. francinii*	0.082	0.072							
4. *P. landryi*	0.090	0.070	0.046						
5. *P. granulata*	0.083	0.098	0.070	0.049					
6. *P. amussis*	0.098	0.099	0.077	0.063	0.081				
7. *P. sequeirae*	0.075	0.091	0.058	0.062	0.073	0.078			
8. *P. exul*	0.079	0.083	0.058	0.058	0.081	0.074	0.007		
9. *P. pawlaki*	0.075	0.087	0.056	0.059	0.073	0.077	0.008	0.013	
10. *P. zaccae*	0.080	0.086	0.062	0.057	0.078	0.073	0.004	0.004	0.011

## Discussion

### Diversity and distribution patterns

The genus *Praepedaliodes* proves far more diverse than hitherto recognized. Its species richness prior to this study was only four, whereas it has now more than doubled to ten. Since most high elevation habitats in the region were thoroughly sampled and numerous collections examined, this figure most probably represents the total number of species of *Praepedaliodes*, unless some of the allopatric populations of *P. phanias* are attributed a separate specific status. This is not the only example of an Atlantic Forest genus of Satyrinae whose species richness has increased considerably due to recent studies. Another example is the genus *Moneuptychia* Forster, 1964 belonging to the subtribe Euptychiina, a predominantly premontane and montane genus whose species also increased from two to eight in the last decade (Freitas 2007, Freitas *et al* 2010, 2015), with at least five more undescribed species identified (Freitas *et al* in prep.).

The generic range of *Praepedaliodes* extends along most of the Atlantic Forest, however most species are restricted to coastal ranges, and the generic distribution overlaps entirely with the area of distribution of the bamboo species of the genus *Chusquea*, the larval host plants (Fisher *et al* 2014). Only one species, *P. phanias*, is widespread throughout this vast region and is found in its western part, as far as the province of Misiones in Argentina and eastern Paraguay. Other representatives of *Praepedaliodes* are geographically more restricted, even if only one species, *P. sequeirae* n. sp., is a single mountain range endemic. The Serra da Mantiqueira appears to be the diversity hot-spot of the genus with as many as seven species occurring in this range (*P. phanias, P. amussis, P. exul, P. francinii* n. sp.*, P. pawlaki* n. sp.*, P. landryi* n. sp. and *P. sequeirae* n. sp.), five of which also found in the northerly, in the isolated Serra do Caparaó. In the parallel northern portion of Serra do Mar (including Serra dos Órgaos), seven species are also known to occur (*P. phanias, P. granulata, P. amussis*, *P. exul, P. duartei* n. sp., *P. francinii* n. sp. and *P. landryi* n. sp.), including five shared with the Mantiqueira range and two, *P. granulata* and *P. duartei* n. sp., which are found only along the coast. To the south (from Paraná to Rio Grande do Sul), seven species are also present, including three shared with the Serra da Mantiqueira (*P. phanias, P. landryi* n. sp. and *P. amussis*) two shared with the Serra do Mar (*P. granulata* and *P. duartei* n. sp.), *P. francinii* n. sp. (occurring in both Serra do Mar and Serra da Mantiqueira) and one restricted to southern Brazil, *P. zaccae* n. sp. In the southernmost portion of this range, the Serra Geral, six species are known, including *P. zaccae* n. sp. In the Serra dos Órgãos part of the Serra do Mar mountain range, only four species are known so far, *P. phanias*, *P. exul*, *P. landryi* n. sp. and *P. pawlaki* n. sp.; however, sampling has been rather limited there so far. One species of *Praepedaliodes* has been reported from the highlands of Belo Horizonte in the NW extreme of the Atlantic Forest, *P. landryi* n. sp. in the superficially sampled region of Serra do Cipó, in the south portion of the Espinhaço mountain range (G. C. N. Pereira, pers. comm.), and other additional species of *Praepedaliodes* are likely to be present (such as *P. phanias*, *P. francinii* n. sp. and *P. amussis*).

Distribution patterns of individual species are unlike those of most Andean cloud forest Pronophilina suggesting a different evolutionary history of *Praepedaliodes*. In the Andes, current topographical and ecological barriers generally constitute distributional limits for species, and higher and more elevated mountainous ranges often harbour several narrow range endemics. Examples are numerous among *Pedaliodes*, a genus closely related to *Praepedaliodes*. In the northern Andes, major valleys separating large mountainous chains such as the Cordillera de Merida, Sierra de Perijá, Sierra Nevada de Santa Marta or the Colombian Eastern, Central and Western Cordilleras prevent the dispersal of montane species and promote allopatric speciation (Adams 1985, 1986, Pyrcz & Rodríguez 2007, Pyrcz *et al* 2009, 2011). In the Brazilian Atlantic Mountains, there is no such obvious pattern. For example, all the species of *Praepedaliodes* occurring in Serra do Caparaó, a topographically and geographically isolated massif reaching high elevations, are also found in the Serra da Mantiqueira. These include even the species occurring at high elevations, generally at or above 2000 m, such as *P. pawlaki* n. sp. and *P. exul*. In addition, most species occurring in the latter range are found across the São Paulo basin in the Serra do Mar. The lowering of terrain in southern São Paulo and northern Paraná State, which separates the Serra de Mantiqueira from the Serra do Mar and the Serra Geral, does not seem to constitute any distributional barrier for *Praepedaliodes*. The only narrow endemic species among the *Praepedaliodes* is *P. sequeirae* n. sp., a taxon restricted to the high elevation in the Itatiaia massif in the Serra da Mantiqueira*.*

**Fig 29:**
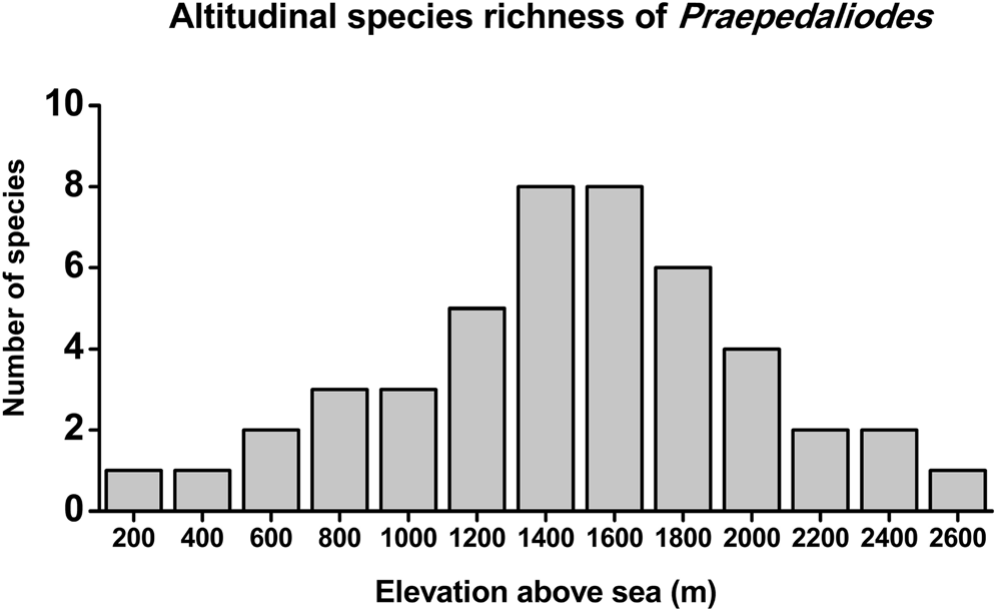
Altitudinal species richness of *Praepedaliodes*.

The genus *Praepedaliodes* reaches its highest diversity at mid to high elevations in Brazil; the peak species richness is reached at 1400–1600 m (Fig 29). However, there is no single area where actually such a species richness is actually attained, because some of the mid-elevation species are allopatric in the Serra de Mantiqueira and the Serra do Mar. Therefore, the highest single site diversity recorded is six species at 1800 m in the Serra da Mantiqueira. The lowest elevation reported for any member of *Praepedaliodes* is 10–200 m for *P. phanias* in Misiones (Argentina) and the neighbouring area of Brazil, and in southernmost Brazil (Pelotas) and Uruguay. The highest reported elevation is slightly above 2500 m for *P. sequeirae* n. sp. in the Itatiaia massif.

It is interesting to note that the abundance of all species of *Praepedaliodes* is typically low, in particular compared to the species of *Pedaliodes* and other related genera occurring in the Andes, such as *Panyapedaliodes, Altopedaliodes* and even some *Praepronophila* and *Pherepedaliodes* Forster, 1964. Despite the high abundance of host plants of the genus *Chusquea* in the montane cloud forests of SE Brazil, adults of *Praepedaliodes* are spatially restricted and are relatively uncommon in the field. This phenomenon certainly deserves some attention, but no working hypothesis can be advanced at this stage. Moreover, contrary to the Andean species of *Pedaliodes* complex, baits consisting of carrion or dung are ineffective and seldom attract individuals of *Praepedaliodes*. Again, quite unlike Andean *Pedaliodes*, adults of both sexes rather often visit flowers, particularly red, violet or pink ones, of various plants, preferably in the subcanopy (although *P. phanias* seldom visits flowers). Finally, pointing out some behavioural differences, species of the genus very rarely come to the ground to feed on decomposing matter or minerals, and are seldom attracted to fruit baits (except *P. phanias*, which is regularly sampled in fruit baited traps), as is commonly observed among Andean Pronophilina.

### Comparative taxonomy

Although the monophyly of *Praepedaliodes* is strongly supported by DNA based data, morphological evidence is less compelling. There are few salient synapomorphies in adult morphology that would support the separation of this genus from *Pedaliodes* complex.

While male genitalia have proven highly informative in the studies of the relationships of various groups of Neotropical Satyrinae, and in particular in assessing the affinities of the genera within the extremely species-rich *Pedaliodes* section, here, male genitalia of *Praepedaliodes* are of more limited systematic value. Male genitalia of *Praepedaliodes* present a series of diagnostic characters, in particular the shape of uncus (stout, curved downwards in the middle) and valva (with a spiny dorsum instead on single, prominent processes), whose combination allows identification with some confidence of a species as belonging to this genus. However, in particular, both the uncus (slender and straight) and the slender valva (with a smooth dorsum and one prominent process) of *P. amussis* are unlike all remaining *Praepedaliodes*, and based on genital morphology it is reminiscent of some Andean *Panyapedaliodes* (Fig 12e) rather than with *Praepedaliodes*, or indeed of *Pedaliodes* (sensu stricto) (Fig 12f). Nevertheless, despite these “aberrant” features of the male genitalia, *P. amussis* is clearly part of *Praepedaliodes* based on molecular evidence*.*

For the first time, the female genitalia of all species of *Praepedaliodes* were studied, and they showed several informative characters in evaluating infrageneric affinities within *Praepedaliodes*, but also in indicating its intrageneric relationships. In particular, all species of *Praepedaliodes* present a salient synapomorphy: large lateral pockets of proximal unit with rippled surface. These pockets are ventral extensions of the lamella antevaginalis, and although strongly sclerotized slat-like lamellas are present in *Pedaliodes* (sensu stricto), such additional structures have not been observed in any other taxa of *Pedaliodes* complex. The shape and structure of the surface of these pockets are highly species-specific, and indicate some possible closer affinities between the various species of *Praepedaliodes*. In particular, in *P. pawlaki* n. sp.*, P. exul, P. sequeirae* n. sp. and *P. zaccae* n. sp., the ripple pattern sculpturing the surface of the pockets is similarly shaped and directed. Both lateral pockets and lamella postvaginalis are similar in *P. granulata* and *P. francinii* n. sp. The lateral compression of the female genitalia is unique to *P. phanias* and *P. amussis*, as is the connection between lamellae ante- and postvaginalis. The ductus bursae is strongly sclerotized in all known species of *Praepedaliodes* as it is in several genera of the *Pedaliodes* complex, including *Corderopedaliodes* and *Physcopedaliodes* but not in *Panyapedaliodes* or in *Pedaliodes* (sensu stricto). However, only in *Praepedaliodes* is the presence of a bulbous sclerotization at the opening of ductus bursae into the corpus bursae is observed.

### Phylogeny and Evolutionary Pathways

This research, as well as previous phylogenetic studies, indicates that *Praepedaliodes* is related to other genera of *Pedaliodes* complex. The resolution of the molecular analysis data did not help identify with certainty the most closely related Andean clade of *Praepedaliodes*, as neither did previous works concerned with the phylogenies of Satyrinae (Peña *et al* 2006). DNA sequence data indicate a closer relationship to the clade of *Punapedaliodes* Forster and *Parapedaliodes* Forster, 1964. This result is intriguing, since the two extant species of *Punapedaliodes* are puna specialists restricted to high elevation habitats in Peru and Bolivia. These species therefore likely only recently radiated from ancestral *Pedaliodes* when boreal vegetation evolved in the Andes, a phenomenon which occurred in the late Pleistocene (Hooghiemstra & Van der Hammen 2004), while all species of *Praepedaliodes* are restricted to forest habitats (except for *P. sequeirae* n. sp. which occurs in the forest-grassland ecotone). Interestingly however, two species of a recently described Satyrinae genus *Stegosatyrus* Zacca, Mielke & Pyrcz, 2013 also occur in puna habitat in Peru and Bolivia, and in SE Brazil, although all the species of *Stegosatyrus* are grassland specialists (Zacca et al. 2013). These relationships suggest some biogeographical affinity between high elevation grassland Andean habitats and southeastern Brazil. In contrast, the other probably most closely related genus, *Parapedaliodes* is found at mid- to low elevations (below 1000 m) along the Pacific coast of the Andes in Peru and Ecuador. It occurs in dry forests and occasionally even in open meadows, and is not strictly associated with cloud forest bamboos as it also feeds on secondary Poaceae (Pelz 1997). It is therefore more prone to dispersal than most *Pedaliodes* as it does not require a dense cloud forest cover. The next most closely related genus in terms of genetic distances is *Physcopedaliodes*. This genus is monobasic and its only representative, *Physcopedaliodes physcoa*, occurs in central (*P. physcoa marulla* Thieme, 1905) and southern tropical and subtropical Andes (*P. physcoa micromaculata* Forster, 1964) (Pyrcz *et al* 2013). *Physcopedaliodes physcoa* is worth mentioning, as this is the only member of *Pedaliodes* complex that occurs at low elevations (below 500 m) and is actually found in the south-central Bolivian plains. Interestingly, during the austral summer *P. physcoa* flies at higher elevations, while during winter individuals are found across the plains, which suggests a tolerance to lower temperatures. On the other hand, a comparison of male and female genital morphology of *Praepedaliodes* with other genera of the *Pedaliodes* complex suggests that *Panyapedaliodes* may be the most closely related genus. A broader study with additional molecular markers is therefore needed to unveil the affinities of the genus *Praepedaliodes*.

Pleistocene paleoclimatic events had apparently little influence on *Praepedaliodes* divergence and speciation, contrary to what has been observed in the Andes (Casner & Pyrcz 2010, Pyrcz 2010, Pyrcz *et al* in prep.). The currently observed widely disjunct distributions of species such as *P. exul, P. landryi* n. sp. and *P. francinii* n. sp., found in isolated ranges such as the Serra do Caparaó, Serra de Mantiqueira, and in the latter two species, also the Serra Geral apparently has not had any noticeable effect on their phenotypic differentiation, and the cumulative effects of genetic drift are not observed in their genetic divergence.

Although the genus *Praepedaliodes* possibly evolved in the Atlantic Forest, the preliminary phylogenetic results clearly show that it is nested inside a large clade composed almost exclusively of Andean species. This suggests that the ancestor of *Praepedaliodes* arrived from the Andes dispersing through ecological corridors during cooler and more humid paleoclimatic periods, when suitable vegetation spread from the south-central Andes across the Chaco to connect with the south Brazilian highlands (van der Hammen & Hooghiemstra 2000). Species of *Praepedaliodes* strictly depend on the presence of species of *Chusquea*, their larval hostplants, and the current distribution of these small bamboos shows a gap (900 km) across the Chaco, possibly related to the low overall rainfall in that area. In addition, based on the preliminary phylogeny here presented, not only an Andean origin of *Praepedaliodes* is inferred, but also the clade that is sister to all remaining species in the genus includes *P. phanias*, the most widely widespread species in the genus, and *P. duartei*, the species most adapted to lowland habitats. However, only a study of character state distribution based on a strongly supported, more broadly sampled phylogeny will provide insights about the possible patterns of evolution and diversification in the genus *Praepedaliodes* (Magaldi *et al* in prep.).
